# Loop Dynamics and Enzyme Catalysis in Protein Tyrosine Phosphatases

**DOI:** 10.1021/jacs.0c11806

**Published:** 2021-03-04

**Authors:** Rory M. Crean, Michal Biler, Marc W. van der Kamp, Alvan C. Hengge, Shina C. L. Kamerlin

**Affiliations:** † Science for Life Laboratory, Department of Chemistry − BMC, Uppsala University, Box 576, S-751 23 Uppsala, Sweden; ‡ School of Biochemistry, University of Bristol, Biomedical Sciences Building, University Walk, Bristol BS8 1TD, United Kingdom; § Department of Chemistry and Biochemistry, Utah State University, Logan, Utah 84322-0300, United States

## Abstract

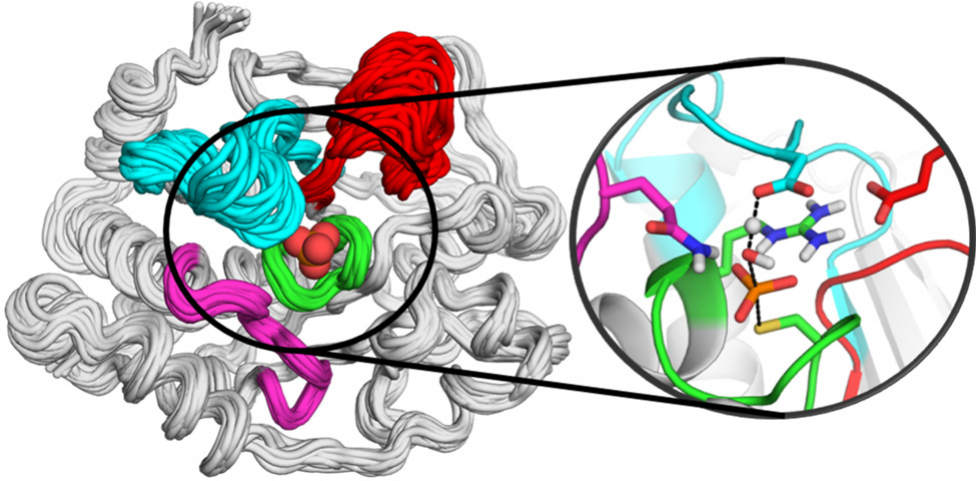

Protein tyrosine phosphatases (PTPs) play an important role in cellular signaling and have been implicated in human cancers, diabetes, and obesity. Despite shared catalytic mechanisms and transition states for the chemical steps of catalysis, catalytic rates within the PTP family vary over several orders of magnitude. These rate differences have been implied to arise from differing conformational dynamics of the closure of a protein loop, the WPD-loop, which carries a catalytically critical residue. The present work reports computational studies of the human protein tyrosine phosphatase 1B (PTP1B) and YopH from *Yersinia pestis*, for which NMR has demonstrated a link between their respective rates of WPD-loop motion and catalysis rates, which differ by an order of magnitude. We have performed detailed structural analysis, both conventional and enhanced sampling simulations of their loop dynamics, as well as empirical valence bond simulations of the chemical step of catalysis. These analyses revealed the key residues and structural features responsible for these differences, as well as the residues and pathways that facilitate allosteric communication in these enzymes. Curiously, our wild-type YopH simulations also identify a catalytically incompetent hyper-open conformation of its WPD-loop, sampled as a rare event, previously only experimentally observed in YopH-based chimeras. The effect of differences within the WPD-loop and its neighboring loops on the modulation of loop dynamics, as revealed in this work, may provide a facile means for the family of PTP enzymes to respond to environmental changes and regulate their catalytic activities.

## Introduction

**Figure 1:**
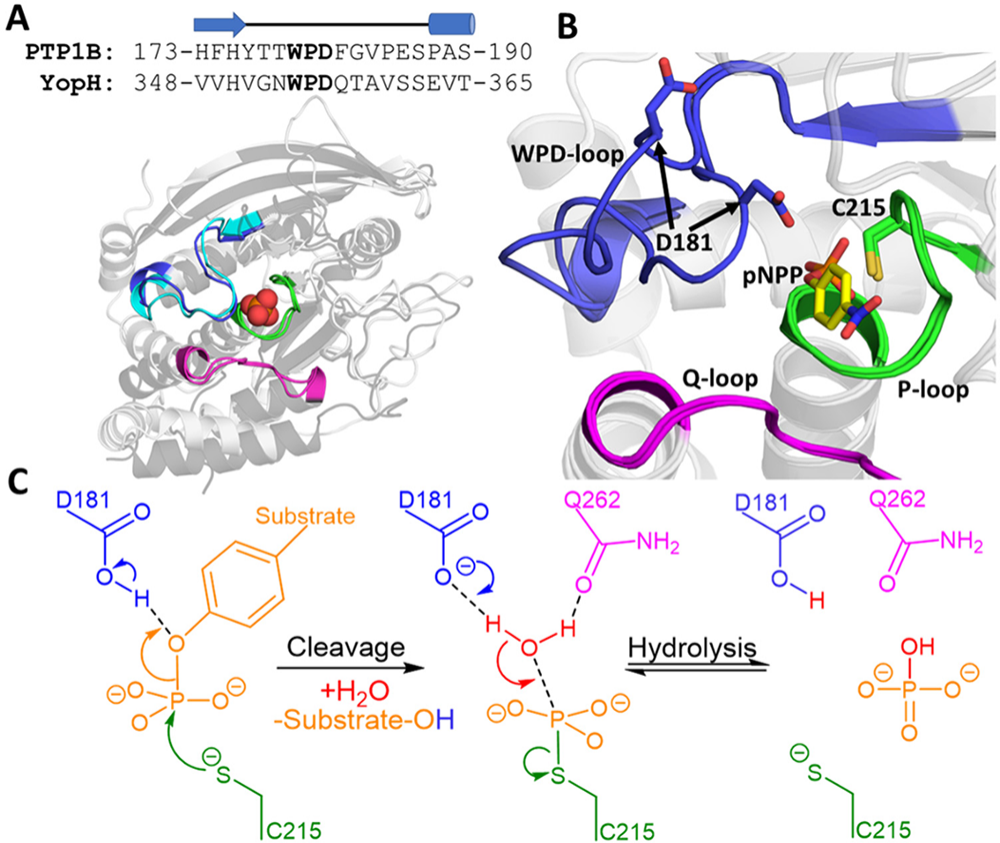
(A) Aligned crystal structures of the closed WPD-loop conformations of PTP1B (light gray, WPD-loop colored blue) and YopH (dark gray, WPD-loop colored cyan), with the phosphate binding site and key catalytic loops highlighted as annotated in Panel B. A sequence alignment of the WPD-loops of PTP1B and YopH is provided above the image, with the cartoon above the sequences used to indicate the secondary structure of each residue. (B) Close-up on the active site of PTP1B showing an overlay of the closed and open WPD-loop structures, with the model substrate *p*-nitrophenyl phosphate (*p*NPP) used in this study depicted in yellow (YopH structures not shown for clarity). (C) Generalized two-step reaction mechanism ascribed to PTPs, using the same coloring as in panel B to indicate the structural location of key residues. Structures of PTP1B (in both closed and open conformations) and YopH (closed conformation only) were taken from PDB IDs 6B90(6) and 2I42,^7^ respectively.

Protein tyrosine phosphatases (PTPs) are a superfamily of regulatory enzymes that play a key role in cellular signaling.^1^ As a result, these enzymes have been implicated in a wide variety of disorders, including type 2 diabetes^2^ and cancer,^3^ and have therefore been the subject of substantial biomedical research effort as potential drug targets.^4^ Members of this superfamily share a unique HCXXGXXRRS(T) “P-loop” signature motif at their active sites (HC(X_5_)R, where X is any residue). They catalyze dephosphorylation *via* a two-step “ping-pong” mechanism that is shared among PTPs (Figure 1), in which the thiol group of the conserved cysteine of the PTP signature motif (C215 using protein tyrosine phosphatase 1B, PTP1B, numbering) first acts as a nucleophile, in order to form a covalently bound thiophosphate enzyme intermediate, which is then hydrolyzed through nucleophilic attack by an active site water molecule.^5^ Further catalytic assistance is provided by the active site arginine present on the P-loop, R221, using PTP1B numbering. Furthermore, critical to this process is acid–base catalysis *via* a conserved aspartic acid, D181 in PTP1B, which acts as a general acid to protonate the leaving group in the first (cleavage) step of the PTP-catalyzed reaction and, subsequently, as a general base to activate the nucleophilic water molecule for the hydrolysis of the phospho-enzyme intermediate in the second step of the reaction mechanism. This aspartic acid lies on a conserved loop, the WPD-loop, which undergoes a large (∼10 Å) conformational change upon substrate binding, from a catalytically inactive “open” conformation to a catalytically active “closed” conformation, which brings this aspartate into position for acid/base catalysis (Figure 1). The nucleophilic water molecule in the second (hydrolysis) step is coordinated by the side chain of a glutamine residue located on the “Q-loop”, which in turn aids in optimally positioning this water molecule for nucleophilic attack on the phosphorus atom. (Figure 1C).

The dynamics of WPD-loop motion have been the subject of significant research effort.^6,8−14^ In particular, a detailed NMR study^9^ of two related PTPs, the human phosphatase PTP1B^1^ as well as YopH, a virulence factor from *Yersinia*,^15^ found an important role for the conformational dynamics of this loop in regulating phosphoryl transfer in these two enzymes, with rates of loop closure that mimicked the rates of phosphoester bond cleavage.^9^ Despite PTP1B and YopH having a relatively low sequence conservation (20.6% sequence identity as determined by the T-Coffee Web server^16^), they and other members of the PTP superfamily share highly superimposable active sites, the same mechanisms and rate-limiting chemical steps, and very similar transition states.^17^ This is significant because catalytic rates across the superfamily vary by several orders of magnitude.^14^ Further evidence for the role of loop motion in regulating PTP catalysis comes from NMR dynamics studies of several PTP1B point variants, in which a correlation was observed between the rate of loop opening/closure and the rates of both chemical steps.^18^

While YopH is the one of the most proficient PTPs,^19^ with *k*_cat_ values ≅1300 s^–1^ for the hydrolysis of monoester dianions at its pH optimum,^20^ PTP1B’s rate of catalysis is significantly slower, with *k*_cat_ values of ∼40 s^–1^ at its pH optimum.^21^ The *k*_cat_, or turnover number, reflects the rate-limiting step after formation of the Michaelis complex. The observation of burst kinetics in YopH^22^ and PTP1B^18^ show this is the second step in Figure 1. Finally, a combined kinetics, isotope effect, and X-ray crystallography study has shown that the precise molecular details of WPD-loop movement differ between PTP1B and YopH,^23^ as does the tolerance of the two loops to mutations, again strengthening the notion that differences in WPD-loop dynamics are important for regulating catalysis.

Despite extensive experimental and computational studies,^6,9,10,18,23,25,26^ it remains unclear why the catalytic rates of different members of the PTP superfamily are so different from each other, although the high structural conservation between these enzymes strongly points toward differences in the rates and dynamics of WPD-loop motion within the superfamily. This study focuses specifically on PTP1B and YopH, two of the best-characterized members of the PTP superfamily.^5,6,8−10,14,18,23,25−27^ In particular, our goal in this work is to understand why the rates of WPD-loop motions differ so significantly between the two enzymes, as well as exploring their correlation with the first chemical step of catalysis, as described experimentally in ref (9).

Furthermore, a recent study on WPD-loop chimeras of YopH and PTP1B (using the YopH scaffold with varying segments of the PTP1B WPD-loop sequence transposed in) demonstrated that some of these chimeras could adopt “hyper-open” WPD-loop conformations, in which the C-terminal portion of the WPD-loop is extended.^14^ While no crystal structures of the native enzymes show such conformations, it is unknown whether these conformations exist in solution and, if so, what, if any, functional relevance these hyper-open conformations have. In addition, while it is clear that WPD-loop closure is essential for the rapid first step of catalysis, it is unclear whether further protein motions occur later in the mechanism.

We note that, while both enzymes have been the subject of significant computational work, in particular for drug discovery efforts, advanced studies of their loop dynamics have been limited in scope,^8,10,28^ and there currently exists no comparative computational study of the two systems. To this end, in the present study, we have performed extensive conventional molecular dynamics (MD) simulations coupled with advanced enhanced sampling simulations. Using Hamiltonian replica exchange^29^ and metadynamics simulations, we characterized the free energy landscapes of loop motion in both enzymes and how these loop dynamics are regulated by the remainder of the protein scaffold. We further complement our approach by performing empirical valence bond (EVB)^30,31^ simulations on both chemical steps of catalysis to provide insight into the link between loop dynamics and the regulation of the phosphoryl transfer reaction catalyzed by these enzymes.

Our simulations illustrate clear differences in the mobilities of both PTPs’ WPD-loops, with YopH’s WPD-loop being substantially more flexible than PTP1B’s, in part due to two proline hinges present in PTP1B. We also identified conserved allosteric communication pathways present in both PTPs that help to regulate WPD-loop motion. Further, we observed wild-type YopH to adopt an additional catalytically incompetent “hyper-open” conformation and pinpointed the structural features that explain why YopH can adopt this conformation while PTP1B cannot.

In stark contrast to the differences in loop dynamics, our EVB simulations suggest that there is no intrinsic difference in the activation free energies for the rate-limiting hydrolysis steps (Figure 1C) of the reactions catalyzed by PTP1B and YopH, respectively. Rather, the experimentally observed differences in the turnover number^20,21^ are most likely due to the experimentally observed differences in the loop dynamics between the two enzymes.^9^ Our simulations also highlight the importance of the conformational sampling of a second loop, the so-called “E-loop”, which is spatially adjacent and correlated to the motions of the WPD-loop for both PTPs. In PTP1B, the E-loop is highly mobile and contains many key residues for transition state (TS) (de)stabilization (as identified by our EVB simulations), suggesting that the “correct” conformational sampling of this loop is vital for catalytic activity. In contrast, YopH possess a highly rigid E-loop, which has a substantially less pronounced role in TS (de)stabilization. Taken together, our study provides key insights into both the dynamical and chemical aspects of PTP catalysis, as well as valuable input for future drug discovery and enzyme engineering efforts on these biomedically critical enzymes.

## Methodology

Methodological details here are presented in brief, with a full description of the simulation details and methods used presented in the Supporting Information.

### System Preparation for Conventional and Enhanced Sampling Molecular Dynamics Simulations

A total of six crystal structures^5−7,32,33^ were used in this study to generate the starting points for simulations of the unliganded forms, Michaelis complexes with *p*-nitrophenyl phosphate (*p*NPP), and covalent phospho-enzyme intermediates for PTP1B and YopH, with their WPD-loops in both their open and closed conformations (see Tables S1 and S2). Simulations of the Michaelis complexes were performed with the WPD-loop acid protonated, while simulations of the unliganded enzyme and the phospho-enzyme intermediate were performed with the WPD-loop acid deprotonated. Where chemically relevant, the cysteine nucleophile was simulated in its deprotonated form. Protonation and tautomerization states of all other residues were kept consistent for all simulations using PROPKA^34^ v3.1, the MolProbity^35^ server, and visual inspection (the assignments used are provided in Table S3). For all conventional and enhanced sampling MD simulations, structures were simulated using periodic boundary conditions. Partial charges for *p*NPP and the phosphorylated cysteine residue (both modeled in their dianion forms) were calculated using the standard restrained electrostatic potential (RESP) protocol (HF/6-31G(d)), using Antechamber.^36^ For *p*NPP, all other simulation parameters were described using the general Amber force field 2 (GAFF2) (see Table S4).^37^ For the phosphorylated cysteine residue, parameters were taken directly from the ff14SB^38^ force field where possible, with any missing terms obtained from GAFF2^37^ (see Table S5).

### Conventional Molecular Dynamics Simulations

Conventional MD simulations were performed using Amber16,^39^ with the protein and water molecules described using the ff14SB^38^ force field and the TIP3P^40^ water model, respectively. Simulations of all 12 different systems of interest here (unliganded, *p*NPP-bound Michaelis complexes and phospho-enzyme intermediates, starting from both open and closed conformations of the WPD-loops of both PTP1B and YopH) were performed for 25 × 200 ns each, in the NPT (300 K, 1 atm) ensemble. Following system equilibration (see the Supporting Information), MD simulations were performed using a 2 fs time step, with the SHAKE algorithm.^41^ One sided harmonic restraints were used to maintain the *p*NPP substrate in a catalytically competent configuration throughout the MD simulations in order to study the interaction between the WPD-loop and the bound substrate (see Table S6 for the restraints applied). We note that equivalent restraints were put in place for both PTPs and no restraints were placed between *p*NPP and the WPD-loop to ensure full conformational freedom of this loop.

### Hamiltonian Replica Exchange Molecular Dynamics Simulations

HREX-MD^29^ simulations were performed on the unliganded forms of both PTPs using the Amber ff99SB-ILDN^42^ force field and TIP3P^40^ water model as implemented into GROMACS 2018.4,^43^ interfaced with PLUMED v2.5^44^ (the ff99SB-ILDN was chosen over ff14SB as only force fields embedded into GROMACS can be used for this simulation methodology). Following system equilibration (see the Supporting Information), two 500 ns long HREX-MD simulations were performed for PTP1B and YopH each, with one simulation starting from the WPD-loop closed structure and the other with the WPD-loop open. All HREX-MD simulations were performed in the NPT ensemble, using a 2 fs time step and the P-LINCS algorithm^45^ to restrain all bonds to hydrogen atoms.

A generous definition of the WPD-loop was used to define the residues included in the “hot region” of the simulations (i.e., residues 175–191 and 349–365 for PTP1B and YopH, respectively). Simulations were performed using a total of eight replicas, with λ values exponentially scaled between 1.0 and 0.6 and exchanges attempted every 1 ps, achieving an average exchange rate of ∼40% for both PTP1B and YopH. Subsequent analysis was performed solely on the neutral replicas (λ = 1).

### Parallel Tempering Metadynamics Simulations

Parallel tempering metadynamics simulations performed in the well-tempered ensemble (PT-MetaD-WTE)^46−48^ were performed with GROMACS 2018.4,^43^ interfaced with PLUMED v2.5,^44^ using the Amber ff14SB^38^ force field and the TIP3P^40^ water model. Following equilibration of each replica to its target temperature, PT-MetaD-WTE simulations were performed in two stages on all of the unliganded, *p*NPP-bound, and phospho-enzyme intermediate states of both PTP1B and YopH. First, a 10 ns long PT-MetaD simulation was performed with a bias potential placed on the potential energy of the system. In the second step, the bias on the potential energy was retained but no additional Gaussians were deposited onto this CV. Instead, three CVs were chosen to describe WPD-loop motion for the production PT-MetaD-WTE simulations. CV1 describes the interloop distance-root-mean-square deviation (DRMSD) between the C_α_ atoms of the WPD- and P-loops, with the closed crystal structures used as the reference structure. CV2 and CV3 describe the motions in the central and C-terminal portions of the WPD-loop, respectively, through a center of mass distance measurement between the WPD-loop residues to atoms on the P- or Q-loops (which are highly rigid). The initial Gaussian height was 0.2 kcal mol^–1^, with a deposition rate of 2 ps and bias factor of 12. Wall potentials were used to prevent the sampling of non-relevant states. Simulations were run for between 700 and 800 ns per replica, with convergence assessed by monitoring the time evolution of the free energy profiles (Figures S1 and S2), alongside checking for diffusive dynamics along each CV (Figures S3 and S4). The CVs used to describe WPD-loop motion are shown in Figure S5 and Tables S7 and S8. Analysis was performed on the replica simulated at 300 K, and simulations were reweighted and projected onto unbiased CVs using the approach described by Tiwary and Parrinello.^49^ The minimum free energy pathway (MFEP) was determined using MEPSA.^50^

### Analysis of Conventional and Enhanced Sampling Molecular Dynamics Simulations

Unless stated otherwise, all analysis of all conventional and enhanced sampling molecular dynamics simulations was performed using CPPTRAJ.^51^ Hydrogen bonds were defined as formed if the donor–acceptor distance was ≤3.5 Å and the donor-hydrogen–acceptor angle was within 180 ± 45°. Principal component analysis (PCA) on the HREX-MD simulations was performed by first RMS fitting to a stable region of the enzyme and then performing PCA on the C_α_ carbons of the WPD-loop. Dynamic cross correlation matrixes (DCCMs) and average inter-residue distance matrixes of the PT-MetaD-WTE trajectories were computed for the C_α_ of every residue. Shortest path maps (SPMs)^52^ were determined using the DCCM and average inter-residue distance matrixes for the simulations of PTP1B and YopH with *p*NPP-bound to the active site, using the available Python script.

### Empirical Valence Bond Simulations

The EVB^30^ approach has been used extensively to describe phosphoryl transfer reactions and loop dynamics,^24,53−58^ including in computational studies of PTP mechanisms.^55,56^ In this work, we have performed EVB simulations of both chemical steps of the reactions catalyzed by PTP1B and YopH (Figure 1). Our starting points for these simulations were PDB IDs 3I7Z(5) and 1QZ0(33) to describe the cleavage step and 3I80(5) and 2I42(7) to describe the hydrolysis step in the reactions catalyzed by PTP1B and YopH, respectively.

In brief, the system preparation and initial equilibration for EVB simulations were performed as described in the Supporting Information. Each system/reaction step was simulated using 30 individual replicas. Each replica was first equilibrated for 30 ns at the approximate transition state (λ = 0.5), with the subsequent EVB trajectories propagated downhill from the transition state in both the reactant and product directions, following our previous work.^54,57^ Each EVB simulation was performed in 51 individual mapping windows of 200 ps in length per trajectory. This led to total cumulative equilibration and EVB simulation time scales of 1.8 and 0.612 μs per enzyme over all individual replicas and both reaction steps (cleavage and hydrolysis), respectively. We note that the active site microenvironment in PTPs causes a substantial reduction in the p*K*_a_ of the catalytic cysteine relative to free cysteine in solution, to experimentally determined values of 4.67 in YopH^59^ and 5.6 in a related PTP, vaccinia H1-related PTP (VHR).^60^ This means that no thermodynamic correction would need to be applied for the deprotonation of the active site cysteine as the deprotonated form will dominate at ambient pH.

All EVB simulations were performed using the *Q6* simulation package^61^ and the OPLS-AA^62^ force field, for consistency with previous related studies.^53,54,63,64^ All EVB parameters necessary to reproduce our work, as well as a detailed description of the computational methodology and subsequent simulation analysis can be found in the Supporting Information.

## Results and Discussion

### Analysis of Available Experimental Structural Information

**Figure 2:**
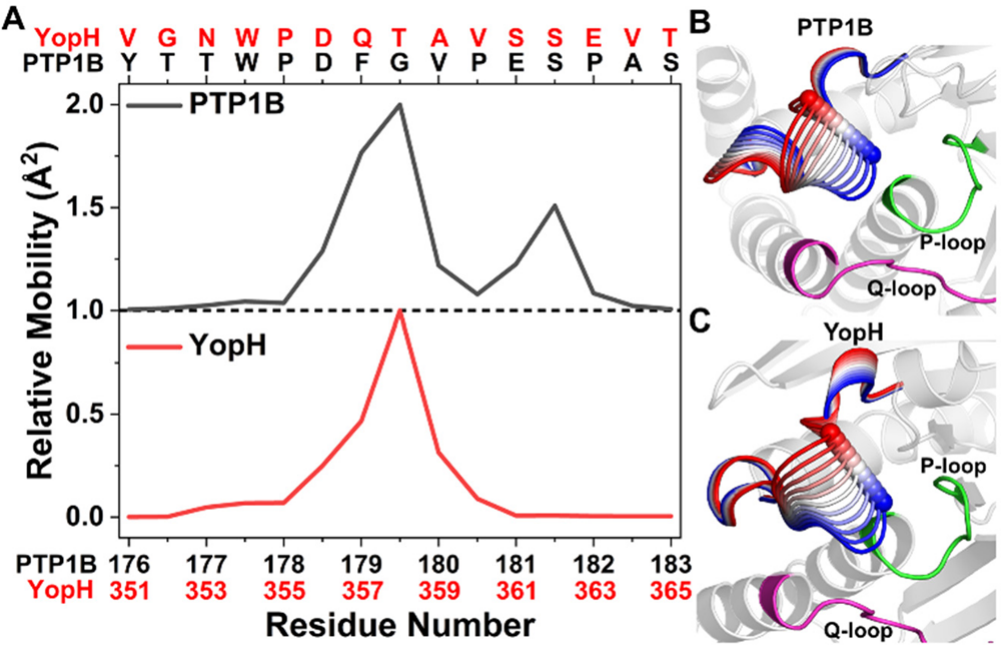
Principal component analysis (PCA) on the WPD-loops of the crystal structures of PTP1B and YopH. (A) Relative mobility of each residue in the projection of PC1, with residue numbers and names provided above and below, respectively, for both PTPs. (B and C) Projection of PC1 onto representative structures of (B) PTP1B and (C) YopH. The color gradient on the WPD-loop indicates the transition from an open (red) to a closed (blue) WPD-loop conformation along PC1 (see Figure S7 for projections onto each structure). The other key catalytic loops are labeled as in Figure 1.

In order to lay the groundwork for our subsequent simulations, we performed a detailed analysis of the conformational diversity of the WPD-loops of PTP1B and YopH on the basis of structures available in the PDB (using 251 structures in total, see the Supporting Information for details).^65^ Our focus was on distinguishing between the differences in the closed and open conformations of the loop for both PTPs using principal component analysis (PCA). PCA on the WPD-loops of the structures of PTP1B and YopH was performed separately, and for both enzymes, we observed the first PC to correspond to loop opening/closure (Figure 2), and to be able to describe the large majority of the variance between the different structures (greater than 91% in both cases, see Figure S6). Further analysis is presented in Section S2 of the Supporting Information.

A comparison of the mobility plots for the individual PC1 projection data (Figure 2A) shows that the majority of changes in both WPD-loops are focused on the central portion of the WPD-loop. However, PTP1B shows a smaller second peak toward the C-terminus of the WPD-loop, which is of particular interest in light of the fact that both residues in this second peak (E186 and S187) flank proline residues and are therefore likely to have restricted mobility. These insights may provide some rationale as to why the movement of the WPD-loop of YopH is so much faster than that of PTP1B.^9^ That is, the bimodal distribution of mobility over multiple PTP1B residues (as opposed to the monomodal distribution observed in YopH), combined with the increased number of pre- or postproline residues that show significant mobility over PC1 (describing loop motion, of which there are three in PTP1B and only one in YopH), would point toward a likely slower loop motion in PTP1B than in YopH.

### Hamiltonian Replica Exchange Molecular Dynamics Simulations

**Figure 3:**
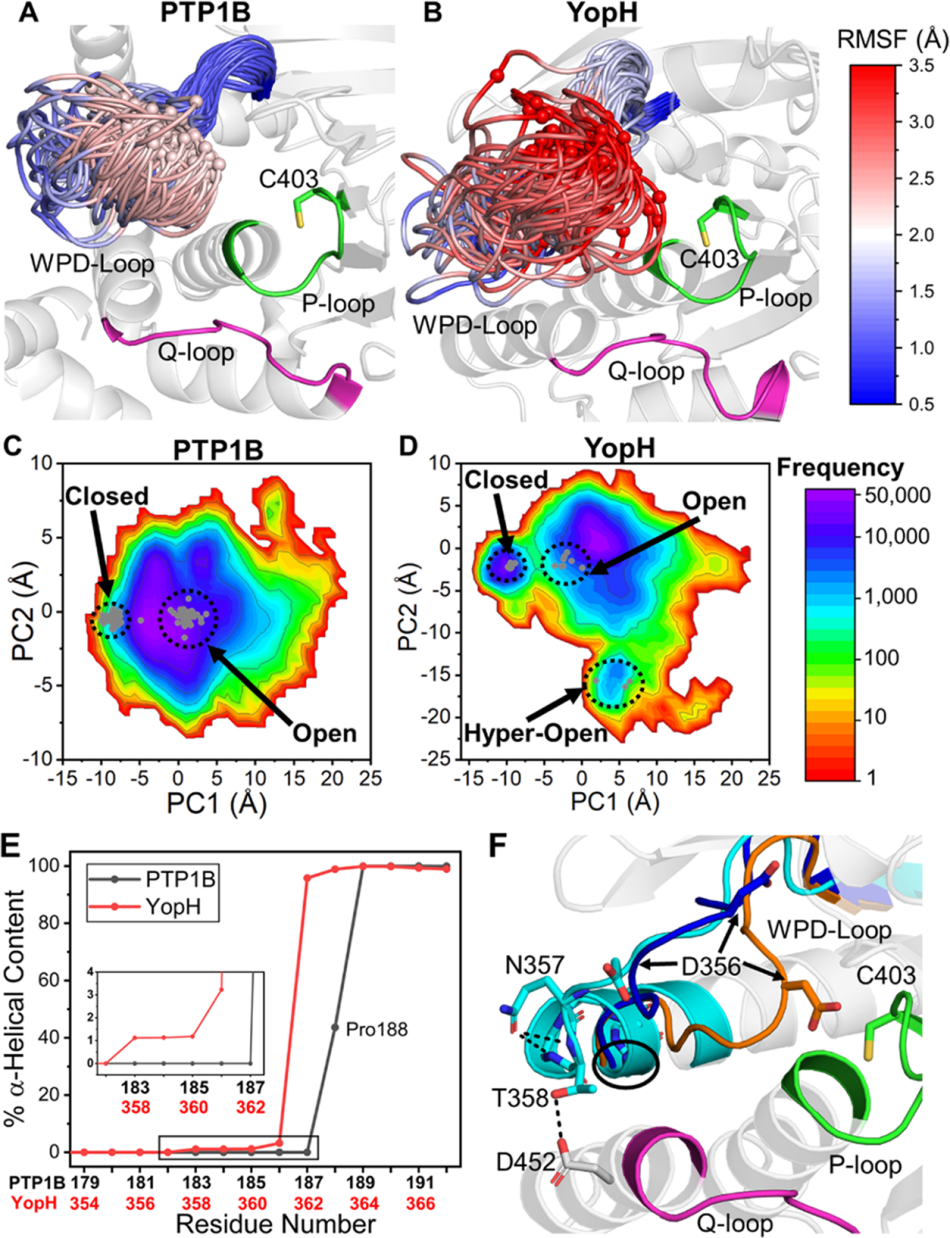
Extensive sampling of the free energy landscapes of the WPD-loops of PTP1B and YopH using HREX-MD simulations. (A and B) Snapshots from the HREX-MD simulations of (A) PTP1B and (B) YopH, showing the diverse conformations sampled by the WPD-loop during the simulations. The WPD-loop residues are colored from red (most flexible) through white and to blue (least flexible) according to their calculated C_α_ RMSF (shown in graphical form in Figure S8). This shows that the WPD-loop in YopH is more flexible and samples a broader range of conformational space than that of PTP1B. The catalytic Asp on the WPD-loop is shown as a sphere on this plot for reference. (C and D) 2D histograms of the first two PCs of the WPD-loop for (C) PTP1B and (D) YopH using a natural log scale. The corresponding X-ray crystal structures are projected onto each plot as small gray dots, with structures corresponding to closed, open, and hyper-open states indicated. (E) Percentage of snapshots that are in an α-helical configuration for the WPD-loops and subsequent α-helices of PTP1B and YopH. (F) Representative structure of the hyper-open conformation of the WPD-loop adopted by YopH in which residues up to T358 are in an α-helical conformation. The hyper-open, open, and closed states are colored cyan, dark blue, and orange, respectively. Two key interactions that help stabilize this configuration are shown (see main text for further details).

Despite the fact that our PCA (which was performed on a broad range of crystal structures) suggests that the differences in dynamics of the WPD-loops of both PTP1B and YopH can be well described by a single PC (i.e., a vector), this does not mean that the conformational change from the open-to-closed conformations of the loop is simple in solution. That is, we have previously shown that, even in the textbook example of the closure of the catalytic loop of triosephosphate isomerase,^54^ which has been often argued to occur as a two-state rigid-body loop motion,^66−68^ the loop dynamics are complex, exhibiting high flexibility and sampling of multiple conformational substates. Although flexibility in mobile loops seems to be the norm, the small size of the WPD-loops provides a test of whether rigid body motion occurs in small mobile loops. Therefore, to further explore the loop dynamics of PTP1B and YopH, we turned to enhanced sampling molecular dynamics (MD) simulations, including HREX-MD simulations of WPD-loop motion in the unliganded-enzyme forms of PTP1B and YopH (8 μs simulation time per PTP, see the Methodology section). A comparison of the conformations sampled by both enzymes’ WPD-loops in our HREX-MD simulations suggests that the WPD-loop of YopH has both a higher mobility and/or a larger accessible conformational space than PTP1B (Figure 3A,B), which is also supported by C_α_ root-mean-square fluctuations (RMSF) calculations (Figure S8).

In order to characterize the WPD-loop conformations sampled during our HREX-MD simulations, we performed PCA on each PTP individually, projecting the observed populations along PCs 1 and 2 as a 2D histogram (Figure 3C,D). PC1 for both PTP1B and YopH describes WPD-loop opening/closure as evidenced by the groupings of closed and open X-ray structures along PC1 (Figure 3C,D). It is also clear that both PTPs can sample a wide variety of open conformations, which can also be notably “more open” (i.e., further from the crystal structure closed conformation) than the open crystal structures.

Interestingly, as we observed in the structural PCA performed on the WPD-loop residues (Figure 2A), the mobilities obtained for PC1 from the HREX-MD data also show a clear monomodal vs. bimodal distribution of mobilities for the WPD-loop residues of YopH and PTP1B, respectively (Figure S9). PC2 in PTP1B corresponds to changes in the central and N-terminal WPD-loop residues, while PC2 in YopH describes a broader movement of the WPD-loop from the open state to another minimum, referred to as the “hyper-open” conformation.^14^ In this hyper-open conformation, the α-helix connected to the C-terminal portion of the WPD-loop is extended by four residues (normally beginning at S362 in the WT closed and open states, in contrast with T358 in the hyper-open conformation, see Figure 3E,F) and has previously only been observed in the crystal structures of two YopH–PTP1B chimeras, in which the YopH WPD-loop is partially swapped for that of PTP1B.^14^

These results therefore suggest that this hyper-open loop conformation is already sampled in the WT-YopH structure, albeit as a rare event (only ∼1.1% of simulation time as determined from analysis of the % α-helical content of the WPD-loop residues, see Figure 3E). The WPD-loops in the two hyper-open crystal structures (PDB IDs: 6DR1(14) and 6DT6(14)) slightly differ from one another (WPD-loop backbone RMSD of 1.38 Å), and our simulations of WT-YopH show that both crystallographically observed hyper-open conformations can be readily sampled (Figure S10). Specifically, the snapshot with the lowest RMSD to each PDB has RMSDs of 0.95 and 1.03 Å to PDB IDs 6DR1(14) and 6DT6,^14^ respectively. Finally, we calculated how the hydrogen bonding network of the WPD-loop differs for the closed, open, and hyper-open states of YopH (Figure S11). Alongside the new interhelical hydrogen bonds formed through the extended α-helix, the side chain carbonyl of the Q357 acts to cap the positive dipole at the end of the helix and a high occupancy hydrogen bond was found between T358 and D452 on the α6-helix (as depicted in Figure 3F).

A comparison of the WPD-loop sequences (see Figure 1A) helps to rationalize why WT-YopH but not WT-PTP1B appears to be able to adopt this hyper-open conformation. That is, P188 in PTP1B (equivalent to E363 in YopH) likely acts as a “helix-breaker”, preventing the extension of the α-helix beyond residue S187 as seen in our simulations of WT-PTP1B (see Figure 3E). The lack of a proline at this position of the WPD-loop in YopH (and in both chimeras crystallized with hyper-open conformations^14^) may therefore provide the necessary conformational flexibility to form this extended helix conformation. It is interesting to note that a recent NMR dynamics study of the PTP1B point variant P188A identified two exchange processes for the WPD-loop as opposed to one for WT-PTP1B and the four other point variants included in the study.^18^ Taken together, our simulations suggest that this second/new process identified in the P188A PTP1B variant may correspond to conformational exchange between the open and (now accessible) hyper-open states of the WPD-loop.

### Parallel Tempering Metadynamics Simulations

As we previously observed in the enzyme triosephosphate isomerase,^54^ our HREX-MD simulations were unable to extensively exchange between the closed and open states of the WPD-loop (see Section S2 of the Supporting Information for further details). This meant that we were unable to obtain a reliable picture of the energy differences between the different states using HREX-MD. We therefore turned to parallel tempering metadynamics simulations in the well-tempered ensemble (PT-MetaD-WTE),^47,48^ simulating the unliganded, *p*NPP-bound Michaelis complexes, and phospho-enzyme intermediate states of both PTP1B and YopH. PT-MetaD-WTE (which combines temperature-based replica exchange with metadynamics simulations) is a particularly useful method for sampling complex reactions coordinates such as the protein conformational change simulated here.^69−71^

**Figure 4:**
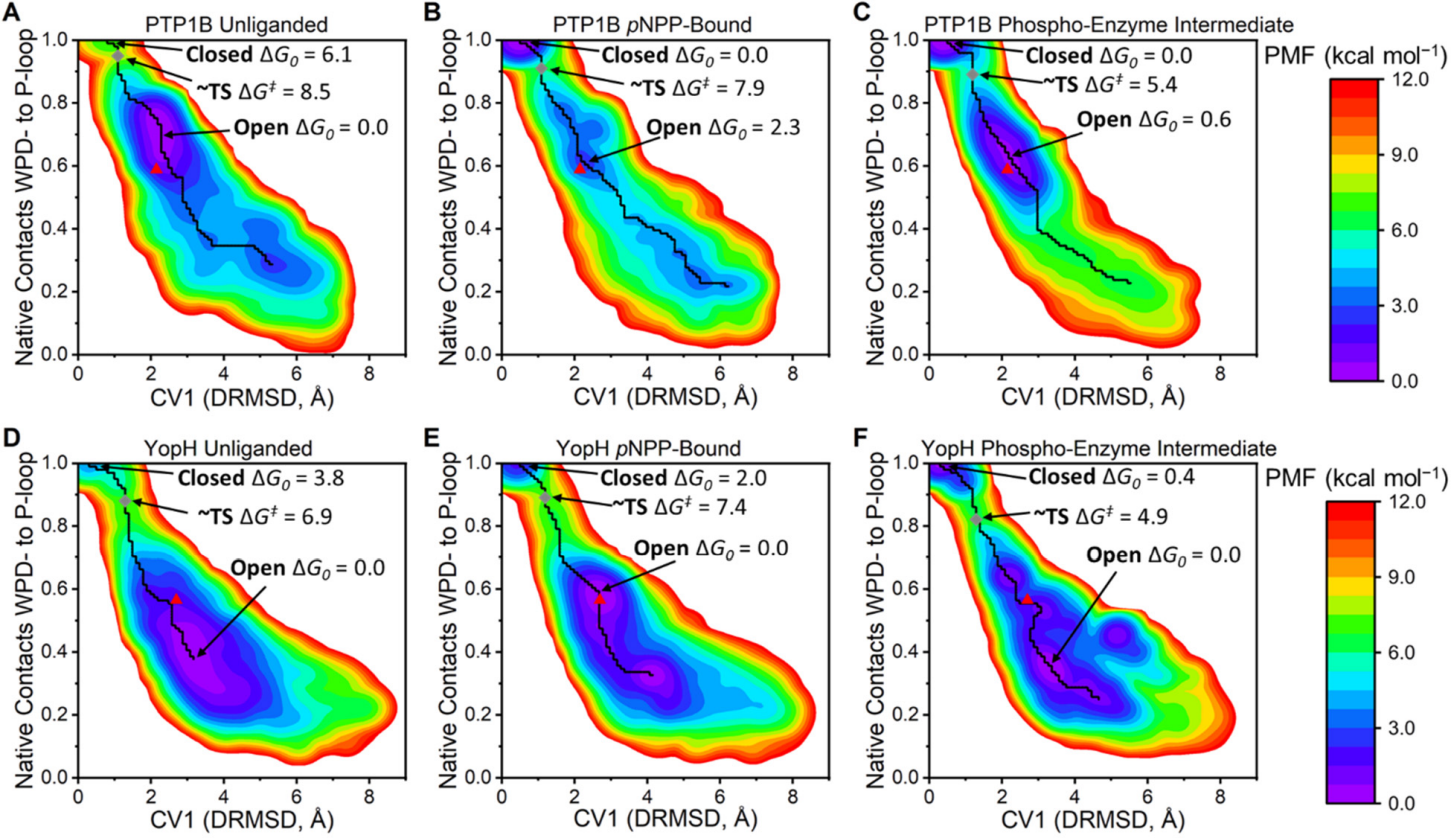
Changes in the WPD-loop free energy landscape over the catalytic cycle of PTP1B and YopH as determined by our PT-MetaD-WTE simulations. (A–C) PMFs of the unliganded, *p*NPP-bound, and phospho-enzyme intermediate states of PTP1B and (D–F) those for YopH. The *x*-axis is the metadynamics simulations’ collective variable 1 (CV1), which is the interdistance RMSD (DRMSD) between the WPD-loop and P-loop C_α_ carbons (using the closed conformation as the reference state). The *y*-axis is the fraction of native contacts^72^ formed between the C_α_ carbons of the WPD-loop and P-loop (values decreasing from one mean a move away from the reference state, the closed X-ray structure, defined in detail in the Supporting Information). In all cases, the minimum free energy pathway (MFEP) between the closed and open states is plotted as a black line. The minimum energy values of the closed and open states are indicated, alongside the size of the pseudo TS barrier (gray diamond) between the two states. The representative X-ray structure of the open (PTP1B, 6B90(6) and YopH, 1YPT(32)) conformation is shown as a red triangle. Note that the closed state (PTP1B, 6B90(6) and YopH, 2I42(7)) has the coordinates (0,1) and that PDB ID 6B90(6) contains both a closed and open WPD-loop conformation.

After analysis of the time evolution of the free energy profiles and diffusive dynamics along each CV to confirm simulation convergence (see the Methodology section), we reweighted and projected the free energy landscapes obtained onto the interdistance RMSD between the WPD- and P-loops (i.e., CV1) and the fraction of native contacts^72^ between the WPD- and P-loops (Figure 4). This allowed us to clearly distinguish between the closed and open states of the WPD-loop alongside constructing a minimum free energy pathway (MFEP) between both states. We caution that the transition state (TS) barrier obtained for complex conformational changes like this is highly sensitive to the reaction coordinate(s) used and should be considered as an approximation of the TS.^73,74^ Instead, in the following sections, we used our obtained MFEPs to describe the structural features along the loop opening/closing process.

Consistent with our HREX-MD simulations, both PTP1B and YopH (in all three states) can sample a broad range of conformational space, including conformations notably more open than their corresponding “open” X-ray crystal structure. Further, our simulations show a clear population shift toward the closed state when *p*NPP is bound or in the phospho-enzyme intermediate state. These results are consistent with prior NMR and crystal structure data,^9^ in which substrate binding or the presence of a phosphate group mimic (to mimic the phospho-enzyme intermediate state) shifted the WPD-loop equilibrium toward favoring the loop closed state for both PTPs. We note that, while for YopH with *p*NPP-bound, the open state is still energetically preferred, there has nonetheless been a clear population shift toward the closed state when compared to unliganded YopH. Finally, we analyzed our simulations for potential changes in the p*K*_a_s of all titratable residues along the WPD-loop opening/closing pathway but found no substantial changes for any residue (see Section S2 of the Supporting Information for further details).

**Figure 5:**
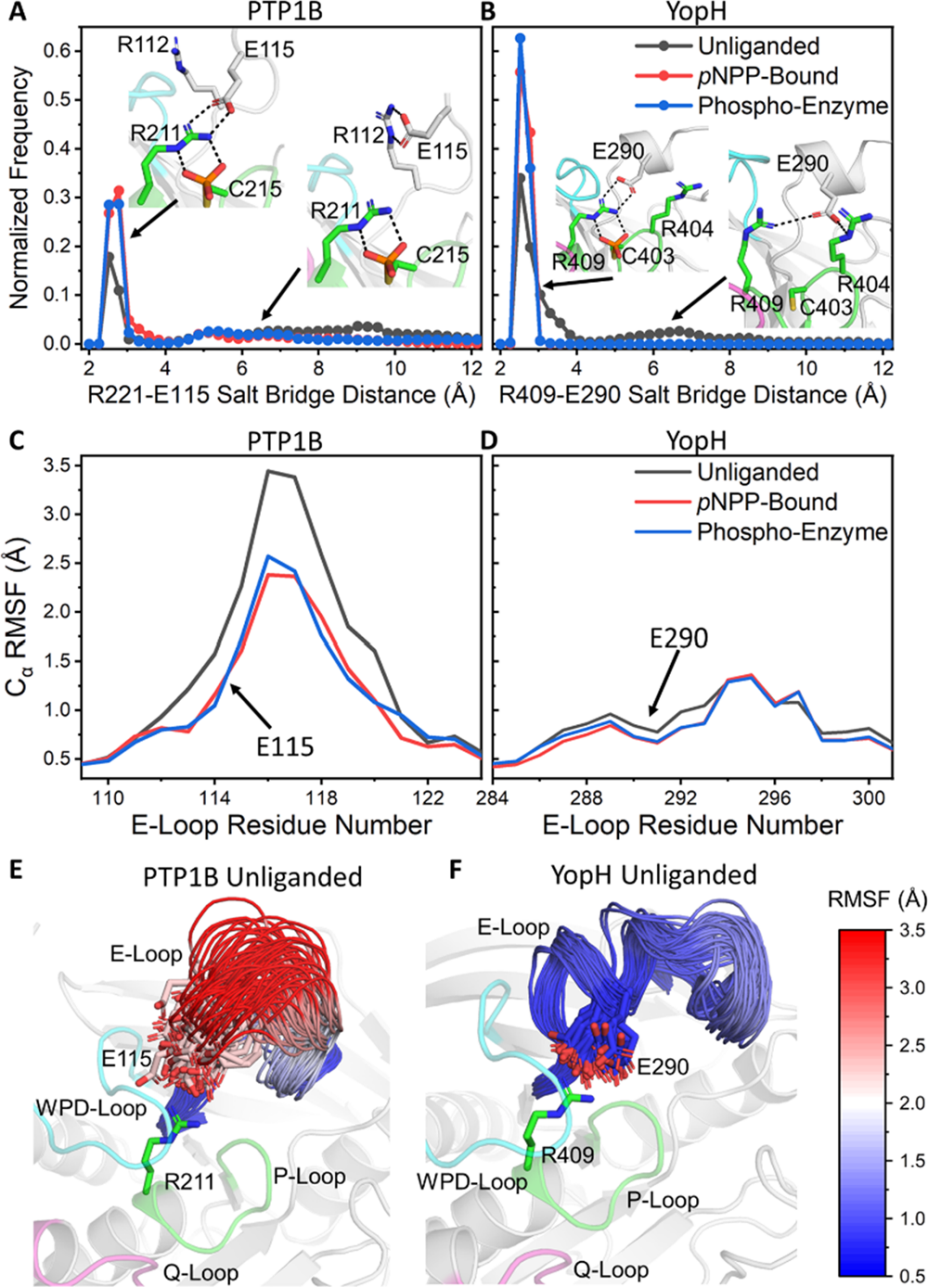
Differences in the structural stability of the E-loops of PTP1B and YopH from our PT-MetaD-WTE simulations. (A and B) Normalized histograms of the P-Loop arginine to the E-loop glutamic acid salt bridge distance from each our simulations of (A) PTP1B and (B) YopH for the unliganded, *p*NPP-bound, and phospho-enzyme intermediates states of both enzymes. The figures show representative conformations for each protein with and without the salt bridge formed. (C and D) RMSFs of the C_α_ atoms of the E-loop residues, obtained from our PT-MetaD-WTE simulations of (C) PTP1B and (D) YopH in all three states simulated. (E and F) Representative structures of the conformational sampling/diversity of the E-loops of (E) PTP1B and (F) YopH, in the unliganded states of each enzyme. E-loop residues are colored mapped from red (most flexible) through white and to blue (least flexible) according to their calculated C_α_ RMSF.

We further analyzed our PT-MetaD-WTE simulations in order to understand what drives the observed population shift (Figure 4) toward the closed WPD-loop conformation for both PTPs when bound to either the substrate or when in the phospho-enzyme intermediate state. One driving force will be from direct interactions between either the substrate or the thiol-phosphate group and the side chain of the WPD-loop Asp when in the closed state (see e.g., Figure 1). Our simulations, however, identify a secondary and indirect mechanism by which the binding of a phosphate group induces a population shift toward the closed state through the preorganization of the E-loop toward a productive WPD-loop closed state (Figure 5). That is, for both PTPs, the E-loop and P-loop are coordinated to one another through a highly evolutionarily conserved (among PTPs, Figure S12) salt bridge between the Arg residue on the P-Loop and the Glu residue on the E-loop (R211/E115 and R409/E290 for PTP1B and YopH, respectively). This P-loop Arg is responsible for coordinating the reacting phosphate group (Figure 5A,B), with the E-loop Asp being responsible for locking the Arg side chain into its catalytic configuration. For both PTPs, we observed the binding of a phosphate group (either from the substrate or in the phospho-enzyme intermediate state) to stabilize this salt bridge (Figure 5A,B) and ultimately rigidify the E-loop (Figure 5C,D). This also helps to induce a population shift toward the closed state by preventing the P-Loop arginine from sampling side chain rotamers that would block productive WPD-loop closure but would not impact the sampling of the open WPD-loop conformation.

From comparing the relative structural stabilities of the E-loops of PTP1B and YopH (Figure 5C–F), it is clear that the E-loop of YopH is notably more stable than that of PTP1B and even contains some degree of a secondary (α-helical) structure. Further, in the case of YopH, we observed no instances of the salt bridge breaking when a phosphate group was present, while for PTP1B in all three simulated states, the salt bridge was observed to be broken for at least some of the simulation time (Figure 5A,B). Our results show that, even with a bound phosphate group, the PTP1B E-loop can still undergo large conformational changes to break the salt bridge (Figure 5A,E). These observations are particularly noteworthy given that a recent NMR dynamics study has suggested that the observed *k*_cat_ for PTP1B does not reflect the isolated open to closed transition of the WPD-loop but one in which this motion occurs in concert with other, cooperative fluctuations, involving the E-loop in particular.^11^ To check for such coupled motions between the WPD-loop and E-loop in our simulations, we computed the dynamic cross correlation matrixes (DCCMs) for both PTPs (Figures S13 and S14). Consistent with the above NMR experiments, we identified the E-loop (and Q-loop) to be notably correlated with WPD-loop motion (see Section S2 of the Supporting Information for a detailed analysis of correlated motions).

### Identification of Key Allosteric Communication Pathways and Residues

**Figure 6:**
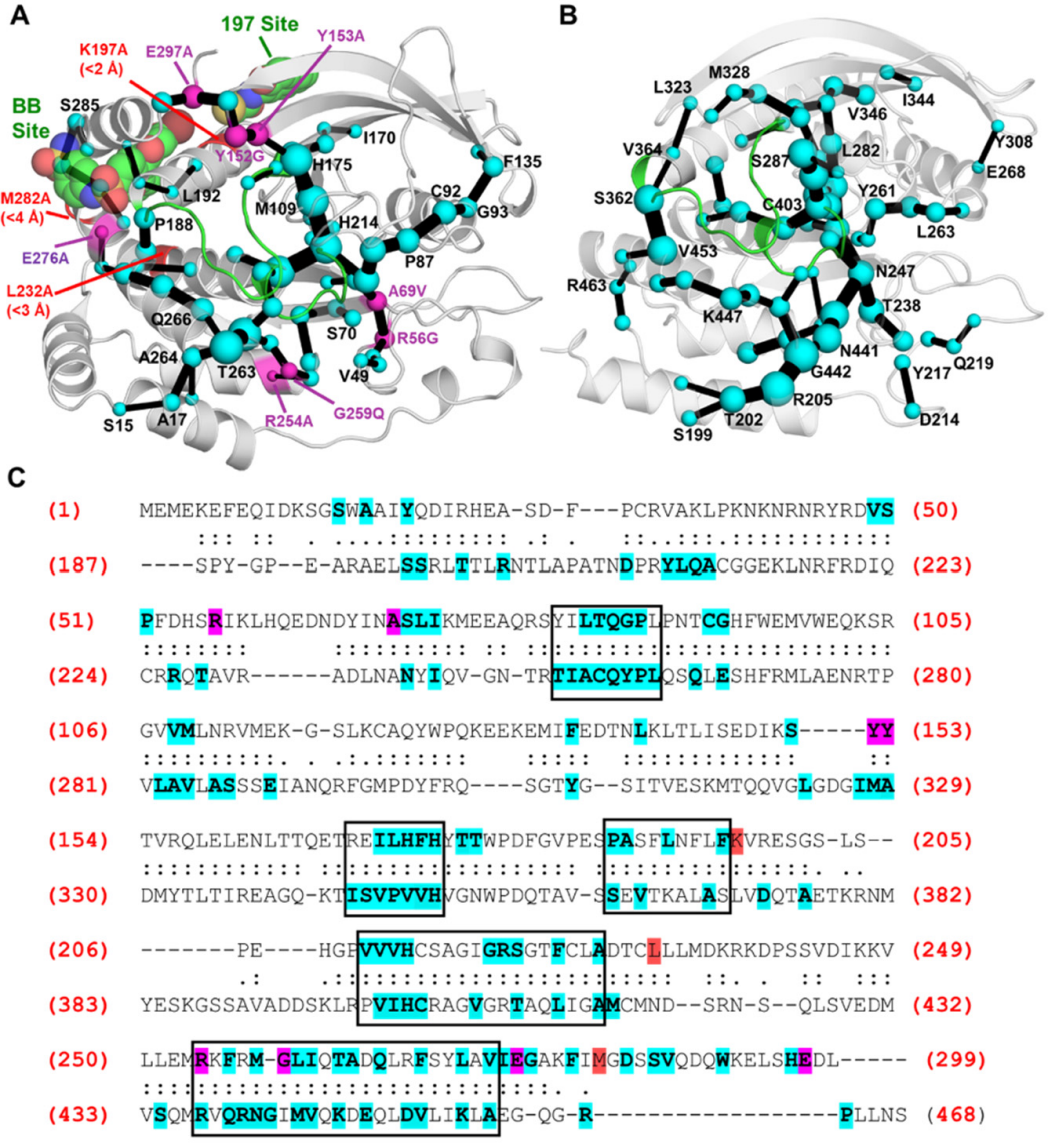
Identification of key residues and pathways utilized for allosteric communication in (A) PTP1B and (B) YopH, determined using the shortest path map (SPM) method.^52^ SPMs for both PTPs were calculated using our PT-MetaD-WTE simulations with *p*NPP-bound. The sizes of the spheres and edges are proportional to the number of pathways found through the residue (spheres) or between two residues (edges) (a larger size means more pathways and therefore more importance for allosteric communication). For PTP1B, non-WPD or P-loop mutations found on the SPM that are known to alter PTP1B activity by > |50%| are shown as purple spheres, with mutations not found on the SPM colored red. For mutations not found, the closest heavy atom distance to an SPM residue is indicated. The two known allosteric drug binding sites (BB and 197) are also depicted with a representative drug bound in each position using PDB IDs 1T49(75) and 6B95,^6^ respectively. (C) Structure-based sequence alignment of PTP1B and YopH, with all aligned residues marked with either a “:” or “.” (residues marked with a “:” have a C_α_–C_α_ distance within 5 Å of one another). All residues in PTP1B and YopH found on the SPM are highlighted in blue, with those known to affect enzyme activity (same criteria as in (A)) highlighted in purple if on the SPM or in red if not on the SPM. Boxes are used to highlight regions that have a high frequency of SPM residues in both PTPs. Structural alignment was performed using TM-align.^78^ PDB IDs 6B90(6) and 2I42(7) were used to describe PTP1B and YopH, respectively.

To complement our enhanced sampling simulations and in order to explore potential pathways of allosteric communication throughout both PTPs, we computed the shortest path maps^52^ (SPMs) for PTP1B and YopH in their *p*NPP-bound Michaelis complexes (Figure 6), as this is likely the most therapeutically relevant state for targeting by allosteric inhibitors.^9,75^ The SPM approach can identify key residues and pathways used for allosteric communication,^52^ both of which would be highly valuable for drug discovery efforts targeting allosteric inhibitors of PTPs.^4,76,77^

We then compared the SPM generated for PTP1B to the extensive literature available on its allosteric behavior (Figure 6A). In PTP1B, 68 residues (of 299 in total) are included in the SPM. Of the 11 non-WPD- or P-loop mutations that were shown to alter *k*_cat_ or *K*_m_ by > |50%| (as compared to the WT, see Table S9), eight were identified by the SPM as being important for allosteric communication. Further, of the three remaining mutations (K197, L232, and M282) that significantly alter *k*_cat_ or *K*_m_, all were within 4 Å (closest heavy atom distance) of a residue in the network. Finally, residues that form both of PTP1B’s known allosteric binding sites (Figure 6A) were identified in the SPM. These results (alongside further comparison to experimental data, see Section S2 of the Supporting Information) therefore provide us with confidence that our SPM can identify residues key to allosteric communication in PTP1B and therefore also in YopH.

In the case of YopH, 71 residues (of 282 in total) are included in the SPM. Given the limited information on YopH allostery and the above observations that our SPM results were able to identify distal mutation sites in PTP1B, including those conserved among other PTPs, we performed a structure-based sequence alignment of the two PTPs (Figure 6C). A comparison of the SPM residues identified in PTP1B and YopH reveals a reasonably high level of conservation between the two PTPs, with 35 of the 69 PTP1B SPM residues conserved in YopH. Notably, five structurally conserved regions in both PTPs show a high frequency of SPM residues, including those that make up the BB-site, suggesting that some of the allosteric pathways known in PTP1B are also present in YopH and that therefore YopH could possibly be targeted in a similar manner as has successfully been applied to PTP1B.

### Evaluation of the Stability of the Michaelis Complexes Formed During PTP Catalysis

**Figure 7:**
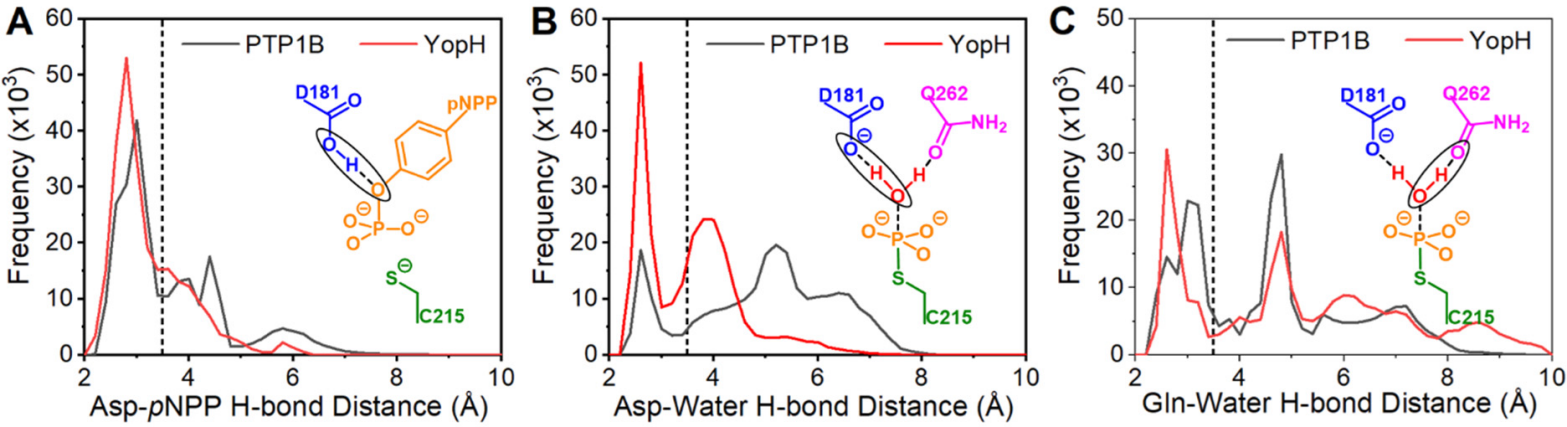
Histograms of hydrogen bonding (H-bond) distances for key interactions required for the formation of the Michaelis complexes for both the (A) cleavage and (B and C) hydrolysis steps of PTP catalysis. The chemical structure embedded into each panel represents the donor–acceptor distance measurement made (heavy atom distances). Histograms (bin size 0.2 Å) were obtained from 25 × 200 ns long MD simulations of each PTP, starting from the closed (catalytically competent) state. The dotted line at 3.5 Å on each graph indicates the approximate point at which an H-bond can no longer be considered formed. One sided harmonic restraints between the substrate and several P-loop residues were used to hold *p*NPP (panel A) in a catalytic competent pose throughout the simulations (see the Methodology section).

We sought to characterize the stability of the reactant complexes for both chemical steps of PTP catalysis. We therefore performed 25 × 200 ns MD simulations of both the *p*NPP-bound Michaelis complex and the phospho-enzyme intermediate, starting simulations from the closed (catalytically competent) state. Simulations with *p*NPP-bound were performed with restraints between *p*NPP and several residues on the P-loop (see Table S6) to ensure *p*NPP was consistently bound to the active site throughout these simulations. Histograms of the hydrogen bond (H-bond) distance between the aspartic acid on the WPD-loop and *p*NPP (Figure 7A) show some sampling of non-productive states for both PTP1B and YopH, which arise primarily from the side chain of the aspartic acid on the WPD-loop “swinging out” to form H-bonds with either the solvent and/or nearby residues (Figure S15). While simulations of the phospho-enzyme intermediate have no restraints in place, we observed a water molecule to be consistently coordinated to the phosphate group (Figure S15), likely because the phosphate group is solvent accessible, and following departure of the leaving group formed in the cleavage step from the active site, there is now sufficient space site for a water molecule to take its place (note that there is extensive experimental evidence that the substrate binds as a phosphodianion, see, e.g., refs (5, 20, 79, and 80)). We therefore evaluated the stability of the H-bonds formed by the catalytic aspartic acid and the coordinating glutamine on the Q-loop to the nucleophilic water molecule (Figure 7B,C). Analysis of Figure 7 suggests the active site of YopH is better configured to stabilize the reactant complexes formed for both steps of PTP catalysis. Note, however, that exchanges between productive and non-productive conformations occurred in both PTPs and for both reactant complexes on the nanosecond time scale, suggesting that the differences identified here may not contribute significantly to the experimentally observed rates for either step.

### Empirical Valence Bond Simulations

Both PTP1B and YopH catalyze the turnover of their substrates using the same two-step mechanism shown in Figure 1, involving the nucleophilic attack of an active site cysteine on the substrate to form a phospho-enzyme intermediate (cleavage) followed by nucleophilic attack of a water molecule to hydrolyze the phospho-enzyme intermediate (hydrolysis).^5^ YopH achieves this more efficiently than PTP1B, with turnover numbers that differ by ∼1 order of magnitude (*k*_cat_ values of ∼1300 s^–1^ for YopH^20^ compared to ∼40 s^–1^ for PTP1B^21^ at their pH optima). We note that the experimental rates for the first chemical step are similar, with a caveat that these were obtained at a pH of 6 and at 3.5 °C (as the first step is too fast to be detected at higher temperatures^81,82^), a more optimal pH for PTP1B catalysis than that of YopH. The main difference is observed in the rates for the subsequent, rate-limiting hydrolysis of the phospho-enzyme intermediate (Table 1). This difference is curious given the similarity in the active sites of the two enzymes, and therefore, in a final step, we used the EVB approach^30,31^ in order to model the cleavage and hydrolysis reactions catalyzed by PTP1B and YopH, respectively (Figure 1).

**Table 1:** Calculated Activation (Δ*G*^‡^) and Reaction Free Energies (Δ*G*_0_), Obtained Using the Empirical Valence Bond Approach, As Well As Relevant Corresponding Experimental Observables for Both Steps of Catalysis for Both PTPs

			experimental data			
	Δ*G*^‡^	Δ*G*_0_	*k* (s^–1^)	temperature (°C)	pH	Δ*G*^‡^_exp_
cleavage						
PTP1B	14.1 ± 0.1	4.5 ± 0.2	270^18^	3.5	5.4	13.1
YopH	11.5 ± 0.2	2.8 ± 0.3	343^83^	3.5	5.8	13.0
hydrolysis						
PTP1B	14.2 ± 0.2	–10.3 ± 0.3	28^18^	3.5	5.4	14.3
			48^9,14,32^	30	5	15.4
			24.4^23^	23	5.5	15.5
YopH	13.5 ± 0.2	–10.4 ± 0.3	1235^19^	30	5	13.5
			601^22^	30	5.5	13.9

a All calculated values are averages and standard errors of the mean over 30 individual EVB trajectories per system, with calculations performed at 30 °C, as described in the Methodology section. Both experimental and calculated activation and reaction free energies are presented in kcal mol^–1^. Shown here are also the corresponding kinetics (*k*, s^–1^) and activation free energies (Δ*G*^‡^_exp_) derived from the experimentally observed rates using the Eyring equation.

**Figure 8:**
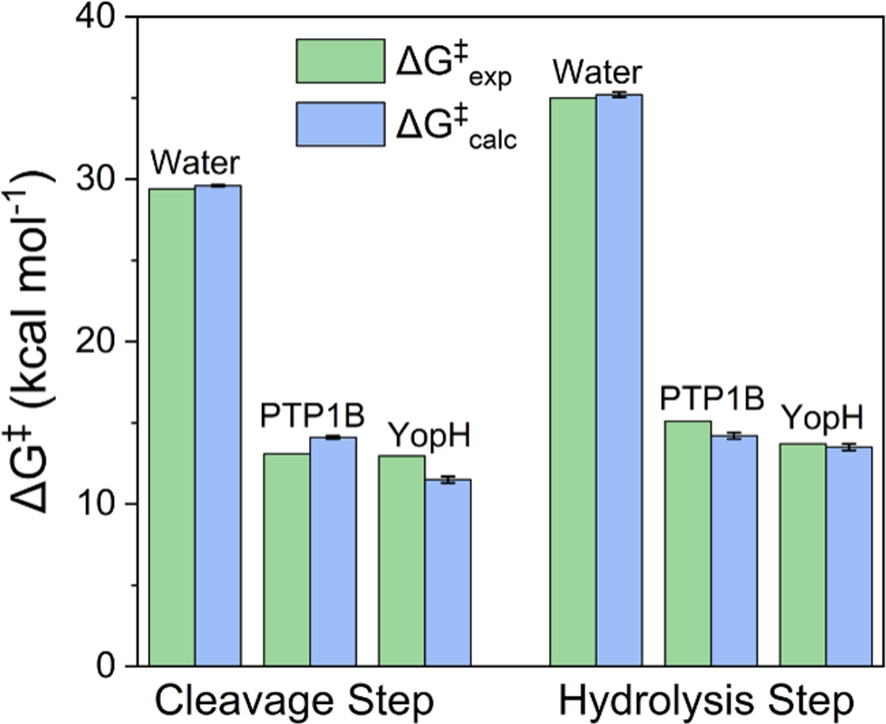
Comparison of the calculated (Δ*G*^‡^_calc_) and experimental (Δ*G*^‡^_exp_) activation free energies for the non-enzymatic and PTP1B- and YopH-catalyzed hydrolysis of *p*NPP. Shown here are separate data for each of the cleavage and hydrolysis steps shown in Figure 1. Data is presented in kcal mol^–1^ as the average values and standard error of the mean over 30 individual EVB trajectories obtained as described in the Supporting Information. The raw data for this figure is presented in Table 1 and Table S9.

The activation and reaction free energies obtained from our EVB simulations are provided in Table 1 and Figure 8. On the basis of these results, it can be seen that, for the cleavage step, we calculated an activation free energy that is 2.6 kcal mol^–1^ lower in YopH than in PTP1B, whereas for the hydrolysis step, the barrier is more similar and only 0.7 kcal mol^–1^ lower in YopH than in PTP1B. In both cases, we obtained a higher barrier for the hydrolysis step than the cleavage step, again in agreement with the experimental data. While our PTP1B calculations give generally good quantitative agreement with the experiment, our YopH calculations underestimate either the expected activation free energy compared to the experiment for the cleavage step or the difference between PTP1B and YopH for the hydrolysis step (see Table 1). However, a direct comparison to the experiment is not straightforward. That is, it has been shown experimentally that catalysis in these enzymes is correlated with WPD-loop motions, and therefore, it is quite possible that the two processes (chemistry and loop motion) are coupled. In such a case, one cannot reliably use the Eyring equation to obtain the experimental activation barrier for the chemical process on the enzyme, because the temperature effect on the rate of catalysis reflects other temperature-dependent events besides the phosphoryl transfer. The fact that we obtain relatively similar barriers for the hydrolysis step, for example, would be expected from the fact that these active sites are practically identical and superimposable; it in turn suggests that the difference in reaction rates is determined by a non-chemical event. We also note that the large catalytic effects (∼16 kcal mol^–1^ for cleavage and 20 kcal mol^–1^ for hydrolysis) observed for both enzymes and both chemical steps are well-reproduced by our simulations (Figure 8).

**Figure 9:**
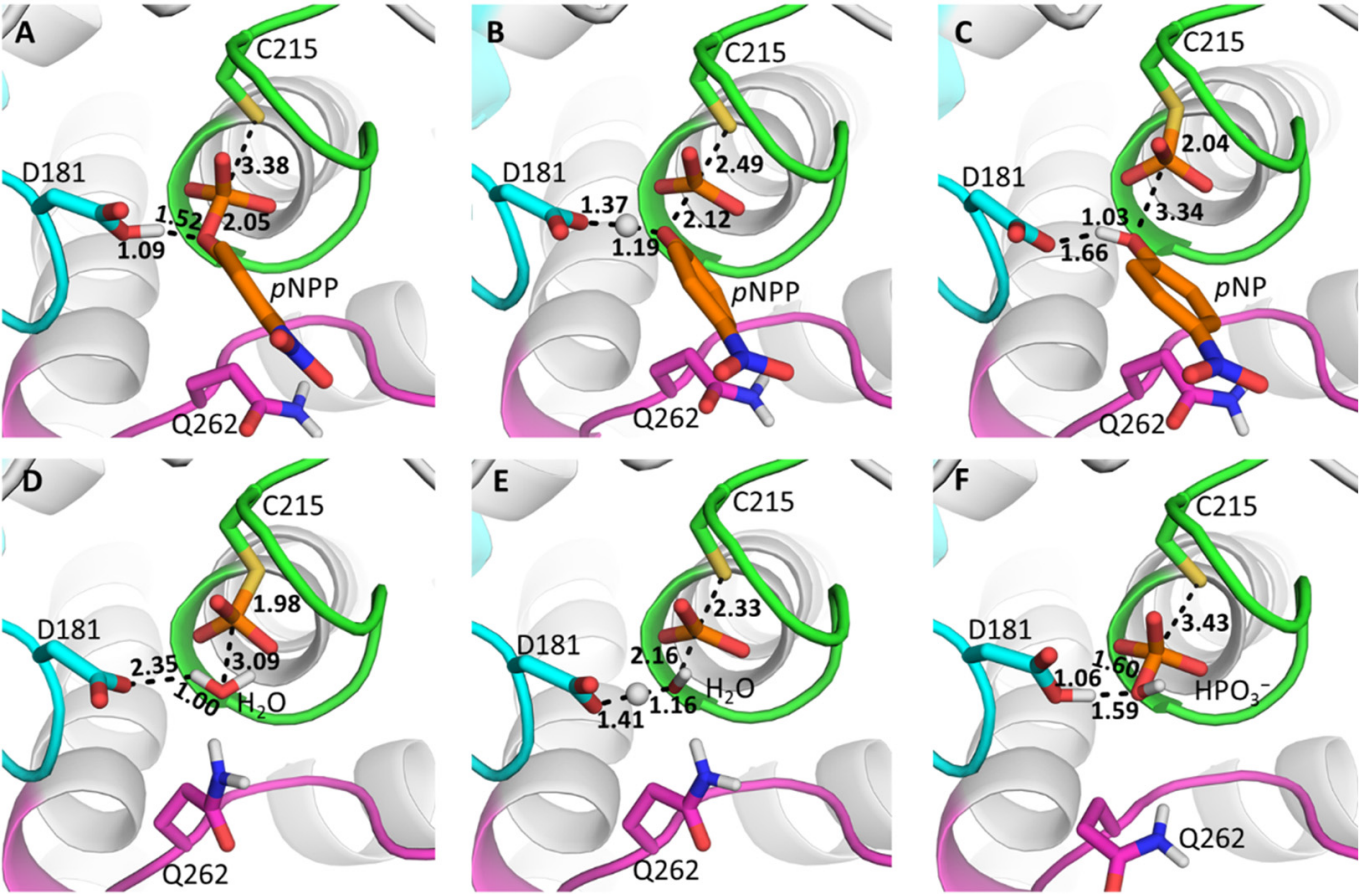
Representative structures of (A) the Michaelis complex, (B) the transition state for the cleavage step, (C and D) the phospho-enzyme intermediate, (E) the transition state for the hydrolysis step, and (F) the final product complex, for the PTP1B-catalyzed hydrolysis of *p*NPP (see Figure S16 for equivalent YopH results). The structures shown here are the centroids of the top ranked cluster obtained from RMSD clustering of 30 individual EVB trajectories of each stationary or saddle point, performed as described in the Supporting Information. Average reacting distances for each catalytic step are also shown.

Taking the limitations described above into account, we explored structural changes observed in our EVB simulations of the different reaction steps and systems (Figure 9). In terms of transition state geometries (Table S10), we observed very similar P–O distances to either the leaving group in the cleavage or nucleophile in the hydrolysis step between the non-enzymatic and enzymatic reactions (irrespective of enzyme). However, we observed a slight contraction in the S_Cys_–P distances with Pauling bond orders (see Section S2 of the Supporting Information for further details) of 0.42, 0.58, and 0.63 for the non-enzymatic reaction and the PTP1B- and YopH-catalyzed reactions, respectively, in the cleavage step, and 0.53, 0.76, and 0.80 in the hydrolysis step. For the P–O_*p*NPP_ distance in the cleavage step, the differences are much smaller, whereas for the hydrolysis step, the P–O_H_2_O_ bond orders follow a similar trend to the S_Cys_–P distances (from 0.50 to 0.40 and 0.38 for the three different reactions, respectively). From this analysis on the basis of the Pauling bond orders (see full data in Table S10), it is clear that (aside from the differences between the non-enzymatic- and enzyme-catalyzed reactions), the main differences between PTP1B and YopH are observed in the sulfur–phosphorus distances, for both reaction steps.

We also applied our EVB simulations to determine the per residue electrostatic contributions to TS stabilization (Figure S17 and Table S11). For both PTPs, many of the residues that provide TS stabilization for the cleavage reaction are destabilizing in the hydrolysis reaction, while residues that provide TS stabilization for the hydrolysis reaction are destabilizing in the cleavage reaction. As an example, K120 on PTP1B and R404 on YopH both coordinate the catalytic aspartic acid in their respective enzymes and each provide the largest contribution to TS stabilization for the cleavage step (Figure S17), where they help to stabilize the buildup of negative charge on the aspartic acid at the TS. In contrast, both K120 and R404 destabilize the hydrolysis step, in which the reverse process, protonation of the aspartate carboxylate, is unfavorable. This observation suggests that the active sites of PTPs have been subjected to competing evolutionary interests toward barrier reduction for both chemical steps. Further, we have previously observed symmetrical roles of residues between reaction steps for the enzyme β-phosphoglucomutase,^24^ which also undergoes a ping-pong reaction mechanism. This is likely a common feature among ping-pong reaction mechanisms, as the second reaction step is the reverse of the first.

It is also interesting to note that many of the key residues that provide substantial TS (de)stabilization for either or both of PTP1B’s reactions are located on the E-loop (residues: R112, E115, K116, and K120, see Figure S17). This would suggest that the conformational sampling of the E-loop is essential for productive catalysis and supports the proposition of Torgeson et al.,^11^ in which the conformational sampling of the WPD-loop and other active site loops including the E-loop control the observed *k*_cat_ (see discussion surrounding Figure 5).

Finally, in order to examine the solvent accessibility of reacting atoms in the active site, we monitored the average number of water molecules within 4 Å of the reacting atoms (Table S10). These values are similar between PTP1B and YopH for both reacting steps, but up to two additional water molecules (not including the nucleophilic water molecule) enter the active site at the transition state for the hydrolysis step compared to the cleavage step, which may partially account for the slightly higher barrier to the hydrolysis step (Table 1), although the higher barrier could also simply be due to the fact that the hydrolysis step (leaving group S-alkyl) is intrinsically more challenging to catalyze with a non-enzymatic barrier of ∼35 kcal mol^–1^ compared to ∼29.5 kcal mol^–1^ for the non-enzymatic equivalent of the cleavage of *p*NPP.^84−86^

## Overview and Conclusions

PTPs regulate a myriad of biological pathways, and as such, their catalytic rates will have been subjected to strict evolutionary pressures. Despite a shared catalytic mechanism and similar transition states for both chemical steps, PTP catalytic rates vary by orders of magnitude, and NMR has demonstrated a linkage between the rate of WPD-loop motion and catalysis in YopH and PTP1B.^9,14^ These differences likely apply throughout the classical PTP family. The results reported here provide an understanding of the basis for the differing conformational dynamics between YopH and PTP1B and insights into the origins of their respective catalytic activities. To the best of our knowledge, PTPs are the first enzyme family known that carries out the same reaction at highly different rates modulated by differences in their protein dynamics. An understanding of these differences also allows for consideration of their broader evolutionary implications.

Our structural analysis of PTP1B and YopH (Figure 1) identified a single principal competent (PC, i.e., vector) that describes the WPD-loop open-to-closed transition. Two additional hinge points flanked by proline residues in the WPD-loop of PTP1B (Figure 2) provide a structural rationale for its ∼50-fold slower loop motion compared to YopH.^9^ This was confirmed by HREX-MD simulations, which demonstrate that, while both PTPs sample a vast array of conformations, YopH samples more conformations and does so at a faster rate than PTP1B. Furthermore, HREX-MD simulations (Figure 3) identified YopH to be able to adopt a “hyper-open” WPD-loop conformation, previously only observed in the crystal structures of two WPD-loop swapped YopH-PTP1B chimeras,^14^ although these conformations are rare events that are infrequently sampled in wild-type YopH. This implies that the swapping of WPD-loop residues in the chimeras did not *cause* the hyper-open conformations observed in their crystal structures but merely stabilized this conformation, allowing it to be crystallized and also, presumably, to be populated to a higher degree, resulting in reduced catalytic activity.^14^ In contrast, wild-type PTP1B does not adopt this hyper-open conformation in our simulations and likely cannot do so, due to the role of P188 acting as a “helix-breaker”. The proposition that PTP1B cannot form this state is consistent with a P188A PTP variant showing two WPD-loop exchange processes, as compared to one for wild-type PTP1B and other point variants.^18^

The functional role (if any) of this hyper-open conformation and its prevalence among other PTPs is currently unknown, although we note that an atypical catalytically inactive hyper-open conformation has also been observed in three other PTPs from different subgroups (STEP, LYP, and GLEP1),^87^ further supporting that these hyper-open conformations are not artifactual but could have functional relevance for the superfamily as a whole. This suggests that, although the precise functional role of this hyper-open state is unknown and it is clearly a conformation incapable of facilitating chemistry, it nevertheless carries some functional significance for PTPs as a whole, as a means for the modulation of activity by these biologically important signaling enzymes. Here, it is possible that this unproductive state is preferentially (de)stabilized by the binding of regulators or changes in the cellular environment, allowing a means for the control of catalysis.

Our MD simulations of the reactant state showed local fluctuations of reacting side chain atoms on the nanosecond time scale (Figure 7), demonstrating that the large-scale closure of the WPD-loop is not the only prerequisite for efficient catalysis. Additionally, modest differences were found in the computed activation barriers between PTP1B and YopH for the initial cleavage step and very similar computed activation barriers for the rate-determining hydrolysis step (Table 1). While not surprising given the similar active sites and transition states, the experimentally measured rates are very different.^9^ This further strengthens the notion that protein motions contribute to the differences in *k*_cat_ in the PTP family.^14^

Our PT-MetaD-WTE simulations (Figure 4) were able to reproduce the experimentally observed population shift toward the closed WPD-loop state in the presence of substrate and in the phospho-enzyme intermediate state. This population shift is induced not only through direct interactions between the ligand/thiol-phosphate group and the WPD-loop acid but also through stabilizing a salt bridge between the side chains of a highly conserved P-loop arginine and E-loop glutamic acid (Figure 5). These simulations also identified the WPD-loop and E-loop motions to be correlated with one another for both PTPs. However, the properties of PTP1B’s and YopH’s E-loops are notably different from one another, with PTP1B possessing a highly flexible E-loop that can sample many conformations, while YopH possesses a highly rigid preorganized E-loop (Figure 5). Additionally, the analysis of per-residue contributions to TS (de)stabilization for PTP1B identified a key role for many E-loop residues (Figure S17), suggesting that the correct conformational sampling of this loop is essential for catalysis. In contrast, no E-loop residue was observed to play a significant role the TS (de)stabilization for YopH. These insights are particularly noteworthy given that a recent NMR study has a proposed *k*_cat_ for PTP1B that reflects cooperative fluctuations between the WPD- and E-loops.^11^

Our EVB simulations reveal subtle changes in transition state geometries and solvent exposure of the active site between the different systems, which can likely account for the differences in calculated activation free energies between the different reaction steps and enzymes shown in Table 1. However, more significantly, our data indirectly suggests that the observed differences in rate between the two enzymes are linked to changes in WPD-loop dynamics, which has been already suggested on the basis of experimental work,^9^ and E-loop dynamics. Specifically, our EVB simulations predict very similar activation free energies for the rate-limiting hydrolysis of the phospho-enzyme intermediate (Table 1). This is unsurprising, considering the near identical active sites and shared reaction mechanisms of the two enzymes. This therefore strongly suggests that the rate of a non-chemical step, in this case the conformational transitions of the WPD- and E-loops, is driving the differences in turnover number.

In contrast to PTP1B, our observations show that YopH has a highly rigid E-loop with low importance for TS (de)stabilization, which may mean that YopH’s *k*_cat_ is controlled primarily by fluctuations in only the WPD-loop. This could be probed experimentally using the same approaches as those applied to PTP1B.^11^ Here, simulations can also be used to identify amino acid substitution(s) that can shift the population between the WPD-loop open and closed conformations toward a permanently closed conformation (an example of such a population shift toward a more populated closed conformation was observed in a recent study performed by our group^88^). Examining the effect of such a population shift on the turnover numbers would allow for probing why some PTPs, such as those studied in the present work, use a general acid located on a mobile loop for catalysis. On the contrary, modeling of the direct coupling between the conformational change and the chemical step of catalysis would be extremely computationally challenging and out of the scope of the present work; however, clear differences are indicated in the dynamical behavior of the WPD-loops of PTP1B and YopH on the basis of our enhanced sampling and empirical valence bond simulations, which further support this observation.

Finally, we utilized correlation-based methods to identify the key residues and pathways for allosteric communication in both PTP1B and YopH (Figure 6). An agreement of these predictions with a substantial body of experimental allosteric data for PTP1B lends confidence to the predictions of analogous potential allosteric regions in YopH, which has been significantly less well-characterized in this regard. These data suggest that the allosteric regulation of YopH may be possible, although to our knowledge it has not been probed experimentally, thus suggesting an avenue for further experimental work. In addition, a comparison of SPMs^52^ produced for both PTP1B and YopH also showed a high degree of conservation of the residues playing significant roles in allosteric communication. Given the relatively low sequence similarities between PTP1B and YopH (20.6% sequence identity), this raises the likelihood that these conserved regions are also present in other PTPs, which would be consistent with a recent study that identified evolutionarily conserved mechanisms of allosteric communication among several PTPs.^26^

In summary, while PTP1B and YopH are chemically and mechanistically indistinguishable in their chemical steps of catalysis, there are clear differences in their WPD-loop and E-loop dynamics, an insight that can only be obtained in the detail presented here using simulation approaches. While the active site electrostatic environment is clearly important for the TS stabilization of both reaction steps that would otherwise be extremely slow, without the correct conformational dynamics, the enzyme would not be able to reach a catalytically productive Michaelis complex for chemistry to occur. If altered loop dynamics are primarily responsible for regulating PTP catalysis, then this raises biological questions as to how and why nature “chose” this approach. It is possible that this allows PTPs to respond to changes in their local environment, such as changes in temperature, pH, viscosity, or crowding. It also provides a means for allosteric regulation by small molecules or proteins that affect WPD-loop motions. Physiologically crucial PTPs like PTP1B must function at rates that meet the physiological requirements of the organism, where the fastest rate is not necessarily optimal. It is likely not a coincidence that YopH has evolved to become the fastest PTP yet characterized, given its role in facilitating *Yersinia* infection where “running wild” in the invaded host is beneficial. Future work could focus on how different (cellularly relevant) environmental conditions can alter the loop dynamics of PTPs. Furthermore, given the recently renewed interest in the allosteric inhibition of PTPs,^89,90^ understanding the similarities and dissimilarities in the allosteric regulation of human PTPs may prove valuable in the design of selective allosteric inhibitors.

## Supporting Information Available

Discussions of supplementary methods and results and Cartesian coordinates, figures of projections of crystal structures, relative mobility of each residue in each PC projection, projection of PCs 1–3 onto a representative structure of PTP1B, C_α_ RMSDs of the WPD-loop to the closed X-ray crystal structures of PTP1B and YopH, PT-MetaD-WTE simulations, distance between the phosphorous atom of *p*NPP and the sulfur atom of the catalytic cysteine over the course of MD simulations, distance histograms, representative structures of catalytically competent and catalytically incompetent substrate binding modes, time evolution of the free energy, diffusive dynamics, graphical depiction of the collective variables, percentage variance described by each principal component, PCA of the WPD loops of the X-ray structures, RMSFs of all C_α_ atoms, mobility plots, aligned structures, heat maps, measurement of sequence conservation among PTPs, dynamic cross correlation matrixes, electrostatic contributions of selected amino acids to calculated activation free energies, valence bond states, key reacting distances, and atom type labeling, and tables of all X-ray crystal structures obtained from the PDB, X-ray crystal structures used for simulations, non-standard protonation states and histidine tautomerization states used, GAFF2 force field and ff14SB force field parameters used, restraints used to stabilize the *p*NPP substrate, collective variables used for our PT-MetaD-WTE simulations of PTP1B and YopH, non-WPD-loop or P-loop single point mutations available from the literature, calculated distances at the Michaelis complexes, transition states, and product states, electrostatic contributions of individual amino acids, list of ionized residues and histidine protonation patterns used, key reacting distances for the non-enzymatic model reaction, impact of the WPD-loop conformation on the predicted p*K*_a_ values of all ionizable residues in PTP1B and YopH, empirical valence bond parameters used, list of the atom types and van der Waals parameters, bond types and corresponding parameters used, angle types and their corresponding parameters used, torsion angle types and the corresponding parameters used, improper torsion types and the corresponding parameters, atom types in the different valence and covalent bond states used, partial charges for the different valence bond states used, angle types used to describe the angles in the reacting part of the system, and improper torsion angle types used (PDF)
