# Correction to “Loop Dynamics and Enzyme Catalysis in Protein Tyrosine Phosphatases”

**DOI:** 10.1021/jacs.2c04624

**Published:** 2022-05-24

**Authors:** Rory M. Crean, Michal Biler, Marina Corbella, Ana R. Calixto, Marc W. van der Kamp, Alvan C. Hengge, Shina C. L. Kamerlin

**Affiliations:** † Science for Life Laboratory, Department of Chemistry − BMC, Uppsala University, Box 576, S-751 23 Uppsala, Sweden; § School of Biochemistry, University of Bristol, Biomedical Sciences Building, University Walk, Bristol BS8 1TD, United Kingdom; ‡ Department of Chemistry and Biochemistry, Utah State University, Logan, Utah 84322-0300, United States

We recently discovered that the partial charges presented in Table S23 of our original study^1^ are incorrect. We have therefore redone the associated empirical valence bond (EVB) simulations,^2^ yielding results that differ numerically but are qualitatively similar, with one exception described below. After addressing these issues, the discussion and conclusions of our original study remain valid.

Upon further examination of the literature, we observed that the experimental rate for the alkaline hydrolysis of the *p*-nitrophenyl phosphate (*p*NPP) dianion likely corresponds to attack at the aromatic ring with C–O cleavage, and not P–O cleavage, due to the resistance of phosphate ester dianions to attack by hydroxide ion.^3,4^ By extension, the reaction with a thiolate nucleophile (analogous to that of these protein tyrosine phosphatases (PTPs)) is even less likely to occur in solution due to the larger size of S compared to O. As this makes it impossible to know what the barrier to a hypothetical uncatalyzed reaction proceeding through P–O cleavage would be, it renders a comparison to the non-enzymatic reaction energetically uninformative.

Since the non-enzymatic reaction is not a good reference state, we have used instead the PTP1B-catalyzed reaction (fit to experimental kinetics) as our reference state, and we use this as a baseline for comparison with YopH, as in our recent studies of other enzymes.^5,6^ Here, we have used the p*K*_a_ difference in solution between the nucleophile and leaving group at each reacting state to calibrate the reaction free energies, yielding values of −1.4 and −10.2 kcal mol^–1^ to describe the cleavage and hydrolysis steps, respectively. We have also made some minor parameter and simulation protocol updates based on best practices developed moving forward with this project. These modifications change the absolute free energy barriers presented in Table 1 of the original submission, but the relative values remain similar. We provide here updated figures, tables, and EVB parameters, and a data package that includes all relevant parameters, input files, and simulation snapshots, as well as a brief description of any other changes to parameters and protocol, as additional Supporting Information.

Finally, please note the addition of two new authors to the manuscript, Drs. Ana Rita Calixto and Marina Corbella, who discovered and rectified the technical issues outlined above.

## 1 Updated Analysis

### Impact of Performing the EVB Hydrolysis Step Simulations as One Continuous Process or Two Discrete Steps

The data presented in Table 1 shows the results of modeling the cleavage and hydrolysis steps using different starting structures, as outlined in the Methodology section of the original paper.^1^ Crystal structures suggest that after the cleavage step, a repositioning occurs of the side chain of a conserved glutamine on the Q-loop (Q262 and Q446 in PTP1B and YopH, respectively). For comparison, we also ran continuous EVB simulations of the hydrolysis step from the endpoint of the EVB simulations of the cleavage reaction, without the prior repositioning of the Q side chain. Here, we have repeated those simulations for comparison to the data presented in Table 1, and obtained marginally higher activation free energies of 14.6 ± 0.2 and 14.7 ± 0.2 kcal mol^–1^ for the hydrolysis steps catalyzed by PTP1B and YopH, respectively, when using the EVB endpoints from the cleavage step.

**Table 1:** Calculated Activation (Δ*G*^⧧^) and Reaction Free Energies (Δ*G*_0_), Obtained Using the Empirical Valence Bond Approach, As Well As Relevant Corresponding Experimental Observables for Both Steps of Catalysis for Both PTPs^a^

			experimental data			
	Δ*G*^⧧^	Δ*G*_0_	*k* (s^–1^)^b^	temp (°C)	pH	Δ*G*^⧧^_exp_
Cleavage						
PTP1B	13.0 ± 0.1	–1.4 ± 0.3	270^18^	3.5	5.4	13.1
YopH	11.7 ± 0.2	–3.7 ± 0.3	343^83^	3.5	5.8	13.0

Hydrolysis						
PTP1B	14.3 ± 0.2	–1.4 ± 0.4	28^18^	3.5	5.4	14.3
			48^9,14,32^	30	5	15.4
			24.4^23^	23	5.5	15.5
YopH	14.1 ± 0.2	–2.9 ± 0.3	1235^19^	30	5	13.5
			601^22^	30	5.5	13.9

a All calculated values are averages and standard errors of the mean over 30 individual EVB trajectories per system, with calculations performed at 30 °C, as described in the Methodology section of the original paper.^1^ Both experimental and calculated activation and reaction free energies are presented in kcal mol^–1^. Shown here are also the corresponding kinetics (*k*, s^–1^) and activation free energies (Δ*G*^⧧^_exp_) derived from the experimentally observed rates using the Eyring equation. Note that for both steps, the calculated activation free energy for PTP1B has been fit to the experimental value at 3.5 °C for consistency, as this is the temperature for which a rate constant is available for the cleavage step.

b Citations in this section refer to the References in the original paper.^1^

### Analysis of Reacting Distances from Our EVB Simulations

We present updated Pauling bond orders for each of the PTP1B/YopH transition states, calculated from the data presented in Table S10, using the relationship *r* = *r*_e_ – 0.6 ln(*n*). Specifically, for the cleavage step we obtained bond orders of 0.60 and 0.62 for the S_Cys_–P distance for PTP1B and YopH, respectively, and both PTPs had a bond order of 0.43 for the P–O_*p*NPP_ bond. For the hydrolysis step, the S_Cys_–P bond order was 0.77 for PTP1B and 0.76 for YopH, while the P–O_H20_ bond order was 0.43 for both PTPs. Thus, the transition states are very similar between the two enzymes.

## 2 Updated Main Text and Supplementary Figures

Figures 8, 9, S16, S17, and S21 have been updated; Figures 8 and 9 are shown here, and Figures S16, S17, and S21 are presented in the corrected Supporting Information.

**Figure 8:**
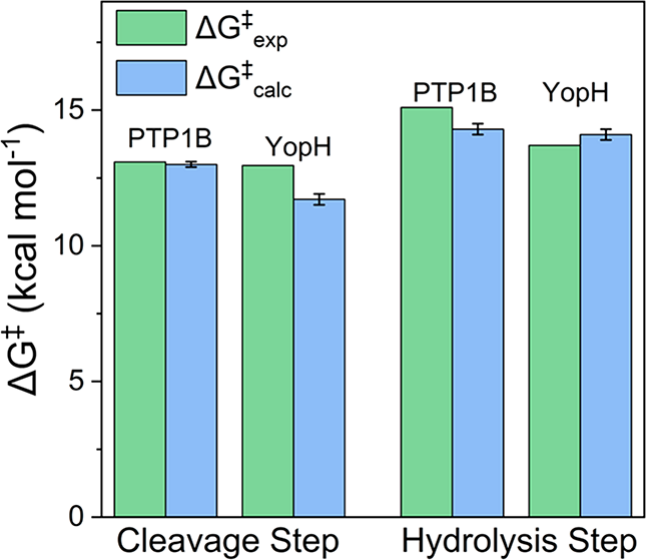
Comparison of the calculated (Δ*G*^⧧^_calc_) and experimental (Δ*G*^⧧^_exp_) activation free energies for the PTP1B- and YopH-catalyzed hydrolysis of *p*NPP. Shown here are separate data for each of the cleavage and hydrolysis steps shown in Figure 1. Data is presented in kcal mol^–1^ as average values and standard error of the mean over 30 individual EVB trajectories obtained as described in the Supporting Information. The raw data for this figure is presented in Tables 1 and S9.

**Figure 9:**
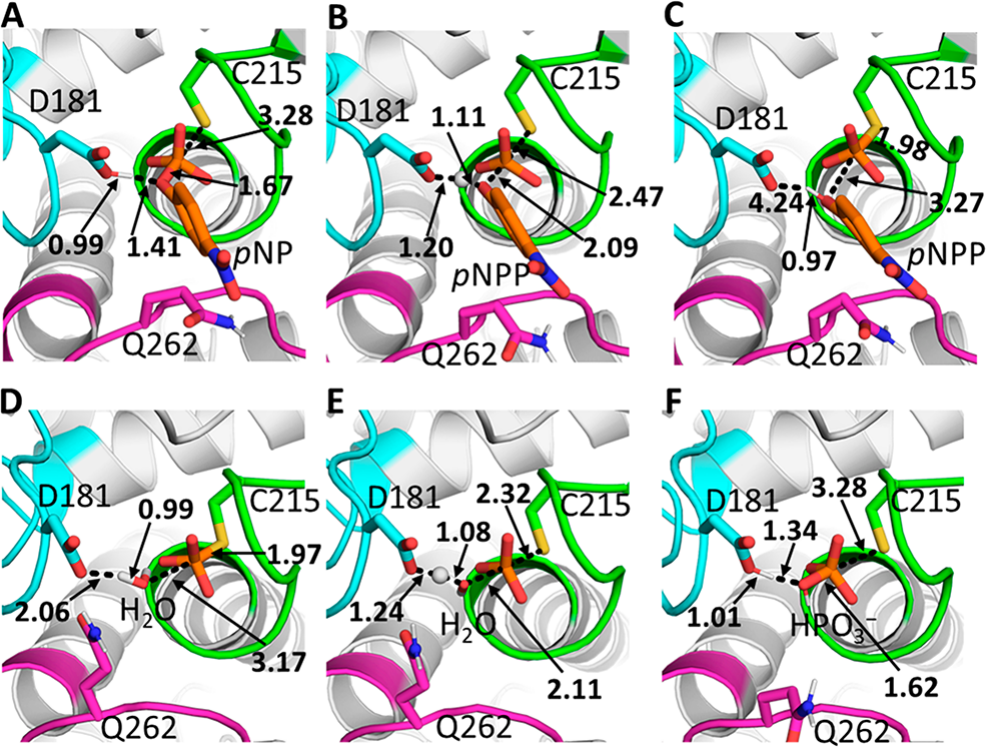
Representative structures of (A) the Michaelis complex, (B) the transition state for the cleavage step, (C and D) the phospho-enzyme intermediate, (E) the transition state for the hydrolysis step, and (F) the final product complex, for the PTP1B-catalyzed hydrolysis of *p*NPP (see Figure S16 for equivalent YopH results). The structures shown here are the centroids of the top-ranked cluster obtained from RMSD clustering of 30 individual EVB trajectories of each stationary or saddle point, performed as described in the Supporting Information. Average reacting distances for each catalytic step are also shown.

## 3 Updated Main Text and Supplementary Tables

Tables 1, S10, S11, and S12 have been updated; Table 1 is shown here, and Tables S10–S12 are presented in the corrected Supporting Information.

## 4 Updated Empirical Valence Bond Parameters

Tables S16–S19 and S22–S25 have been updated and are presented in the corrected Supporting Information.

In Table S16, the values of *H*_*ij*_ and α were calibrated to reproduce experimental activation free energies for the PTP1B-catalyzed cleavage and hydrolysis reactions, and reaction free energies of −1.4 and −10.2 kcal mol^–1^ for the cleavage and hydrolysis reactions, respectively. Tables S17–S19 and S22–S25 highlight modified parameters.

## Supporting Information Available

Discussions of supplementary methods and results and Cartesian coordinates, figures of projections of crystal structures, relative mobility of each residue in each PC projection, projection of PCs 1–3 onto a representative structure of PTP1B, C_α_ RMSDs of the WPD-loop to the closed X-ray crystal structures of PTP1B and YopH, PT-MetaD-WTE simulations, distance between the phosphorous atom of *p*NPP and the sulfur atom of the catalytic cysteine over the course of MD simulations, distance histograms, representative structures of catalytically competent and catalytically incompetent substrate binding modes, time evolution of the free energy, diffusive dynamics, graphical depiction of the collective variables, percentage variance described by each principal component, PCA of the WPD loops of the X-ray structures, RMSFs of all C_α_ atoms, mobility plots, aligned structures, heat maps, measurement of sequence conservation among PTPs, dynamic cross correlation matrixes, electrostatic contributions of selected amino acids to calculated activation free energies, valence bond states, key reacting distances, and atom type labeling, and tables of all X-ray crystal structures obtained from the PDB, X-ray crystal structures used for simulations, non-standard protonation states and histidine tautomerization states used, GAFF2 force field and ff14SB force field parameters used, restraints used to stabilize the *p*NPP substrate, collective variables used for our PT-MetaD-WTE simulations of PTP1B and YopH, non-WPD-loop or P-loop single point mutations available from the literature, calculated distances at the Michaelis complexes, transition states, and product states, electrostatic contributions of individual amino acids, list of ionized residues and histidine protonation patterns used, key reacting distances for the non-enzymatic model reaction, impact of the WPD-loop conformation on the predicted p*K*_a_ values of all ionizable residues in PTP1B and YopH, empirical valence bond parameters used, list of the atom types and van der Waals parameters, bond types and corresponding parameters used, angle types and their corresponding parameters used, torsion angle types and the corresponding parameters used, improper torsion types and the corresponding parameters, atom types in the different valence and covalent bond states used, partial charges for the different valence bond states used, angle types used to describe the angles in the reacting part of the system, and improper torsion angle types used (corrected, with revisions shown in blue type) (PDF)
Data package, including clustered snapshots, inputs, libs, starting PDBs, and topologies (ZIP)
