# Supplementary material for: Loop Dynamics and Enzyme Catalysis in Protein Tyrosine Phosphatases
Source: J Am Chem Soc. 2021 Mar 4;143(10):3830–45. doi: 10.1021/jacs.0c11806 (PMC8031367; doi:10.1021/jacs.0c11806)
Supplement: Supplementary file 1 — ja0c11806_si_001.pdf [file ja0c11806_si_001.pdf]

Supporting Information for:

Loop Dynamics and Enzyme Catalysis in Protein Tyrosine Phosphatases

Rory M. Crean,[†] Michal Biler,[†] Marc W. van der Kamp,[§] Alvan C. Hengge*,[‡] and Shina C. L. Kamerlin*,[†]

[†] Science for Life Laboratory, Department of Chemistry – BMC, Uppsala University, BMC, Box 576, S-751 23 Uppsala, Sweden.

[§] School of Biochemistry, University of Bristol, Biomedical Sciences Building, University Walk, Bristol BS8 1TD, United Kingdom.

[‡] Department of Chemistry and Biochemistry, Utah State University, Logan, Utah 84322-0300, United States.

*****Corresponding Authors

E-mail: alvan.hengge@usu.edu and lynn.kamerlin@kemi.uu.se

Table of Contents

S1. Supplementary Methodology S3

Crystal Structure Analysis S3

Root Mean Square Fitting Selections for X-ray Structure and Simulation Analysis S4

System Preparation for Conventional and Enhanced Sampling Molecular Dynamics Simulations S4

Ligand Parameterization for Conventional and Enhanced Sampling Molecular Dynamics Simulations S6

Conventional Molecular Dynamics Simulations S7

Equilibration Procedure for Conventional Molecular Dynamics Simulations S8

Equilibration Procedure for Enhanced Sampling Molecular Dynamics Simulations S9

Hamiltonian Replica Exchange Molecular Dynamics Simulations S10

Parallel Tempering Metadynamics Simulations S11

Analysis of Conventional and Enhanced Sampling Molecular Dynamics Simulations S13

System Preparation for the Empirical Valence Bond Simulations S16

Parameterization and Calibration of the Empirical Valence Bond Simulations S17

System Equilibration and Empirical Valence Bond Simulations S20

EVB Simulation Analysis S21

S2. Supplementary Results S23

Further Analysis of the Crystal Structure PC Projections S23

Hamiltonian Replica Exchange Sampling and Convergence S25

Changes in pK_a along the WPD-loop Opening/Closing Pathway S26

Identification of Residues Directly Correlated to WPD-Loop Motion S27

Further Description of the SPM Generated for PTP1B S29

Impact of Substrate Restraints on the Michaelis Complexes Stability. S29

Impact of Performing the EVB Simulations as One Continuous Process or Two Discrete Steps S32

Analysis of Reacting Distances From our EVB Simulations S33

S3. Supplementary Figures S34

S4. Supplementary Tables S58

S5. Empirical Valence Bond Parameters S77

S6. Supplementary References S92

S7. Cartesian Coordinates of QM Optimized Stationary Points S112

S1. Supplementary Methodology

Crystal Structure Analysis

Crystal structures of PTP1B and YopH were obtained from the RCSB Protein Data Bank (PDB).[1] Structures with missing residues within the residue range E2-I281 for PTP1B and S187-K456 for YopH were not taken forward for further analysis, to allow Principal Component Analysis (PCA) to be performed over these regions. PCA produces orthogonal eigenvectors (principal components, PCs) that describe the variance of the different structures, with the PCs produced ranked by the amount of variance they describe in the given dataset. PCA was performed in Cartesian coordinate space on the C_α carbons of the WPD-loops of all structures studied.

A total of 231 and 16 structures of PTP1B and YopH being included in PCA, respectively, with 3 additional YopH-PTP1B chimera structures also included. A complete list of all PDB structures used for the analysis of each enzyme is provided in **Table S1**. PCA was then performed on these structures using the CPPTRAJ[2] module that is part of the AmberTools18[3] suite of programs, by first reformatting all structures into Amber-readable format (through a combination of shell scripts and the pdb4amber program within AmberTools18[3]), and subsequently performing PCA on the C_α carbon atoms of the residues selected for the analysis. PCA was performed by first generating an average structure based on all the X-ray crystal structures used, and then performing root mean square (RMS) fitting of all structures to this average structure for the subsequent PCA. Several different RMS fitting procedures were used depending on the analysis being performed (for example, whether the PCA was being performed on both PTP1B and YopH simultaneously or independently), and the specific residues chosen for all analyses are provided in the section **Root Mean Square Fitting Selections for X-ray Structure and Simulation Analysis**.

Root Mean Square Fitting Selections for X-ray Structure and Simulation Analysis

As described in the main text, several different root mean square deviation (RMSD) fitting procedures were used for our analyses, and the specific RMS fitting procedure was chosen based on the context in which the analysis was being performed. Residues selected for RMS fitting were stable/rigid (and with defined secondary structure) such that they would provide a good RMS fit throughout the simulations (unlike *i.e.*, a flexible loop region which would sample many different regions of conformational space). For individual PCA of PTP1B, RMS fitting was performed on the C_α atoms of residues: 15-26, 33-41, 69-84, 91-102, 106-109, 133-150, 153-162, 166-176, 188-201, 212-214, 220-237, 246-256, and 264-281. For individual PCA on YopH and the YopH-PTP1B chimeras,[4] RMS fitting was performed on the C_α atoms of residues 191-208, 246-251, 254-259, 264-277, 281-284, 288-296, 306-308, 311-324, 327-337, 344-351, 362-386, 389-392, 400-402, 408-420 and 429-440. For combined PCA on the WPD-loops of all enzymes, RMS fitting was performed on 55 residues which are structurally conserved between the YopH and PTP1B scaffolds, and relatively rigid. The quality of the chosen residue selection was validated by outputting an RMS fitted trajectory of both the PTP1B and YopH simulations together, to allow for visual evaluation of the quality of the fit. For PTP1B, the selected residues were: 68-72, 170-175, 212-231, 247-256. For YopH, the selected residues were: 245-249, 256-260, 345-350, 351-365, 400-419 and 430-439.

System Preparation for Conventional and Enhanced Sampling Molecular Dynamics Simulations

A total of six crystal structures[5–9] were used in this study to generate the starting points for simulations of the unliganded forms, Michaelis complexes with *p*-nitrophenyl phosphate (*p*NPP) and covalent phospho-enzyme intermediates for PTP1B and YopH, with their WPD-loops in both their open and closed conformations (see **Table S2** for a full list of structures used, and the

modifications required for preparation of each system). In brief, simulations at the *p*NPP-bound Michaelis complexes were prepared by modifying structures with a peptide inhibitor bound, while simulations of the phospho-enzyme intermediates were prepared by modifying structures with a vanadate ion bound to the active site (by replacing the vanadate ion with inorganic phosphate). Simulations of the Michaelis complexes were performed with the catalytic aspartic acid (D181 in PTP1B and D356 in YopH, respectively) protonated and the cysteine nucleophile deprotonated, whilst simulations of both the unliganded enzyme and phospho-enzyme intermediate were performed with the catalytic aspartic acid on the WPD-loop deprotonated (protonation states selected to be in line with the chemical mechanism depicted in **Figure 1**). Care was taken to ensure the protonation and tautomerization of all other residues were kept consistent for all simulations, based on a combination of empirical screening using PROPKA[10] v3.1 and visual inspection. Based on a predicted pK_a of 8.27 for both the WPD-loop open and closed conformations (using both conformations available in PDB ID: 6B90[8], E102 of PTP1B was protonated in all our simulations. Finally, tautomerization states and any required Asn or Gln side chain "flips" were predicted using the MolProbity[11] server, with the results visually inspected to ensure consistency across all simulations (tautomerization states used for all simulations are provided in **Table S3**). For all conventional and enhanced sampling MD simulations, structures were solvated in an octahedral water box, with the box size selected such that no protein atom was within 10 Å of the box boundary, and Na^+ or Cl^- counterions were added as required to ensure overall charge neutrality of the system. System solvation and other simulation preparations for our EVB simulations are described separately in the section **System Preparation for the Empirical Valence Bond Simulations**.

Ligand Parameterization for Conventional and Enhanced Sampling Molecular Dynamics Simulations

Partial charges for the ligand, *p*NPP (modelled in its dianion form), were calculated using the standard restrained electrostatic potential (RESP) protocol, using Antechamber.[12] The electrostatic potential of *p*NPP was determined *in vacuo* at the HF/6-31G(d) level of theory, using Gaussian 16 Rev. A.03,[13] after having performed geometry optimization at the same level of theory. All other force field terms for *p*NPP were then described using the general Amber force field 2 (GAFF2),[14] with relevant parameters provided in **Table S4**. In the case of the phosphorylated cysteine residue, geometry optimization of this residue was performed at the MP2/6-311G(d,p) level of theory, using capping acetyl (ACE) and N-methylamide (NME) capping groups, and implicit solvent, using the conductor-like polarizable continuum model (CPCM).[15] As with *p*NPP, charges were determined *in vacuo* at the HF/6-31G(d) level of theory, using Gaussian 16 Rev. A.03.[13] Further, the charges of the main chain and capping group atoms were kept constant (in order to match the charges of deprotonated cysteine from the Amber ff14SB force field[16]), and only the side chain charges were allowed to change. All other parameters were taken directly from the ff14SB force field, where possible, and any remaining missing terms were obtained using GAFF2.[14] The parameters used to describe the covalent phospho-enzyme intermediate are provided in **Table S5**. These parameters were then used for all conventional and enhanced sampling molecular dynamics simulations. In the case of the EVB simulations, these simulations were performed using the OPLS-AA force field[17,18] for consistency with previous related studies,[19–22] and therefore separate EVB parameters were derived as described in the section **Parameterization and Calibration of the Empirical Valence Bond Simulations**.

Conventional Molecular Dynamics Simulations

All conventional MD simulations were performed using the GPU-accelerated version of Amber16,[3] with the protein and water molecules described using the ff14SB[16] force field and the TIP3P[23] water model, respectively. Simulations of all 12 different systems of interest here (unliganded, *p*NPP-bound Michaelis complexes and phospho-enzyme intermediates, starting from both open and closed conformations of the WPD-loops of both PTP1B and YopH) were performed for 25 x 200 ns each, in the NPT (300 K, 1 atm) ensemble. In order to prepare for production quality MD simulations, we used a minimization, heating and equilibration procedure, that is described in the section **Equilibration Procedure for Conventional Molecular Dynamics Simulations**. All production quality MD simulations were performed using a 2 fs time step, with the SHAKE algorithm[24] used to constrain all bonds containing hydrogen atoms. The temperature and pressure during the simulations were regulated using Langevin temperature control[25] (collision frequency of 1 ps^{-1}), and a Berendsen barostat[26] (pressure relaxation time of 1 ps), respectively. All simulations used an 8 Å direct space non-bonded cutoff, with long range electrostatics being evaluated using the particle mesh Ewald algorithm.[27] Four distance and one angle restraint(s) were applied during simulations of the Michaelis complex to maintain the *p*NPP substrate in a catalytically competent configuration throughout the MD simulations (see **Table S6** for a full list of restraints applied). Equivalent restraints were put in place for both PTP1B and YopH simulations, and we note that no restraints were placed between the substrate and the WPD-loop to ensure full conformational freedom of this loop. These restraints were only applied to the Michaelis complex simulations to prevent dissociation of *p*NPP from the active site upon WPD-loop opening, and were used for both the conventional MD simulations and the parallel tempering metadynamics simulations.

Equilibration Procedure for Conventional Molecular Dynamics Simulations

A common equilibration procedure was used for all conventional molecular dynamics simulations in this work, which used the Amber16 simulation package.[3] All simulation steps used the SHAKE algorithm[24] to restrain all bonds to hydrogen atoms, and simulations of the *p*-nitrophenyl phosphate (*p*NPP)-bound Michaelis complexes of PTP1B and YopH included the additional restraints described in **Table S6**, which were applied to prevent the substrate from dissociating from the active site. In the first step, all hydrogen atoms and solvent molecules (including any counterions) were energy minimized using first 500 steps of steepest descent minimization, followed by 500 steps of conjugate gradient minimization. In order to keep the protein and substrate fixed in place during the minimization, 10 kcal mol^{-1} $Å^{-2}$ positional restraints were applied to all heavy (non-hydrogen) protein and ligand atoms. These positional restraints were retained as the system was heated from 50 to 300 K in an NVT ensemble over the course of 200 ps of simulation time using a 1 fs time step with Langevin temperature control[25] (collision frequency of 1 ps^{-1}). For the subsequent steps, the restraints were reduced from 10 to 5 kcal mol^{-1} $Å^{-2}$ and applied to only the C_α atoms of all protein residues. Following this, another energy minimization and heating process was performed, again using 500 steps of steepest descent minimization followed by 500 steps of conjugate gradient minimization. The restraints on the C_α atoms were retained as the system was heated from 25 K to 300 K over the course of 500 ps in an NVT ensemble using the same conditions as described above. Simulations were then performed in the NPT ensemble (300 K, 1 atm) using Langevin temperature control[25] (collision frequency of 1 ps^{-1}) and a Berendsen barostat[26] (1 ps pressure relaxation time). NPT simulations were performed using a 2 fs simulation time step. The 5 kcal mol^{-1} $Å^{-2}$ positional restraints described above were slowly released in 1 kcal mol^{-1} $Å^{-2}$ increments every 10 ps of 50 ps of simulation time

Finally, a 1 ns long unrestrained MD simulation was performed for further equilibration, using the same NPT conditions as described above. After this, simulations were then considered ready for production simulations. All replicas were assigned different random velocity vectors for both heating steps, to generate 25 independent starting structures for production MD simulations of each state simulated.

Equilibration Procedure for Enhanced Sampling Molecular Dynamics Simulations

This procedure was used for simulations that used GROMACS with PLUMED, which corresponds to both the Hamiltonian replica exchange molecular dynamics (HREX-MD)[28] and parallel tempering metadynamics simulations performed in the well-tempered ensemble (PT-MetaD-WTE) protocols. For simulations with *p*NPP-bound to the active site, all dynamics steps included the substrate restraints described in **Table S6**. Furthermore, during all equilibration steps, 2.39 kcal mol^{-1} $Å^{-2}$ positional restraints were applied to all heavy atoms.

First, the systems were subjected to 5000 steps of steepest descent and then conjugate gradient energy minimization. Next, systems were heated from 25 to 300 K over the course of 500 ps in an NVT ensemble using Langevin temperature control[25] (collision frequency of 1 ps^{-1}) and a time step of 1 fs. Following this, the systems were equilibrated in the NPT ensemble (300 K, 1 atm) using Langevin temperature control[25] and a Parrinello−Rahman barostat[29] (pressure relaxation time of 1 ps) to regulate temperature and pressure, respectively. NPT equilibration was performed with a time step of 2 fs. Both NVT and NPT equilibration simulations used the P-LINCS[30] algorithm to restrain all bonds to hydrogen atoms. Furthermore, all simulation steps used a 10 Å non-bonded interaction cut-off was used to evaluate long range electrostatic interactions, using the Particle Mesh Ewald (PME) algorithm.[27]

Hamiltonian Replica Exchange Molecular Dynamics Simulations

Hamiltonian replica exchange molecular dynamics (HREX-MD),[28] which has been described in detail elsewhere (see ref. [31]), is an enhanced sampling simulation technique that provides an unbiased approach to increase the rate of sampling of the free energy landscape for a region of interest. In the present work, HREX-MD simulations were performed on the unliganded enzyme structures of PTP1B and YopH, using the systems prepared as described in the section **System Preparation for Conventional and Enhanced Sampling Molecular Dynamics Simulations**. All HREX-MD simulations were performed using the Amber ff99SB-ILDN[32] force field and TIP3P[23] water model as implemented into GROMACS 2018.4,[33] interfaced with PLUMED v2.5[34] (the AMBER ff99SB-ILDN was chosen over the AMBER ff14SB[16] for HREX-MD simulations as only force fields embedded into the GROMACS software can be used for this simulation methodology). Following a standard minimization, heating and equilibration procedure (described in detail in the **Supplementary Methodology**), two production quality HREX-MD simulations of 500 ns per replica (over 8 replicas, see below) were performed for PTP1B and YopH each, with one simulation starting from structures with the WPD-loop in its closed conformation, and the other starting from a loop-open conformation to maximize the potential sampling of the conformational space. All HREX-MD simulations were performed in the NPT ensemble, using a 2 fs time step and the P-LINCS algorithm[30] to restrain all bonds to hydrogen atoms. A 10 Å non-bonded interaction cut-off was used to evaluate long range electrostatic interactions, using the Particle Mesh Ewald (PME) algorithm.[27] The simulations were performed using Langevin temperature control[25] (collision frequency of 1 ps^{-1}) and with pressure regulated using a Parrinello-Rahman barostat[29] (pressure relaxation time of 1 ps).

When setting up the HREX-MD simulations, a generous definition of the WPD-loop was used to define the residues included in the "hot region" of the simulations (i.e., residues 175-191 in the case of PTP1B, and 349-365 in the case of YopH). The Hamiltonian of the hot region was then modified by scaling the partial charges by $\sqrt{\lambda}$, the Lennard Jones parameters by λ, and the dihedral terms by λ (with the exception of the first and last residues of the "hot region", which were scaled by $\sqrt{\lambda}$). A total of 8 replicas, with λ values exponentially scaled between 1.0-0.6 (λ values of 1.0, 0.930, 0.864, 0.803, 0.747, 0.694, 0.645, 0.6 were used), resulting in an effective temperature range of 300-500 K. Exchanges between replicas were attempted every 1 ps, achieving an average exchange rate of 40% for PTP1B and 38% for YopH. Subsequent analysis was performed solely on the neutral replicas (λ=1).

Parallel Tempering Metadynamics Simulations

Parallel tempering metadynamics performed in the well-tempered ensemble (PT-MetaD-WTE)[35,36] is a form of metadynamics simulation well suited to describe complex reaction coordinates such as protein conformational changes. PT-MetaD-WTE (which combines temperature based replica exchange with metadynamics simulations) is particularly useful method for sampling complex reactions coordinates such as the protein conformational change simulated here,[37–39] as any slow degrees of freedom not well described by the chosen metadynamics CVs will still have their sampling enhanced through the use of temperature based replica exchange.

During conventional metadynamics[40] simulations, two or three collective variables (CVs) are normally chosen to describe the process under investigation, with each CV being biased by a history dependent Gaussian-type bias. In PT-MetaD-WTE,[41] multiple metadynamics simulations are propagated in parallel with each replica assigned a different temperature (in our case temperatures of 300, 312.62, 325.78, 339.47, 353.75, 368.63, 384.13 and 400.29 K were used) and

exchanges between adjacent replicas are attempted periodically. By performing simulations in the well-tempered ensemble (WTE), we are able to enhance the energy fluctuations of each replica whilst still keeping the average close to the canonical value. This in practice means one can use a much smaller number of replicas over a given temperature range and still achieve a reasonable exchange rate as compared to standard PT-MetaD.[35,36] PT-MetaD-WTE simulations were performed with GROMACS 2018.4,[33] interfaced with PLUMED v2.5,[34] using the Amber ff14SB[16] force field and the TIP3P[23] water model (systems were setup with "tleap" in AmberTools18[3] and then converted to a GROMACS compatible format using the program "parmed", also in AmberTools18).

Following initial structure preparation (see the section **System Preparation for Conventional and Enhanced Sampling Molecular Dynamics Simulations**), PT-MetaD-WTE simulations were performed on all of the unliganded, *p*NPP-bound Michaelis complex, and phospho-enzyme intermediate states of both PTP1B and YopH (*i.e.*, 6 systems in total). After the equilibration of each replica to its target temperature, PT-MetaD-WTE simulations were performed in two stages. First, a 10 ns long PT-MetaD simulation was performed with a bias potential placed on the potential energy of the system. In this step the bias factor, Gaussian height and Gaussian deposition rates was set to 60, 0.6 kcal mol^{-1} and 1 ps for both PTP1B and YopH, respectively. The Gaussian widths were set to 100 kcal mol^{-1} and 110 kcal mol^{-1} for PTP1B and YopH simulations, respectively. In the second step, the bias on the potential energy was retained (in order to run simulations in the WTE) but no additional Gaussians were deposited onto this CV. Instead, three CVs were chosen to describe WPD-loop motion (see below) for the production PT-MetaD-WTE simulations, which were run for between 700-800 ns per replica. Simulations were stopped once they had been observed to be converged, which was assessed by the analysis of the time evolution

of the free energy profiles (**Figure S1** and **Figure S2**), alongside the clear observation of diffusive dynamics along each CV (see **Figure S3** and **Figure S4**).

Exchanges between replicas were attempted every 1 ps, and all six PT-MetaD-WTE simulations had exchange rates between 21-29%. The 3 CVs chosen (**Figure S5** and **Table S7** and **Table S8**) were used to enable sampling of the free energy landscape of WPD-loop motion. CV1 was the interloop distance-RMSD (DRMSD) of the C_α atoms of the WPD- and P-loops, with the closed crystal structures used as the reference structure. CV2 and CV3 describe the motions in the central and C-terminal portions of the WPD-loop respectively through a center of mass distance measurement between the WPD-loop residues to atoms on the P- or Q-loops (which are highly rigid). Gaussians with an initial height of 0.2 kcal mol^{-1} were deposited every 2 ps, with the Gaussian height gradually reduced over the course of the simulation by using a bias factor of 12. Gaussians widths were set to 0.1, 0.3 and 0.3 Å, respectively, and wall potentials were used to prevent the sampling of non-relevant states (walls were placed to prevent sampling of states beyond those seen in the HREX-MD simulations). All analysis was performed on the replica simulated at 300 K and simulations were reweighted and projected onto unbiased CVs using the approach described by Tiwary and Parrinello.[42] The minimum free energy pathway (MFEP) analysis was performed using the Minimum Free Energy Path Surface Analysis (MEPSA) approach.[43]

Analysis of Conventional and Enhanced Sampling Molecular Dynamics Simulations

Unless stated otherwise, all analysis of all conventional and enhanced sampling molecular dynamics simulations was performed using the CPPTRAJ[2] module that is part of the AmberTools18[3] suite of programs. All hydrogen bonds formed between the WPD-loop and the remainder of the enzyme were identified using a donor-acceptor distance cut-off of 3.5 Å, and a

donor-hydrogen-acceptor angle of 180±45°. Only hydrogen bonds with an occupancy of >1% of the cluster simulation time were included in the subsequent analysis. PCA on the trajectories was performed in the same manner as described in the structural analysis section (RMS fit to a stable region of the enzyme, generate average structure, RMS fit to the average structure and then perform PCA on the C_α carbons of this trajectory). The raw populations of PC1 and PC2 from each simulation were converted to a 2D histogram (using 900 bins in total, *i.e.*, 30 x 30 bins on each axis) and scaled using a natural log scale. Natural log scaling was chosen as it directly relates populations to Gibbs free energy. Dynamic cross correlation matrices (DCCMs) and average inter-residue distance matrices of the PT-MetaD-WTE trajectories were computed using CPPTRAJ[2] for the C_α atom of every residue. Prior to the DCCM calculation, the same RMS fitting procedure as used for PCA calculations was applied.

In the SPM approach,[44] dynamic cross correlation maps (DCCMs) and average inter-residue distance matrices are computed and used to calculate the shortest (communication) pathways between all nodes (here, a residue is a node) in the network. Nodes and edges (pathways between nodes) that are often used for communication between residues can then be identified, and these residues can therefore be considered as being important for regulating the global conformational dynamics of the enzyme. Shortest Path Maps (SPM)[44] were determined using the DCCM and average inter-residue distance matrices for the simulations of PTP1B and YopH with *p*NPP-bound to the active site, using the available python script.[44]

The closest water molecule to the phosphorus atom on the phosphate group in the phospho-enzyme intermediate of each enzyme was determined using the "closest" command within CPPTRAJ,[2] in all cases selecting the closest water molecule to the phosphorus atom in each snapshot (using the oxygen atom of the water molecule to determine the distance between the

water molecule and the phosphorus atom). In a similar manner, as the aspartic acid oxygens in **Figure 7B** are equivalent, the closest oxygen atom was identified at each snapshot and used to generate the histogram. Distances and hydrogen bonds were also measured using CPPTRAJ.[2] The secondary structure content of WPD-loop residues was determined using the DSSP algorithm available within CPPTRAJ.[2]

Whilst our CVs allow for efficient sampling of the WPD-loop's accessible conformational space, they do not clearly distinguish between the open and closed WPD-loop states (2 out of the 3 of the CVs are combinations of main and side chain atom distances, See **Figure S5** for further explanation). We therefore reweighted our PT-MetaD-WTE simulations, projecting the underlying free energy landscape onto the inter-distance RMSD between the WPD- and P-loops (*i.e.*, CV1) and the fraction of native contacts[45] (described below) between the WPD- and P-loops (**Figure 4**). This allowed us to clearly distinguish between the closed and open state(s) of the WPD-loop alongside constructing a minimum free energy pathway (MFEP) between both states.

The native contacts parameter (Q)[45] provides a measure of the distance from a given configuration to a reference configuration (in this case the closed state of the WPD-loop), through the combination of multiple distance measurements. Q values are assigned between 1 and 0, with a value of 1 indicating the reference state (100% retention of native contacts) and decreasing values indicating a move away from the reference state. Q was calculated as described in Eq. 1:

$$Q(X) = \frac{1}{S} \sum_{(i,j)\in S} \frac{1}{1 + \exp\left[\beta\left(r_{ij}(X) - \lambda r_{ij}^{0}\right)\right]} \qquad \textbf{(1)}$$

Where X is the conformation along the reaction coordinate, X is the conformation along the reaction coordinate, $r_{ij}(X)$ is the distance between atoms i and j for a given conformation X, r_{ij}^{0} is the reference distance between atoms i and j and S is the number of all possible atom pairs. Both

the β (regulates the softness of the switching function) and λ (scales how rapidly Q changes with increasing distance) values are adjustable parameters. In our case the β value was kept as 5 $Å^{-1}$ as is recommended,[45] whilst the λ values was set to 1.2 for both enzymes (value chosen as it spreads the snapshots out well between 1 and 0). The C_α distances between equivalent residues on the WPD-loop and P-loop were used for both enzymes. For PTP1B, WPD-loop residues 176-188 and P-loop residues 214-220 were used, for YopH, WPD-loop residues 351-363 and P-loop residues 402-408 were used. The PTP1B and YopH closed state WPD-loop reference structures used for the native contacts definitions were PDBs: 6B90[8] and 2I42[7], respectively.

Empirical estimations of the pK_a values of all titratable residues as a function of WPD-loop closure were performed using PROPKA v3.1.[10] For each native contact value, a total of 100 frames were taken from our PT-MetaD-WTE simulations of PTP1B and YopH (with *p*NPP-bound) and subjected to the PROPKA calculation (and then averaged).

System Preparation for the Empirical Valence Bond Simulations

Empirical valence bond (EVB)[46] simulations were performed of both the cleavage and hydrolytic steps in the PTP1B- and YopH-catalyzed hydrolysis of *p*NPP. The structures used to describe each chemical step are the same as those used for our conventional and enhanced sampling simulations, and are described in **Table S2**. Specifically, we used PDB IDs: 3I7Z[9] and 1QZ0[5] to describe the cleavage step and 3I80[9] and 2I42[7] to describe the hydrolysis step in the reactions catalyzed by PTP1B and YopH, respectively, as these structures were solved in complex with either appropriate transition state analogues to describe the Michaelis complex, or vanadate ion as a proxy for the phospho-enzyme intermediate.

All systems were solvated in a 23 Å radius droplet of TIP3P[23] water molecules, centered on the backbone oxygen atoms of the arginine of the P-loop (R221 and R409 using PTP1B and YopH

numbering, respectively), and described using the Surface Constrained All Atom Solvent (SCAAS) model,[47] as in our previous work.[20,21,48–50] In this model, all residues within the inner 85% of the sphere are fully mobile during the simulations, all residues within the outer 15% of the sphere are restrained to their original crystallographic positions using 10 kcal mol^{-1} $Å^{-2}$ harmonic positional restraints, and all residues outside the water droplet are constrained to their crystallographic coordinates using 200 kcal mol^{-1} $Å^{-2}$ harmonic positional restraints. Residues outside the water droplet and in the restrained region of the droplet were simulated in their uncharged forms, in order to avoid introducing system instabilities associated with the presence of charged residues outside the explicit simulation sphere. All residues within the mobile region of the droplet (the inner 85% of the sphere) were simulated in the ionization states predicted from pK_a estimates performed as described in the section **Analysis of Conventional and Enhanced Sampling Molecular Dynamics Simulations**. A complete list of the assigned protonation states of all ionizable residues in our EVB calculations, as well as the histidine tautomerization states assigned, are provided in **Table S12**.

Parameterization and Calibration of the Empirical Valence Bond Simulations

A starting point for EVB simulations is selecting a well-defined reference state for calibration of the EVB off-diagonal coupling element, H_{ij}, and the gas-phase shift, α (for a detailed description of what these EVB parameters mean, see *e.g.*, refs. [46,51]). This reference state can be for instance the non-enzymatic reaction in vacuum or in solution, or the wild-type enzyme as a baseline for examining the effect of different enzyme variants. The EVB parameters are then calibrated to reproduce activation ($\Delta G^{\ddagger}$) and reaction (ΔG^0) free energies for the reference state based on either experimental data or predictions made from high-level calculations. Once the reference state has been parameterized, calculations for all other systems are performed using the same EVB

parameters, unchanged between systems for a discussion of the transferability of the EVB parameters, see *e.g.* refs. 52,53).

In the present work, we used the nucleophilic attack of ethanethiolate on *p*NPP and the hydrolysis of the resulting thiolphosphate (with the *p*-NP leaving group removed) in the presence of propionic acid as an acid/base catalyst as model systems to describe the corresponding cleavage and hydrolysis steps shown in **Figure 1** (for a description of the valence bond states, see **Figure S18** and **Figure S19**). We calibrated our EVB parameters to reproduce activation free energies of 29.4 and 35 kcal mol^{-1} for the cleavage and hydrolysis steps, respectively, based on extrapolation from experimental data presented in refs. [54–56], as well computational modeling of the hydrolysis of methyl phosphate.[57] In addition, we used the pK_a difference in solution between the nucleophile and leaving group at each reacting state to calibrate the reaction free energies, yielding values of −1.4 and −10.2 kcal mol^{-1} to describe the cleavage and hydrolysis steps, respectively. This calibration resulted in the EVB parameters provided in **Section S5**, which were in turn used to describe all systems studied herein.

Following from this, we also performed quantum chemical calculations using density functional theory (DFT) to characterize the transition state for the nucleophilic attack of ethanethiolate on *p*NPP in the presence of propionic acid to protonate the departing leaving group (as a model for the catalytic aspartic acid side chain present in PTP1B and YopH). Due to the availability of experimental data against which to calibrate our EVB off-diagonal coupling element, H_{ij}, and the gas-phase shift, α (**Section S5**), our focus here was on obtaining a meaningful QM geometry for the model reaction against which to parameterize our EVB bonding parameters, with the obtained the S_{Cys}-P and P-O_{pNPP} distances to the nucleophile and leaving group then used to parameterize bond formation and cleavage in our corresponding EVB simulations.

We note that we focused here on only the cleavage step, as we then used the same geometric EVB parameters in reverse to describe the corresponding hydrolysis step. We performed our calculations using the M06-2X functional,[58] and the 6-31+G(d,p) basis set, using the Gaussian09 (Rev. E01) software package.[59] Solvation was described using a mixed explicit/implicit solvent model as in our previous work[22,57,60] with implicit solvation taken into account using the Solvation Model based on Density (SMD) model,[61] and with the inclusion of six explicit water molecules in order to ensure all hydrogen bond donor/acceptor sites were saturated. We first obtained an optimized transition state for this reaction, which was validated by vibrational frequency analysis and by following the intrinsic reaction coordinate (IRC)[62,63] in both reactant and product directions to verify that it is the correct transition state for the process of interest (*i.e.*, group transfer coupled with proton transfer to the leaving group from propionic acid). The corresponding distances and geometries of key stationary points are shown in **Table S13** and **Figure S20** and the Cartesian coordinates for the optimized stationary points are provided in **Section S7**.

Partial charges of the corresponding moieties were calculated as described in the section **Ligand Parameterization for Conventional and Enhanced Sampling Molecular Dynamics Simulations**. All other OPLS-AA-compatible force field parameters were obtained using the "ffld_server", as implemented in Schrödinger's Macromodel suite,[64] and then converted to *Q*6 readable format using *Q*tools v0.5.10 (DOI: 10.5281/zenodo.842003). The Morse bond parameters, as well as the non-bonded parameters for the exponential repulsion function placed on the reactive bond being formed/cleaved during the reaction were parameterized to reproduce the distances of the bonds at the transition state obtained in our QM geometry optimization of the model reaction (**Table S13**), and the same parameters were then used for all systems studied in this work. All EVB parameters necessary to reproduce our work can be found in the **Section S5**.

System Equilibration and Empirical Valence Bond Simulations

EVB simulations in this work were performed using the *Q*6 simulation package,[65] using the OPLS-AA force field as implemented into *Q*6.[18] A 10 Å cut-off was set for all non-bonded interactions, except those involved in the chemical reactions, for which the cut-off was set to 99 Å (effectively no cut-off). Long-range electrostatic interactions were described using the local reaction field (LRF) approach,[66] and the temperature was controlled by the Berendsen thermostat.[26] All systems underwent a sequence of short equilibration and heating simulations over a total of 213 ps of simulation time, in order to gradually remove possible steric clashes and bad contacts in the system prior to equilibration at 300 K. Once the system reached 300 K, all restraints on the mobile region of the protein were removed, and only weak (0.5 kcal mol^{-1} $Å^{-2}$) harmonic positional restraints were retained on the reacting moieties. Finally, we performed a further 30 ns of equilibration to fully equilibrate the system (backbone RMSDs over the course of the equilibration procedure are provided in **Figure S21**).

In line with previous work,[21,48,49] for computational efficiency, all equilibrations were performed at the approximate EVB transition state ($\lambda = 0.5$), as this allows for EVB trajectories in the reactant and product directions to be propagated simultaneously thus reducing human time, while also allowing for faster convergence of the simulations. We performed 30 individual equilibrations of each system being simulated, yielding a total cumulative simulation time of 0.9 μs per system and per catalytic step. The final structure from each equilibration run was then used as the starting point for the subsequent EVB production simulations, with one production EVB trajectory generated from the endpoint of each equilibration run.

Production EVB runs were performed using the valence bond states depicted in **Figure S18** and **Figure S19**, as described in the main text. The weak harmonic positional restraints of 0.5 kcal

mol^{-1} $Å^{-2}$ placed on the reactive atoms during the equilibration were retained for the production EVB simulations as well. Moreover, at the second catalytic step (the hydrolysis reaction), the P-O_{nuc} distance at the phospho-enzyme intermediate was restrained using a one sided harmonic wall potential, which was inactive at distances between 0 and 4 Å, and which applied a force constant of 10 kcal mol^{-1} $Å^{-2}$ for distances beyond 4 Å to keep the reacting fragments within 4 Å of each other. This wall potential was only active in the intermediate state, as the P-O_{nuc} bond was fully formed by the final product state. This restraint was introduced to prevent water exchange between the nucleophile and other solvent molecules as we observed in unrestrained simulations, simply due to the fact that while these water molecules are otherwise chemically equivalent, the nature of a VB-based model requires pre-defining the reacting atoms and therefore it is important to be able to track the nucleophilic water molecule during the reaction. In order to be consistent with the hydrolysis reaction, a one-sided wall potential with the same parameters as described above was used to restrain the reacting S_{Cys}-P distance to the nucleophile during the cleavage reaction (which was again only active in the Michaelis complex).

Finally, we note here that for comparison, we also modelled the cleavage step using the end-point of our EVB simulation (which does not include the motion of Q446 into the active site in the case of YopH). In doing so, we obtain an identical activation free energy in the case of PTP1B (14.2 ± 0.4 kcal mol^{-1}), whereas in YopH we obtain a higher activation free energy (17.6 ± 0.3 kcal mol^{-1}) when Q446 is not in a catalytically competent conformation, in agreement with structural data.

EVB Simulation Analysis

Analyses of root mean square fluctuation (RMSF), and root mean square deviations (RMSD) of all backbone atoms during our simulations were performed using AmberTools16.[3] All other

analyses were performed using *Q*6,[65] *Q*tools v0.5.10 (DOI: 10.5281/zenodo.842003) and VMD 1.9.3.[67] RMSD clustering was performed on all catalytic residues/substrates and water molecules directly involved in the chemical reaction, using the algorithm of Daura *et al*.[68] as implemented in GROMACS v.2018.1,[33] with an RMSD cut-off 0.05 Å. The centroids of the top ranked cluster of each state were used to produce **Figure 9** (PTP1B) and **Figure S16** (YopH).

S2. Supplementary Results

In this section we provide additional results from those presented in the main text for certain sections. Figure labelling for this section is independent of the rest of the document for readability purposes and labelled with the format: "**Figure SRX**", where **SR** stands for **Supplementary Results** and **X** is the figure number.

Further Analysis of the Crystal Structure PC Projections

Alongside the separate principal component analysis (PCA) performed on the WPD-loops of PTP1B and YopH crystal structures described in the main text, we also performed PCA on the WPD-loop conformations of all PTP structures together (**Figure SR1**). Our focus was on distinguishing between different closed and open conformations of the loop, including a non-productive "hyper-open" conformation of the WPD-loop observed in YopH-PTP1B chimeras[4] in which some WPD-loop residues of YopH were replaced with those of PTP1B. For this a total of 250 structures of PTP1B, YopH and YopH-PTP1B chimeras (231, 16 and 3 structures each respectively) were used, as described in the **Supplementary Methodology**.

We projected the coordinates of each crystal structure onto the first three PCs (**Figure SR1**), which describe 96.2% of the total variance observed in the data, with all remaining PCs providing a relatively small contribution (**Figure S6**). Analysis of the PC projections and each PCs' corresponding mobility plot (**Figure SR1C-F**) demonstrates that the first principal component, PC1, describes the open-to-closed transition of the WPD-loop. This is further validated by the well-defined partition of the open and closed crystal structures of PTP1B and YopH along PC1 (we note here that PDB ID: 1QXK[69] represents a semi-closed conformation of PTP1B, induced by the presence of an active site inhibitor), (**Figure SR1A-B**). PC2, on the other hand, appears to

largely describe differences in the structures of the residues that anchor to the C-terminal portion of the WPD-loop (the α4- and α3-helices of PTP1B and YopH, respectively), thus partitioning the PTP1B, YopH and YopH-PTP1B chimeras into three clearly defined groups. Unlike PC2, PC3 describes variance primarily in the central portion of the WPD-loop, but, like PC2, does appear to describe structural differences between PTP1B, YopH and YopH-PTP1B WPD-loops, and not the open-to-closed transition. In summary, our PCA calculations on available structural data suggest that the open-to-closed transition of both the WPD-loops of PTP1B and YopH can largely be described by a single PC. This structural differences between PC1 (loop opening/closure) for PTP1B and YopH were addressed by PCA performed on the structures of PTP1B and YopH separately (as described in the main text).

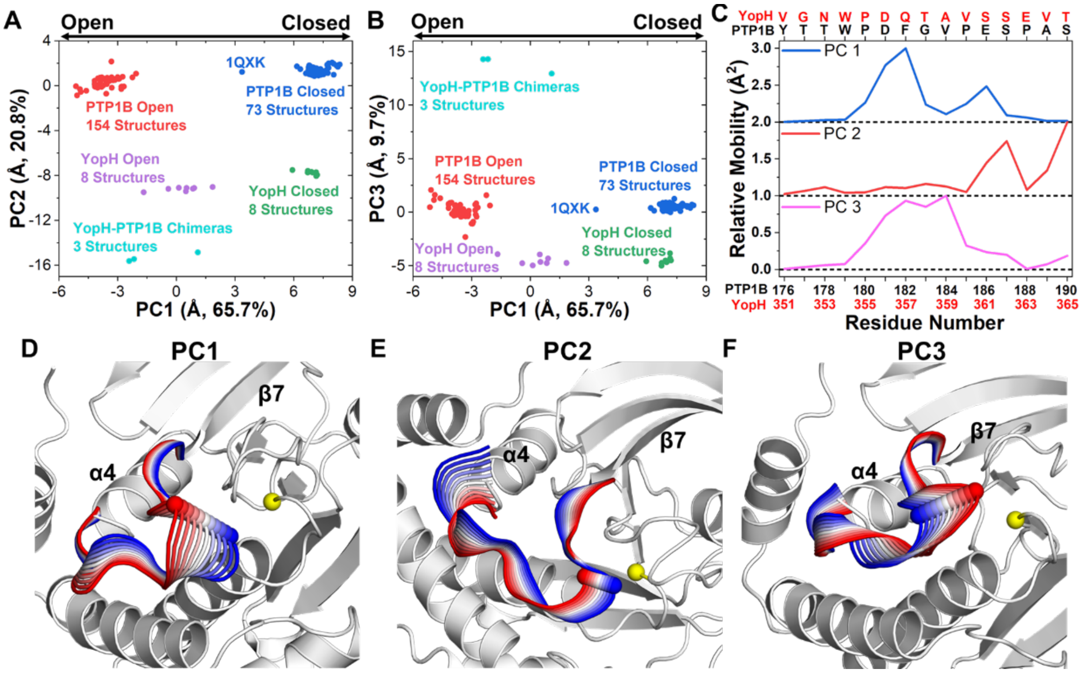

Figure SR1. Principal component analysis (PCA) on the WPD-loops of the crystal structures of PTP1B, YopH and the YopH-PTP1B chimeras (for details see main text). (**A**, **B**) Projections of crystal structures onto the first three principal components (PCs), with the total variance described by each PC indicated in brackets in the axis labels. (**C**) Relative mobility of each residue in each PC projection, with residue numbers and names provided above and below

respectively for both PTP1B and YopH. (**D–F**) Projection of PCs 1-3 onto a representative structure of PTP1B. The color gradient on the WPD-loop indicates moving from negative (red) to positive (blue) values along the given PC (as seen in panels **A**, **B**). The catalytic cysteine (yellow) and aspartic acid are shown as spheres for reference, and the locations of the β10-strand and α4-helix on PTP1B (which precede and follow the WPD-loop residues, respectively) are indicated in all three panels.

Hamiltonian Replica Exchange Sampling and Convergence

The analysis of the conformational sampling of our HREX-MD simulations of PTP1B and YopH shows limited sampling of the WPD-loop closed states for PTP1B and YopH (**Figure SR2**). In particular, after ~250 ns, essentially no sampling of the WPD-loop closed state is observed for YopH. These results would therefore suggest our simulations are unable to provide a reliable estimate of the free energy difference between the WPD-loop closed and open states.

We have previously observed a similar issue when studying the dynamics of an active site loop in the enzyme triosephosphate Isomerase (TIM) with HREX-MD simulations. Simulations of TIM were initiated from the loop closed state but within ~25 ns of simulation time all replicas had opened the loop and it was not observed to close again throughout the rest of the simulation. As is the case here, this is likely because although HREX-MD enhances the sampling to increase the probability of observing rare events (like loop conformational changes) the simulations are still relatively short when compared to the experimental loop closure rates (for both TIM and the two PTPs studied here). In contrast (and as also observed in our previous study of TIM), metadynamics simulations which use collective variables to enhance sampling did not suffer from this issue (see **Figures S3** and **S4** for evidence of "diffusive dynamics" in our PT-MetaD-WTE simulations).

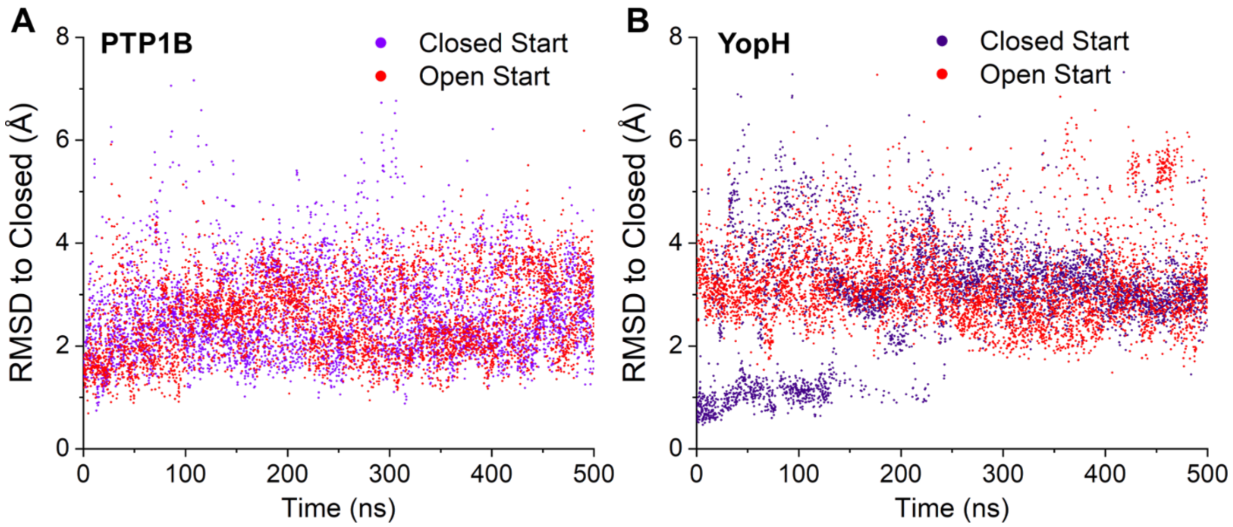

Figure SR2. The Cα RMSD of the WPD-loop to the closed X-ray crystal structures of PTP1B (**A**) and YopH (**B**) from the neutral replica of our HREX-MD simulations. For clarity, only every 50th snapshot is plotted. The structures 6B90[8] and 2I42[7] were used as reference for the WPD-loop closed states for PTP1B and YopH, respectively.

Changes in pK$_a$ along the WPD-loop Opening/Closing Pathway

Using our PT-MetaD-WTE trajectories of both PTP1B and YopH with *p*NPP-bound, we predicted how the pK_a of all titratable residues differs along the WPD-loop opening/closing pathway. To do this, we extracted snapshots from our trajectories at different native contact values along the WPD-loop opening/closing pathway. Using PROPKA v3.1,[10] we then calculated the average (from 100 snapshots at each point) pK_a predicted for each residue, with complete results for PTP1B and YopH provided in **Tables S14** and **S15** respectively. We note that whilst empirical methods like PROPKA are not reliable for exact pK_a values, they should be able to identify trends (in this case associated with WPD-loop conformational changes).[10] We observed no notable changes in the pK_a of any residue throughout both PTPs, and even in the case of the catalytic acids, the changes are quite subtle (the maximum difference was less than 1 unit for both PTPs). These

results are also consistent with our PROPKA predictions on crystal structures of open and closed state crystal structures of PTP1B and YopH (discussed in the **Supplementary Methods**).

Identification of Residues Directly Correlated to WPD-Loop Motion

To complement our enhanced sampling simulations, we performed structural bioinformatics analysis on the resulting PT-MetaD-WTE trajectories in order to explore potential pathways of allosteric communication throughout both PTPs. As we are particularly interested in how the motion of the WPD-loop is regulated by the remainder of the protein, we computed dynamic cross correlation matrices (DCCMs) for both PTPs (**Figure S13**) to identify which residues correlated with the motion of the WPD-loop (**Figure SR3**). DCCMs measure the degree of correlated motion between residues (using the Cα carbon of each residue for the measurement) over the course of the simulation. Motions between atoms are assigned values between +1 (perfectly positively correlated motion) and –1 (perfectly anti-correlated motion), and 0 indicating no correlation between residues. We also identified many residues that correlate with WPD loop motion for PTP1B to include those that make up both of the known allosteric drug binding sites on PTP1B (BB[70] and K197[8] allosteric sites, see **Figure SR3**). These results are of particular interest, given the relatively limited information on YopH allostery and the potential application of inhibitors towards YopH.[71–73]

We further note that comparison of the truncated DCCM plots obtained for unliganded, *p*NPP-bound and the phospho-enzyme intermediate simulations of PTP1B and YopH (**Figure S14**) are largely similar, with the unliganded state generally showing slightly higher correlation than the remaining two states. However, the same sets of residues are indicated for each state, suggesting the same allosteric network is active in all three states. Specifically, for PTP1B, we identified residues on the α3-, α6- and α7-helices; the β5-, β6- and β7-strands, and the E-Loop (~residues

109-129). For YopH, we identified residues on the α1-, α4- and α7-helices; the β5-, β6- and β7-strands, and the E-Loop (~residues 284-304). Interestingly, despite their close spatial proximity and catalytic importance (see **Figure 1**), the motion of the P-loop is not notably correlated with WPD-loop motion in either PTP1B or YopH (**Figure SR3**). As mentioned in the main text, in contrast to the P-loop, however, and of interest considering the data presented in **Figure 5**, we find the motions of the WPD-loop and E-loop to be correlated with one another.

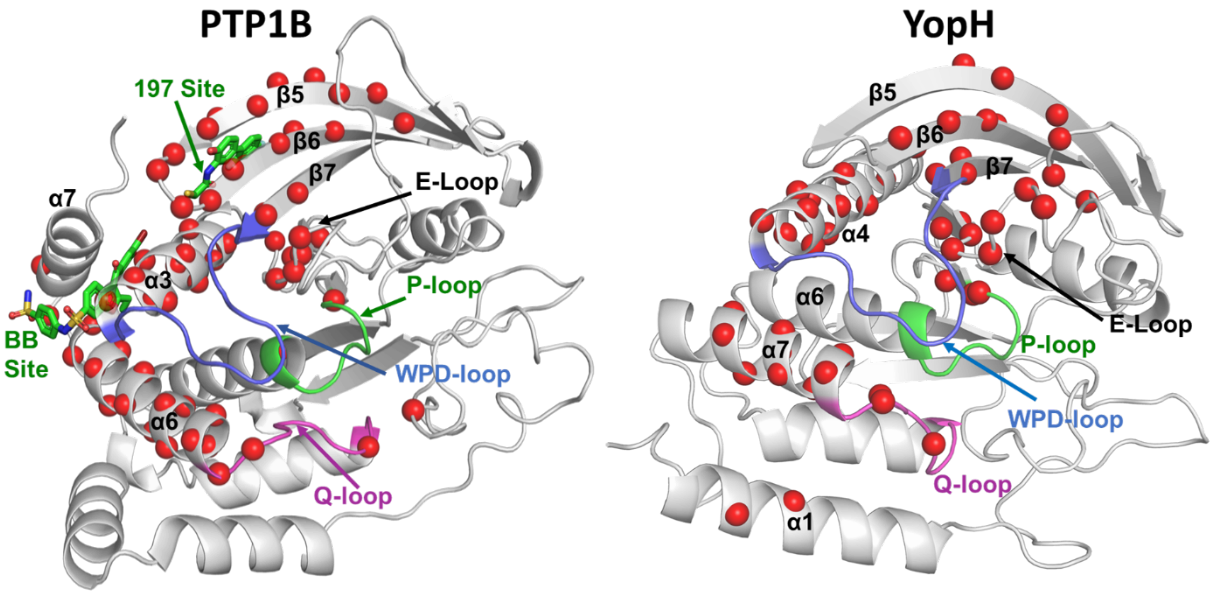

Figure SR3. Residues in PTP1B and YopH that are directly correlated with WPD-loop motion identified from our PT-MetaD-WTE simulations of PTP1B and YopH with *p*NPP-bound. Correlated residues (shown as red spheres) were identified by calculating dynamic cross correlation matrices (DCCMs) for both PTPs and identifying any residue with a correlation cut-off value >|0.3| to any WPD-loop residue (meaning that both correlated and anti-correlated residues are shown). The WPD-loop, P-loop and Q-loop are colored in blue, green and magenta respectively, and key secondary structure regions are also labelled. The two known allosteric drug binding sites (BB and 197) are also depicted with a representative drug bound in each position (using PDB IDs: 1T49[70] and 6B95[8] respectively). Truncated DCCM plots of PTP1B and YopH in all three simulated states are provided in **Figure S14**.

Further Description of the SPM Generated for PTP1B

This section provides further details and comparisons to relevant experimental data for the Shortest Path Map (SPM)[44] generated for PTP1B, see discussion surrounding **Figure 6** in the main text. Our SPM analysis of PTP1B identifies a central "highway" of key residues (H175, M109 and H214) which contain four major branches spouting off from this highway (**Figure 6A**). One of these branches begins from the α7-helix (H296 and E297), passing through Loop 11 residues (including Y152 and Y153) towards the N-terminus of the WPD-loop (H175). The observation that allosteric activation of the α7-helix is communicated through Loop 11 through to the N-terminus of the WPD-loop is consistent with prior NMR and structural studies,[8,74] and the SPM analysis allows us to characterize the key residues and pathways involved in this allosteric activation. Known allosteric mutations A69V and R56V form another branch to the central highway, passing through residues on the β3-strand (T84), P-loop (H214) and Loop 9 (M109) before reaching the N-terminus of the WPD-loop. Note that analogous mutations at equivalent positions in other PTPs (SHP2 and TC-PTP) also significantly alter activity.[75] Another branch starts between the α1 and α2-helices, and passes through the C-terminal region of the α6-helix, followed by the Q-loop before finally reaching the central highway residue H214 on the P-loop. Mutations G259Q (on the Q-loop) and R254A (on the α5-helix) likely interfere with this pathway of communication. The final branch identified begins at F135, travels through the α3-helix (*i.e.*, residues the G92 and C93) and Loop 3 (*i.e.* P87 and G85) before reaching H214.

Impact of Substrate Restraints on the Michaelis Complexes Stability.

Both our conventional MD and PT-MetaD-WTE simulations with *p*NPP-bound utilized several one-sided harmonic restraints (commonly referred to as "wall potentials", **Table S6**) between the P-loop and the phosphate group of the substrate. The rationale for this was to prevent

the dissociation of the substrate so as to ensure we are studying only the ligand bound state throughout the simulation(s). We note as well there is strong experimental evidence that a substrate can bind independent of WPD-loop conformation,[70,76] (*i.e.*, the loop does not need to be closed for substrate binding to occur).

Based on a reviewer comment, in this section we have evaluated how stable the substrate (*p*NPP) is in the absence of any restraints. To do this, we performed 20 replicas of 200 ns long MD simulations of PTP1B and YopH with *p*NPP-bound starting from the WPD-loop closed state. We have compared these results to an equivalent amount of simulation time using our prior protocol with restraints applied between the P-loop and *p*NPP (**Figure SR4**).

Analysis of **Figure SR4A** and **B** unsurprisingly shows that the simulations performed with restraints maintain the reactive phosphorus sulfur distance to a narrow range for both PTPs. In contrast, simulations of both PTPs with *p*NPP-bound in the absence of restraints show sampling of a mixture of both reactive and non-reactive phosphorus-sulfur distances (**Figure SR4C** and **D**). In order to examine substrate binding in the non-productive conformations, we clustered our PTP1B trajectories (**Figure SR4E** and **F**), and found that in the extended phosphorus-sulfur distance sampled in our simulations is due to rotation of the substrate away from an optimal conformation for inline nucleophilic attack. The non-productive conformation (**Figure SR4F**) identified through clustering is still able to interact with the arginine side chain and many other hydrogen bond donors which ultimately stabilize this conformation. Given the small size of the substrate and the fact that it interacts with both PTPs almost solely through its phosphate group, its limited stability at the Michaelis complex is perhaps therefore of little surprise.

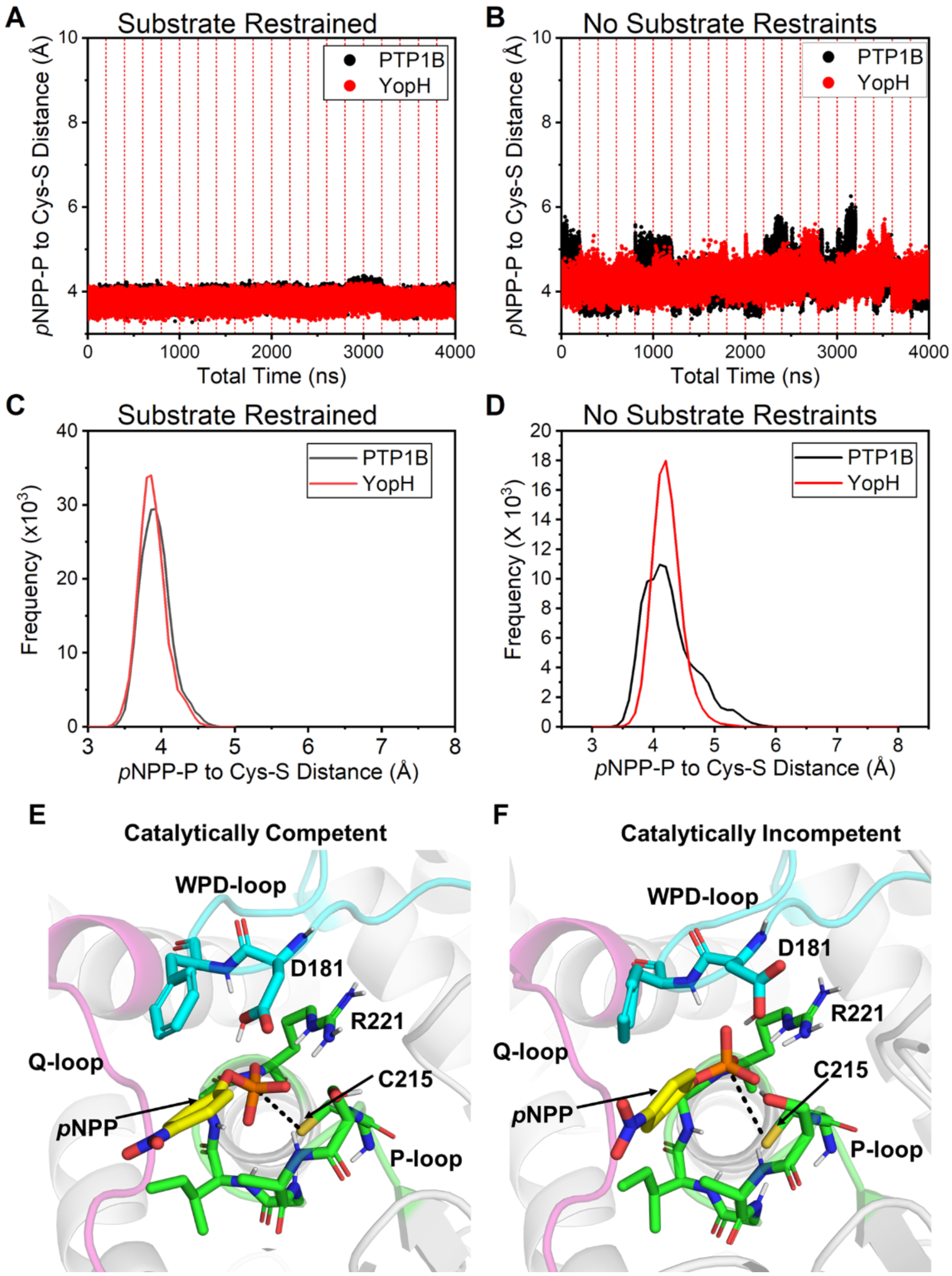

Figure SR4. Impact of substrate restraints on the sampling of *p*NPP. (**A**, **B**) Distance between the phosphorous atom of *p*NPP and the sulfur atom of the catalytic cysteine over the course of our MD simulations with the restraints between the P-loop and *p*NPP as defined in **Table S6** both (**A**) with and (**B**) without any restraints applied. In both cases 20 replicas each of length 200 ns are shown (with the red dotted lines marking out the start of a new trajectory). (**C**, **D**) Histograms of the distance described in panels **A** and **B** both (**C**) with and (**D**) without restraints applied. (**E,F**) Representative structures of catalytically competent and catalytically incompetent substrate binding modes observed

in simulations of PTP1B, respectively, without any restraints applied during the simulations. Representative structures were obtained through clustering with the DBSCAN[77] algorithm (epsilon set to 1), using all heavy atoms of the substrate (after first aligning the trajectory onto a stable portion of the scaffold).

Impact of Performing the EVB Simulations as One Continuous Process or Two Discrete Steps

For a typical EVB simulation protocol with multiple reaction steps, we would normally directly model the second step (hydrolysis step in this case) using the structure obtained from the end-point of the first step of the EVB simulations (which would be the phospho-enzyme intermediate). However, in between the cleavage and hydrolysis steps, the side chain rotamer of a conserved glutamine on the Q-loop (residues Q262 and Q446 for PTP1B and YopH, respectively, see **Figure 1**) changes from originally pointing away from the active site to coordinating the nucleophilic water molecule for the hydrolysis step (see detailed discussion in ref. 9). We do not observe this displacement at the end point of our EVB simulations of the cleavage reaction (**Figures 9** and **S17**), likely due to the product (*p*-nitrophenol) still being located in the active site of the enzyme. As this rotamer change is observed in PDB IDs: 3I80[9] and 2I42[7] (PTP1B and YopH, respectively), which were crystallized in the presence of vanadate ion as a proxy for the phospho-enzyme intermediate, we modelled the two reaction steps as discrete processes with the appropriate starting crystal structure for each process (see the **Methodology** section), and also with the *p*-nitrophenol leaving group removed from the starting point for the hydrolysis reaction, as this would be expected to depart rapidly from the active site.[78] This gives rise to the six key stationary points presented in **Figures 9** and **S16**. We note here that for comparison, we also modelled the cleavage step using the end-point of our EVB simulation (which does not include the motion of Q446 into the active site in the case of YopH). In doing so, we obtain an identical activation free energy in the case of PTP1B (14.2 ± 0.4 kcal mol^{-1}), whereas in YopH we obtain a higher activation free

energy (17.6 ± 0.3 kcal mol^{-1}) when Q446 is not in a catalytically competent conformation, in agreement with structural data.

Analysis of Reacting Distances From our EVB Simulations

We have explored structural changes observed in our EVB simulations of the different reaction steps and systems. In terms of transition state geometries (**Table S10**), we observe very similar P-O distances to either the leaving group in the cleavage or nucleophile in the hydrolysis step between the non-enzymatic and enzymatic reactions (irrespective of enzyme). However, we observe a slight contraction in the S_{Cys}-P distances with Pauling bond orders between the sulfur atom of the cysteine side chain and the phosphorus atom of the phosphate group in both PTP1B and YopH, compared to the non-enzymatic reaction. This difference becomes clear when considering Pauling bond orders of the transition state ensembles (determined from the calculated distances provided in **Table S10** and the equilibrium bond distances used in our EVB simulations, using the relationship $r = r_e - 0.6ln(n)$, see the **Supplementary Information Methods**). These are 0.42, 0.58 and 0.63 for the non-enzymatic reaction and the PTP1B and YopH catalyzed reactions, respectively, in the cleavage step, and 0.53, 0.76 and 0.80 in the hydrolysis step. For the P-O_{pNPP} distance in the cleavage step, the differences are much smaller, whereas for the hydrolysis step, the P-O_{H2O} bond orders follow a similar trend to the S_{Cys}-P distances (0.50 to 0.40 to 0.38 for the three different reactions, respectively). From this analysis based on Pauling bond orders (see full data in **Table S10**), it is clear that (aside from the differences between the non-enzymatic and enzyme catalyzed reactions), the main transition state differences between PTP1B and YopH are observed in the sulfur-phosphorus distances, for both reaction steps.

S3. Supplementary Figures

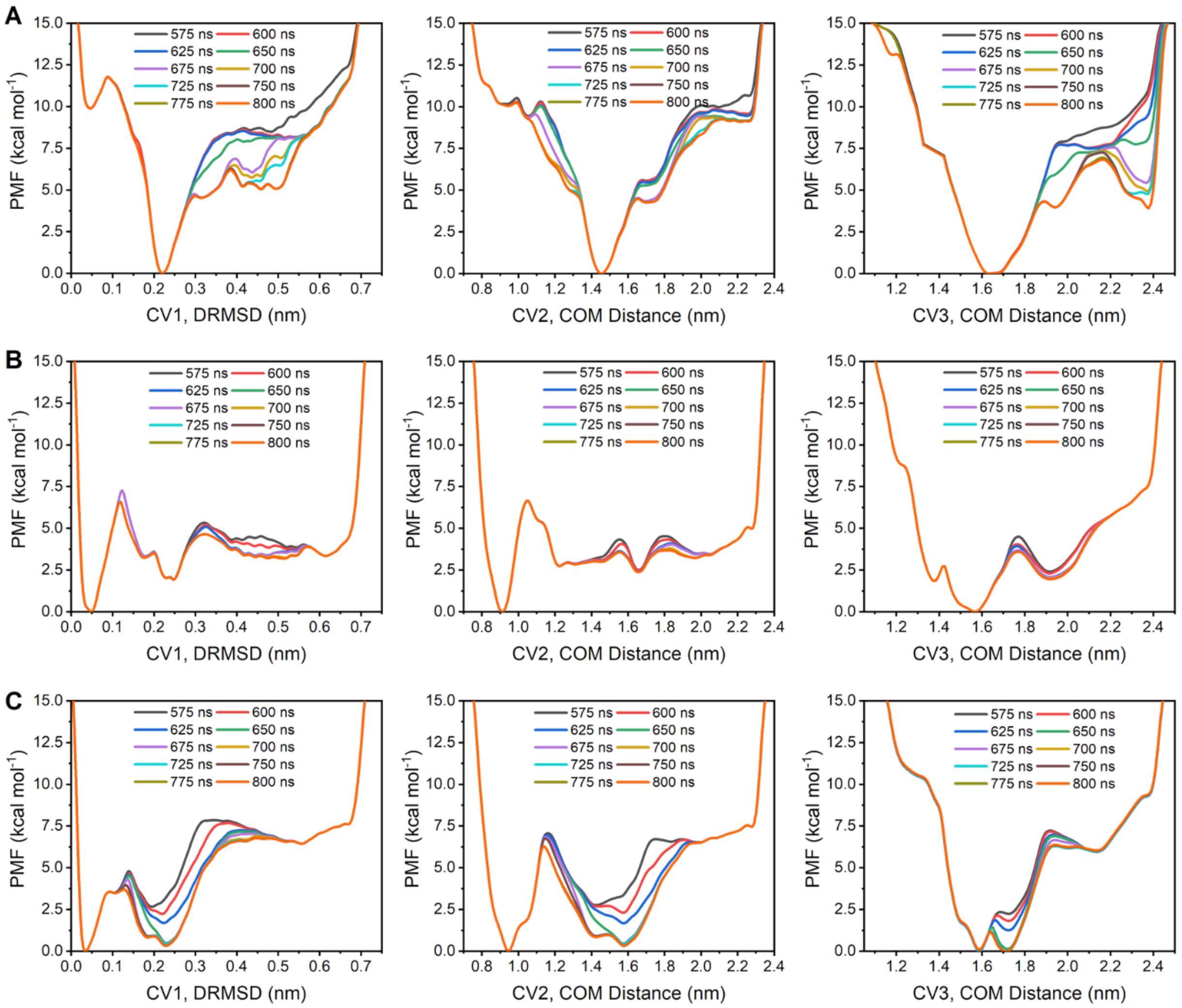

Figure S1. Time evolution of the free energy for the 3 collective variables (CVs) used for our PT-MetaD-WTE simulations of PTP1B in the (**A**) unliganded, (**B**) *p*NPP-bound and (**C**) phospho-enzyme intermediate states. For a definition of the CVs used, see **Table S7**.

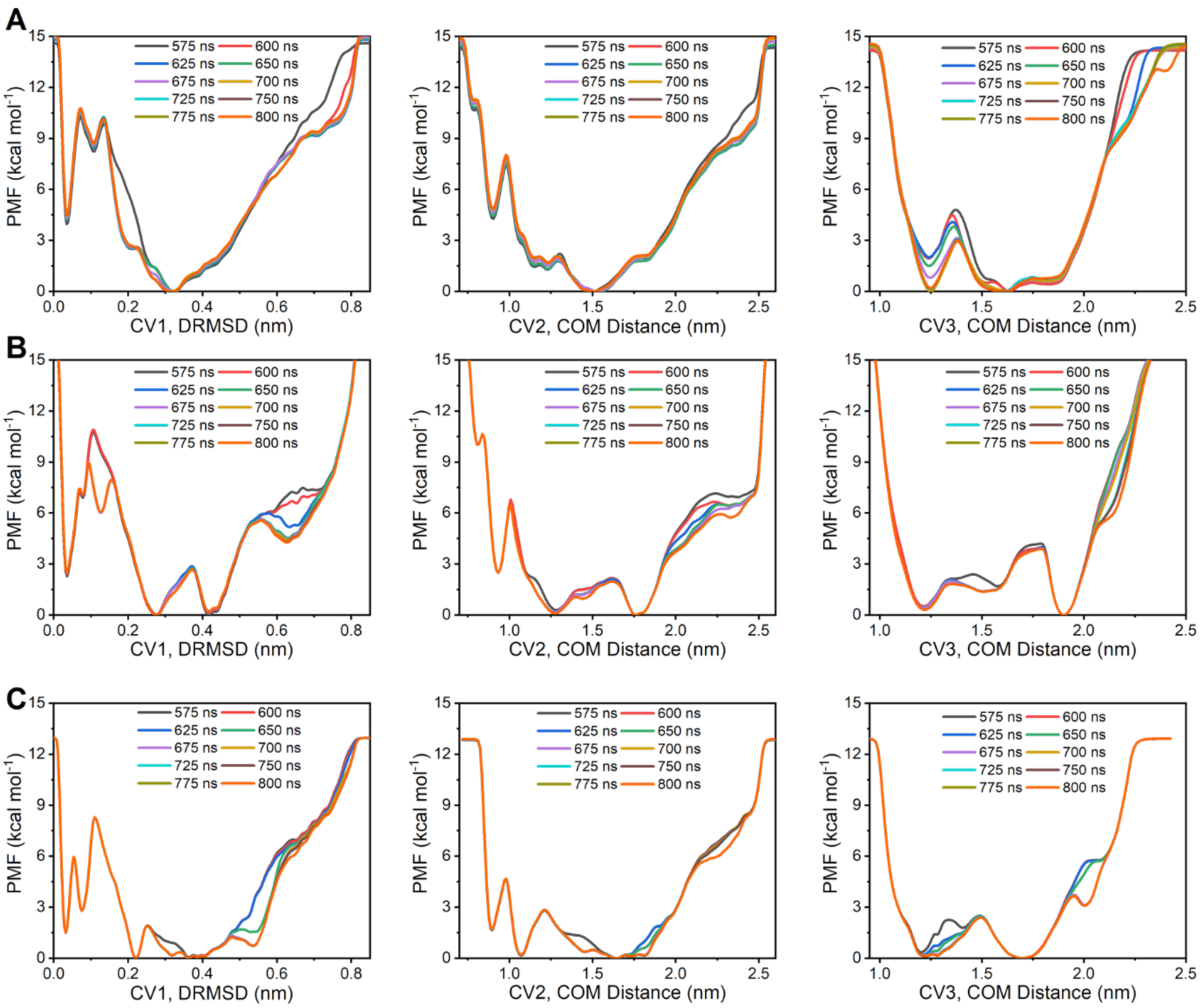

Figure S2. Time evolution of the free energy for the 3 collective variables (CVs) used for our PT-MetaD-WTE simulations of YopH in the (**A**) unliganded, (**B**) *p*NPP-bound and (**C**) phospho-enzyme intermediate states. For a definition of the CVs used, see **Table S8**.

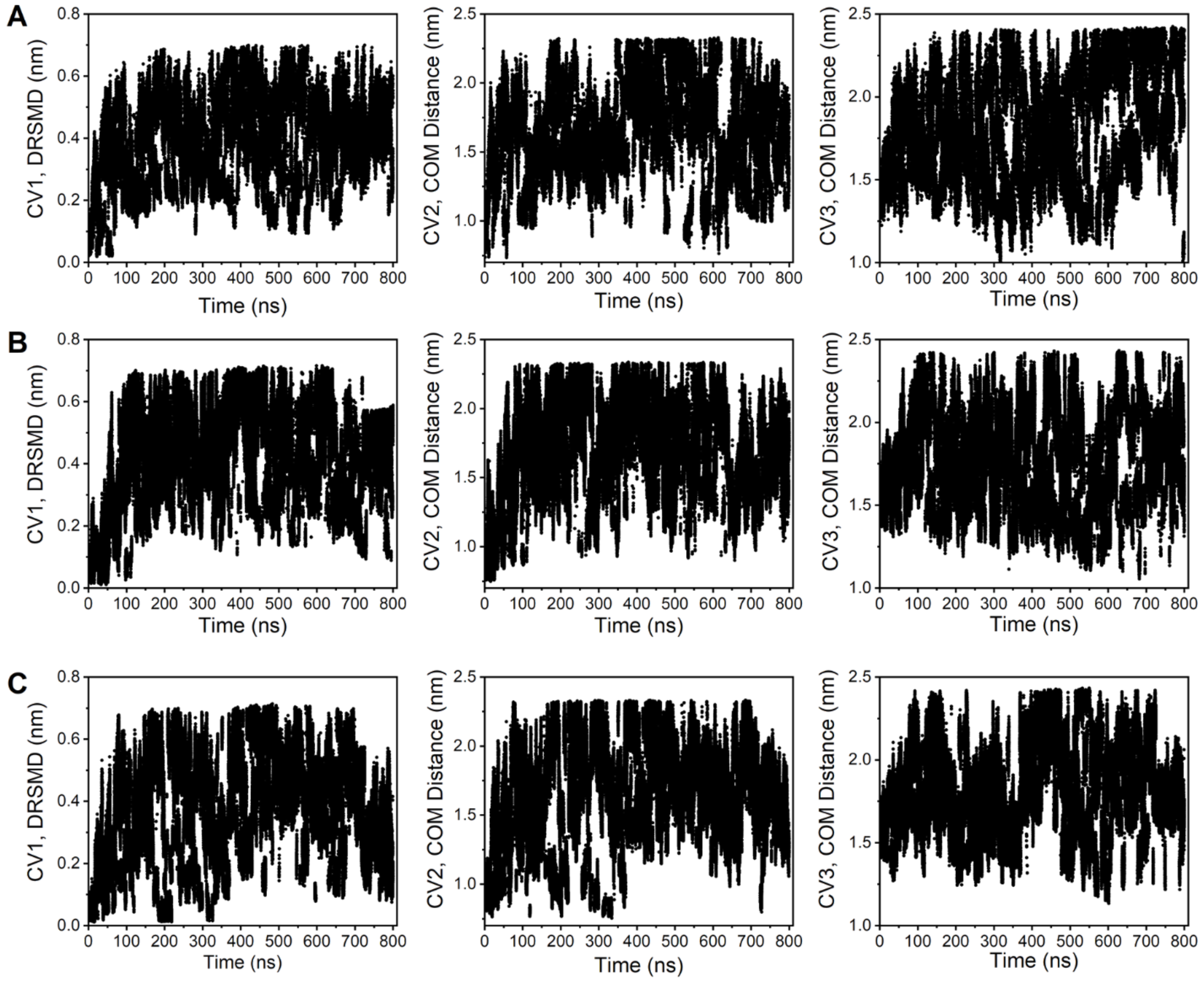

Figure S3. Diffusive dynamics of the 3 collective variables (CVs) used for our PT-MetaD-WTE simulations of PTP1B in the (**A**) unliganded, (**B**) *p*NPP-bound and (**C**) phospho-enzyme intermediate states. Note that wall-potentials were used on each CV to prevent the sampling of conformational states beyond those observed in our HREX-MD simulations (see **Methodology** and **Supplementary Methodology** for further details). For a definition of the CVs used, see **Table S7**.

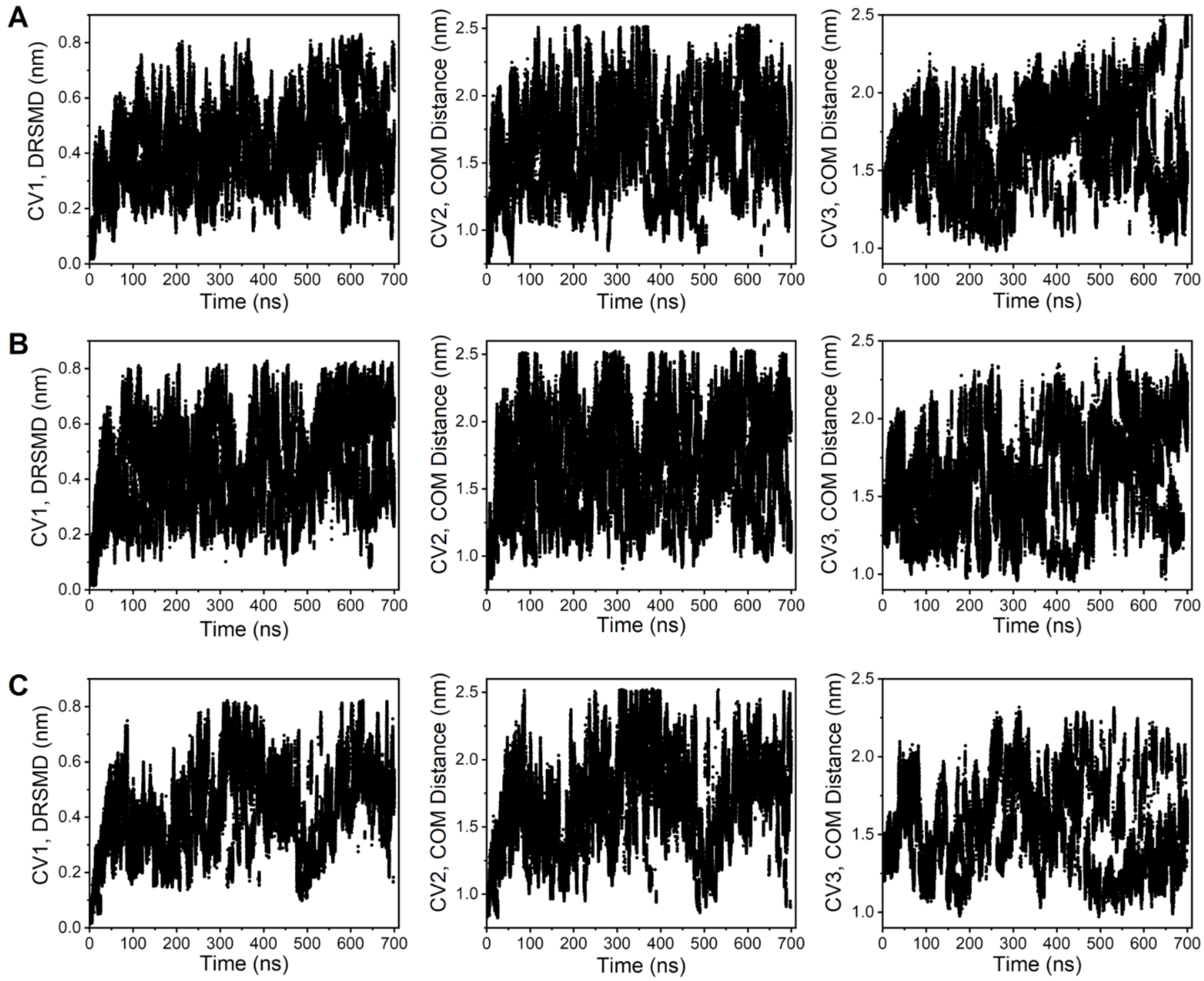

Figure S4. Diffusive dynamics of the 3 collective variables (CVs) used for our PT-MetaD-WTE simulations of YopH in the (**A**) unliganded, (**B**) *p*NPP-bound and (**C**) phospho-enzyme intermediate states. Note that wall-potentials were used on each CV to prevent the sampling of conformational states beyond those observed in our HREX-MD simulations (see **Methodology** and **Supplementary Methodology** for further details). For a definition of the CVs used, see **Table S8**.

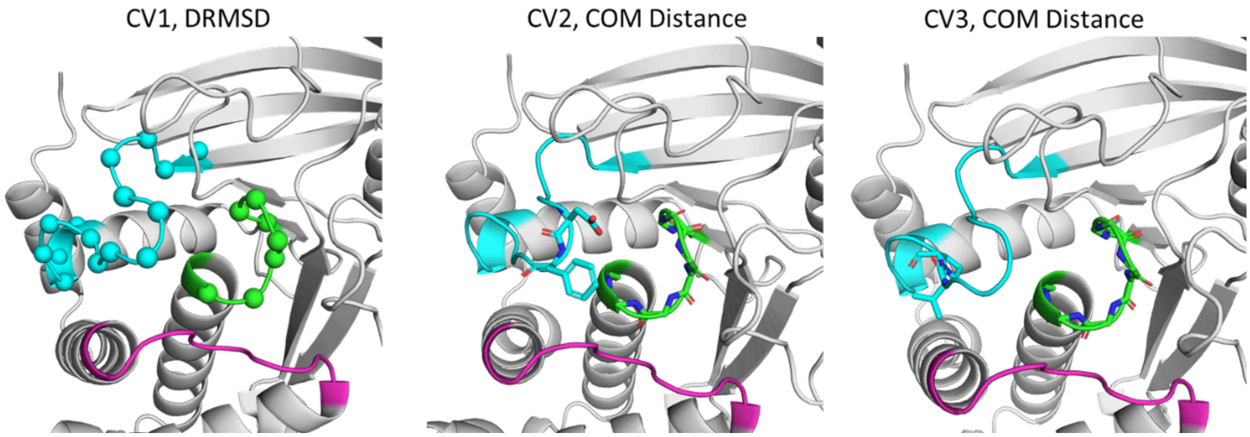

Figure S5. Graphical depicition of the collective variables (CVs) used to describe WPD-loop motion in PTP1B (note that effectively the same sets of CVs were used to descrbe YopH). CV1 is the inter-distance RMSD (DRMSD) between the C_α of the WPD-loop to the P-loop. CV2 is the center of mass (COM) distance between the WPD-loop residues D181 and F182 to the backbone heavy atoms of the P-loop. CV3 is the COM distance between the C-termial residues of V184 and P185 to the backbone of the P-loop R221. Note that the P-loop is highly stable in both enzymes (and throughout the metadynamics simulations), and so acts as a good reference point. The WPD-loop, P-loop and Q-loops are coloured cyan, green and purple respecively. A more detailed list of CVs and the corresponding parameters used can be found in **Table S7** for PTP1B, and **Table S8** for YopH.

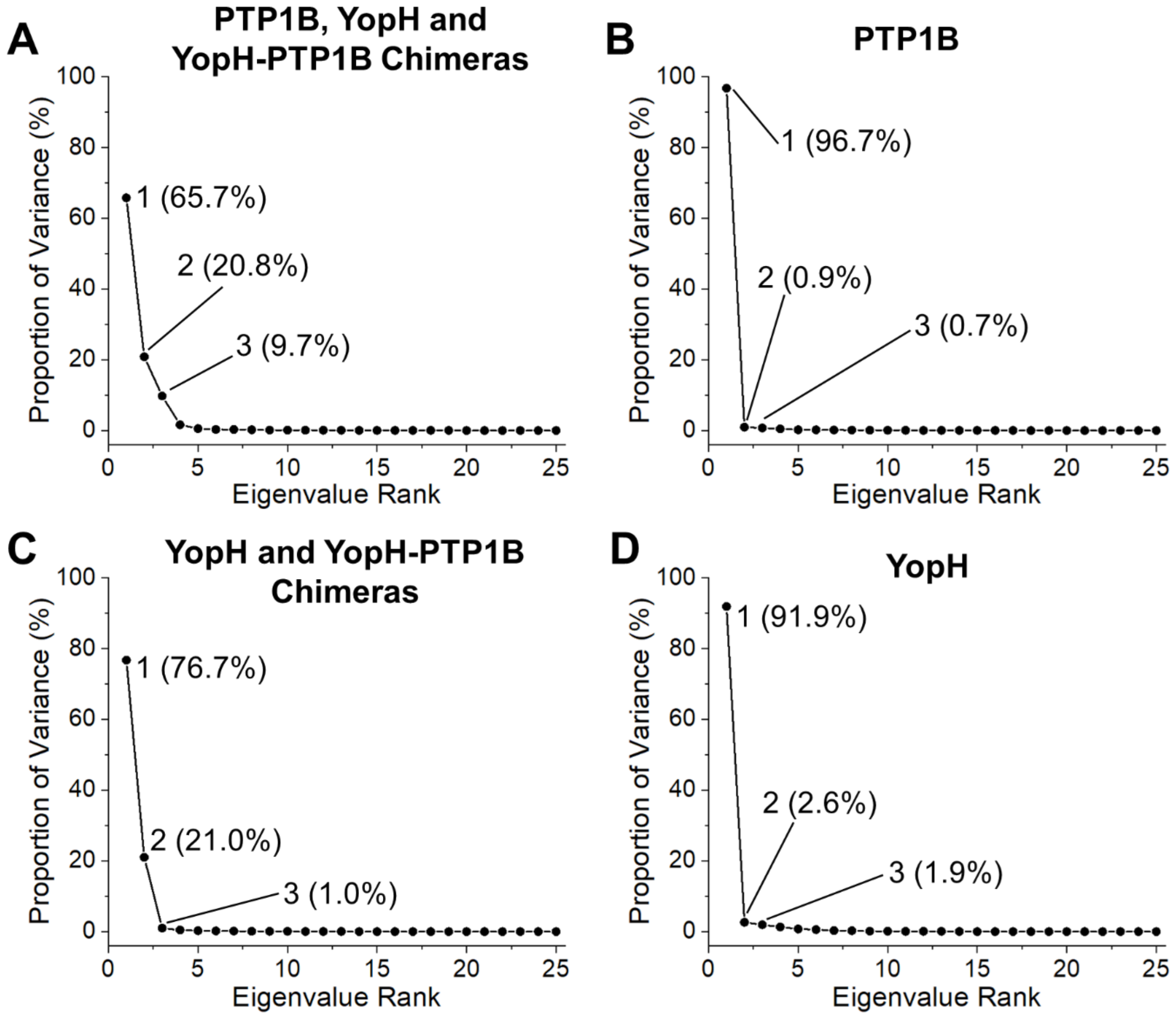

Figure S6. The percentage variance described by each principal component (PC) from PCA performed on the WPD-loops of X-ray structures of PTP1B and YopH, as well as YopH-PTP1B chimeras.[4] The values of the three principal components with the largest eigenvalues (*i.e.*, the largest description of the variance) are stated on each graph.

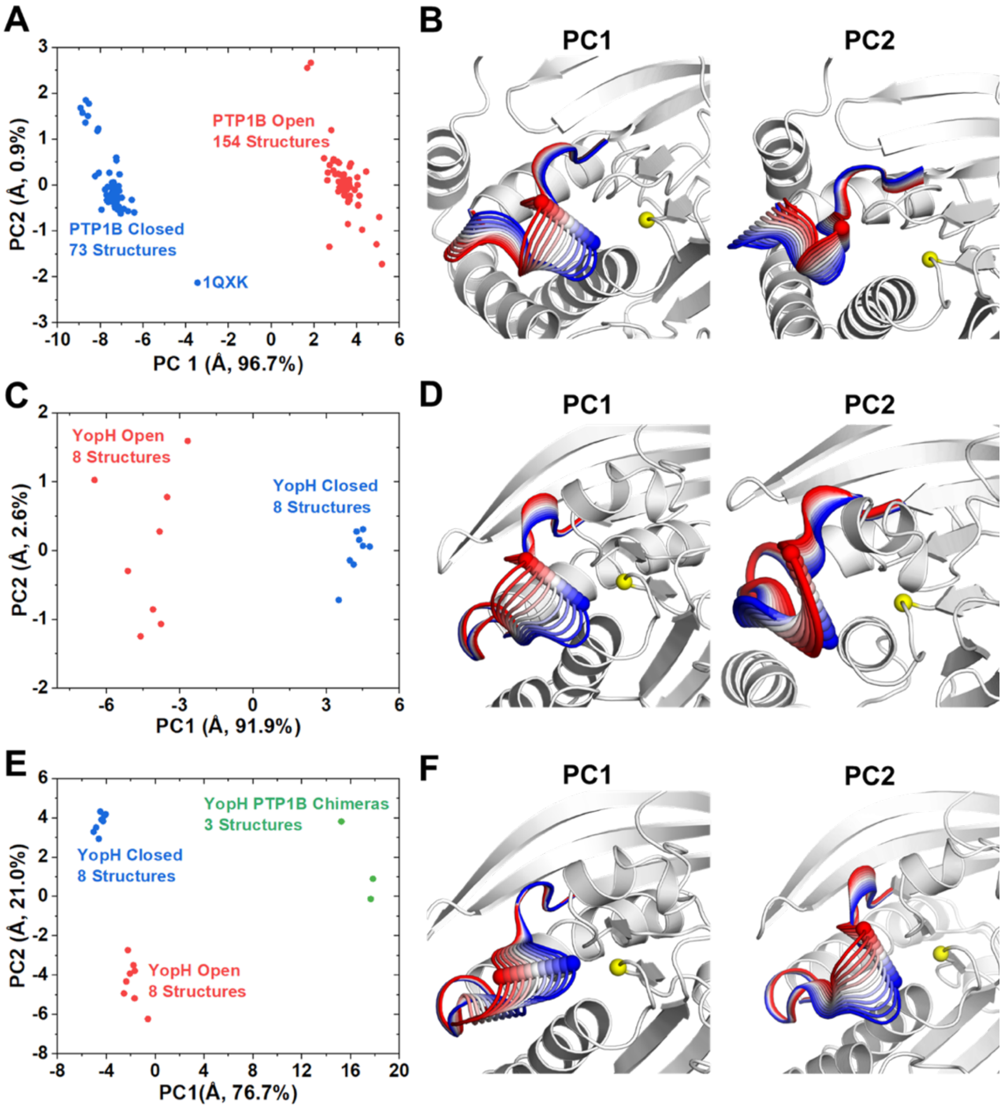

Figure S7. PCA of the WPD loops of the X-ray structures of (**A**,**B**) PTP1B, (**C**,**D**) YopH and (**E**,**F**) YopH and YopH-PTP1B chimeras.[4] For a list of PDB structures used for the analysis, see **Table S2**. The total variance described by each PC is provided in brackets on the axis labels. The first two PCs are projected onto each PCA. In all cases, the catalytic aspartic acid and cysteine nucleophile are shown as balls on each structural figure.

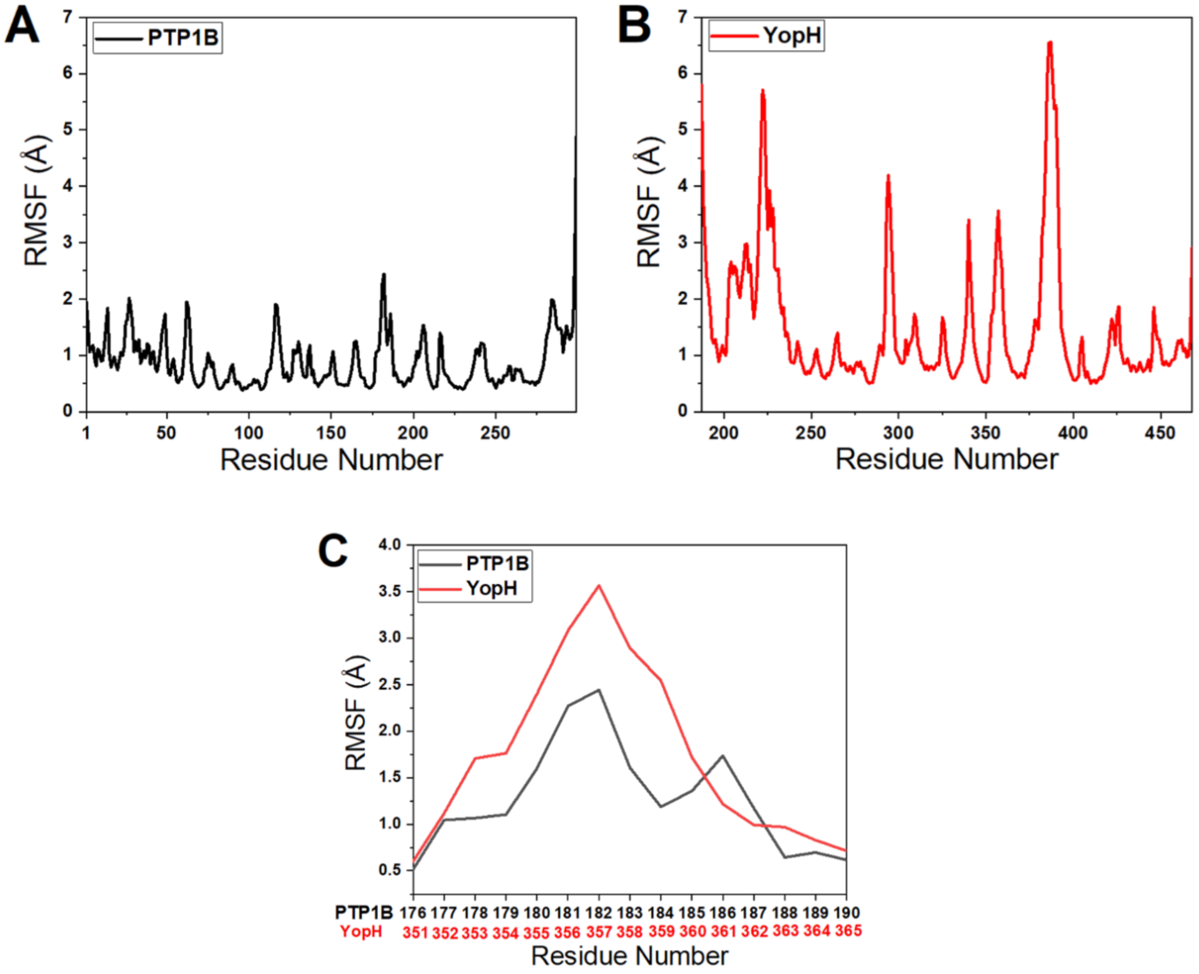

Figure S8. (**A**, **B**) Root mean square fluctuations (RMSF, Å) of all C_α atoms of the unliganded forms of (**A**) PTP1B and (**B**) YopH, obtained from HREX-MD simulations of these systems. (**C**) The RMSF of the WPD-loop regions for both enzymes. For PTP1B, RMS fitting was performed to the C_α atoms of residues: 15-26, 33-41, 69-84, 91-102, 106-109, 133-150, 153-162, 166-176, 188-201, 212-214, 220-237, 246-256, and 264-281 after generating an average structure from the HREX-MD trajectory. For YopH, RMS fitting was performed to the C_α atoms of residues: 191-208, 246-251, 254-259, 264-277, 281-284, 288-296, 306-308, 311-324, 327-337, 344-351, 362-386, 389-392, 400-402, 408-420 and 429-440 after generating an average structure from the HREX-MD trajectory.

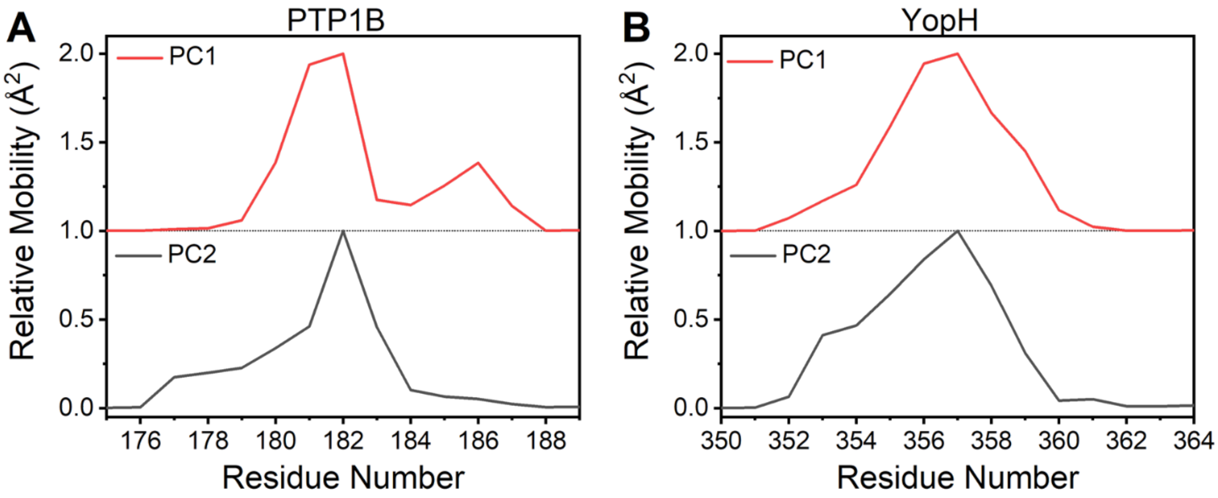

Figure S9. Mobility plots of PC1 and PC2 from the PCA performed on our HREX-MD simulations of the unliganded-states of (**A**) PTP1B and (**B**) YopH.

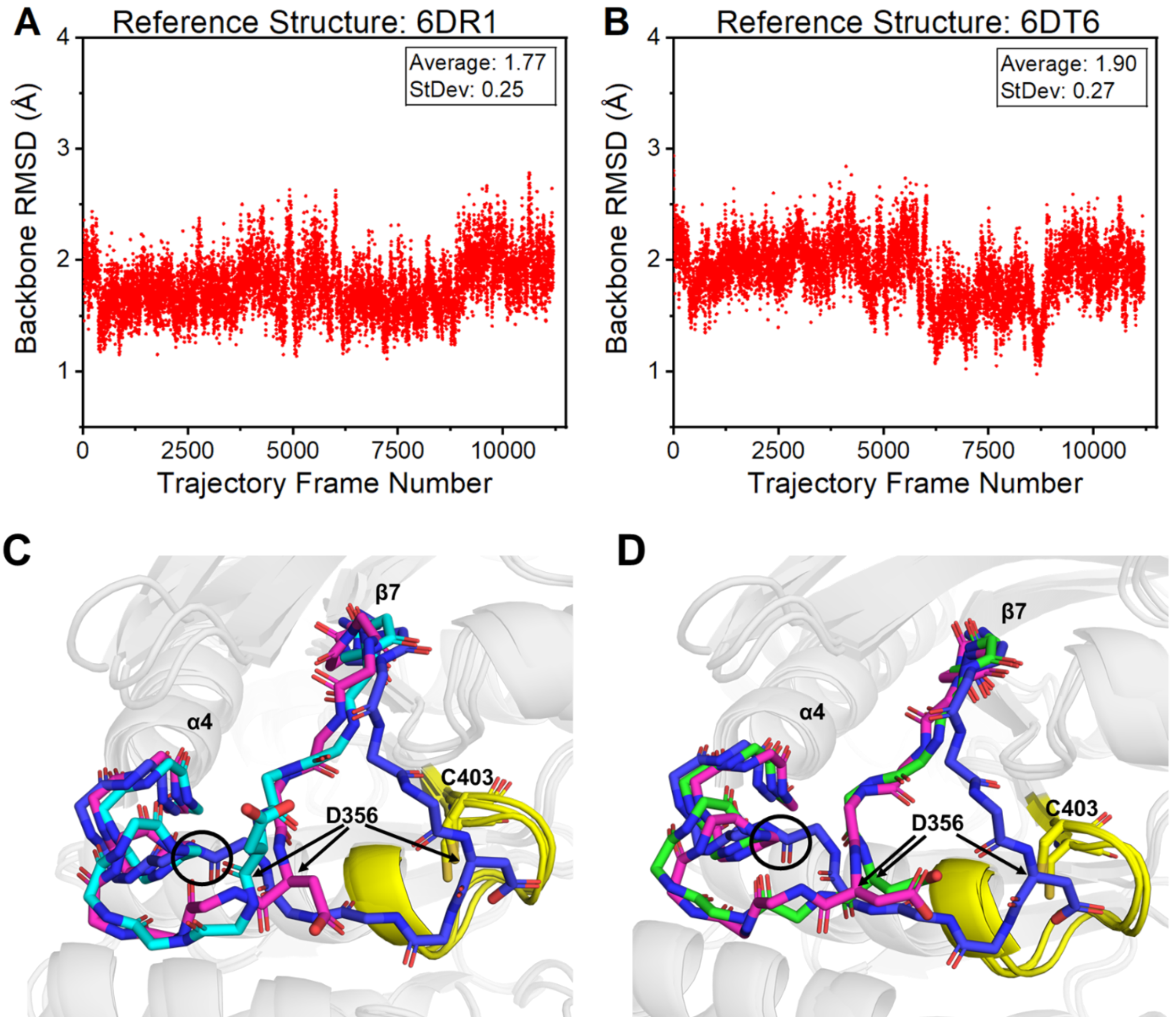

Figure S10. (**A**, **B**) Measurement of the backbone WPD-loop RMSD of WT-YopH to the crystal structures of two YopH-PTP1B chimeras which adopt "hyper-open" WPD-loop conformations (PDB IDs: 6DR1[4] and 6DT6,[4] respectively). Trajectory frames used are those with their WPD-loop in an extended α-helix conformation from our HREX-MD simulations (see **Figure 3** and the associated discussion in the main text). PDB IDs: 6DR1[4] and 6DT6[4] (referred to as chimera 2 and chimera 3 in the original manuscript[4]) are used as the reference structure for RMSD calculations in panels **A** and **B** respectively. Selected frames were fitted to the reference structure using the C_α atom of residues: 191-208, 246-251, 254-259, 264-277, 281-284, 288-296, 306-308, 311-324, 327-337, 344-351, 362-386, 389-392, 400-402, 408-420 and 429-440. The RMSD of the WPD-loop

was then determined using the backbone of residues 351-365. (**C**, **D**) Aligned structures of HREX simulation of WT-YopH (shown in magenta) with the smallest backbone WPD-loop RMSD to the two crystal structures of the wide open YopH-PTP1B chimeras. The crystal structures of chimera 2 (PDB ID: 6DR1,[4] panel **C**) and chimera 3 (PDB ID: 6DT6,[4] panel **D**) are shown in cyan and green respectively. The WT-YopH closed state is shown in dark blue below for reference. For both figures, the P-loop is colored yellow and the point at which helix extension occurs in the wide-open states (see main text) is circled for both figures. Further, the β7-sheet and α4-helix, which precede and follow the WPD-loop, are indicated on the figure.

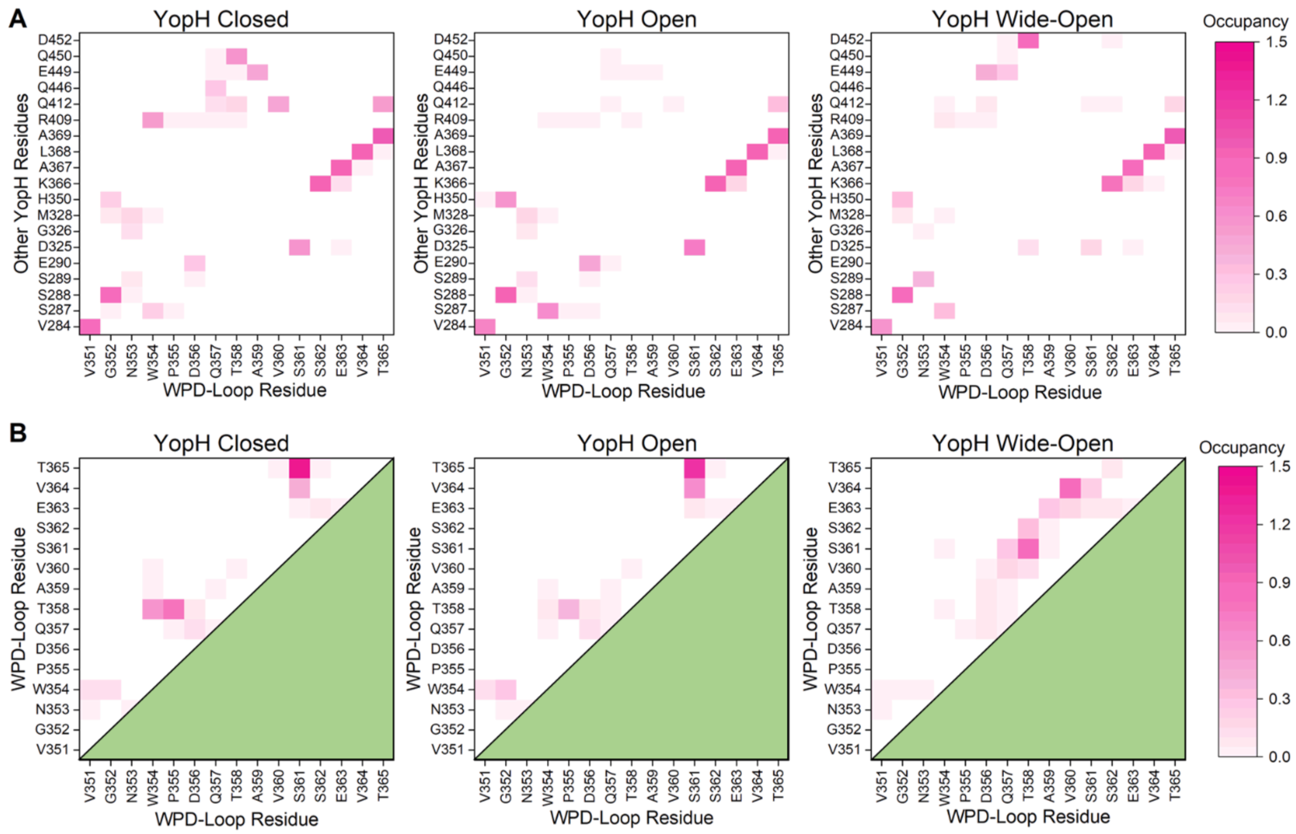

Figure S11. Heat maps of the WPD-loop hydrogen bonding (H-bond) network differences for YopH in the closed, open and hyper-open WPD-loop conformational states, obtained from our HREX-MD simulations. The top panels describe the H-bonds between WPD-loop residues and other residues distributed throughout the entire enzyme, while the bottom panels describe inter-WPD-loop H-bonds (note that in the bottom panels, only half of the matrix is shown, as it is symmetrical). The heat map is colored according to the average number of H-bonds formed per frame between each residue.

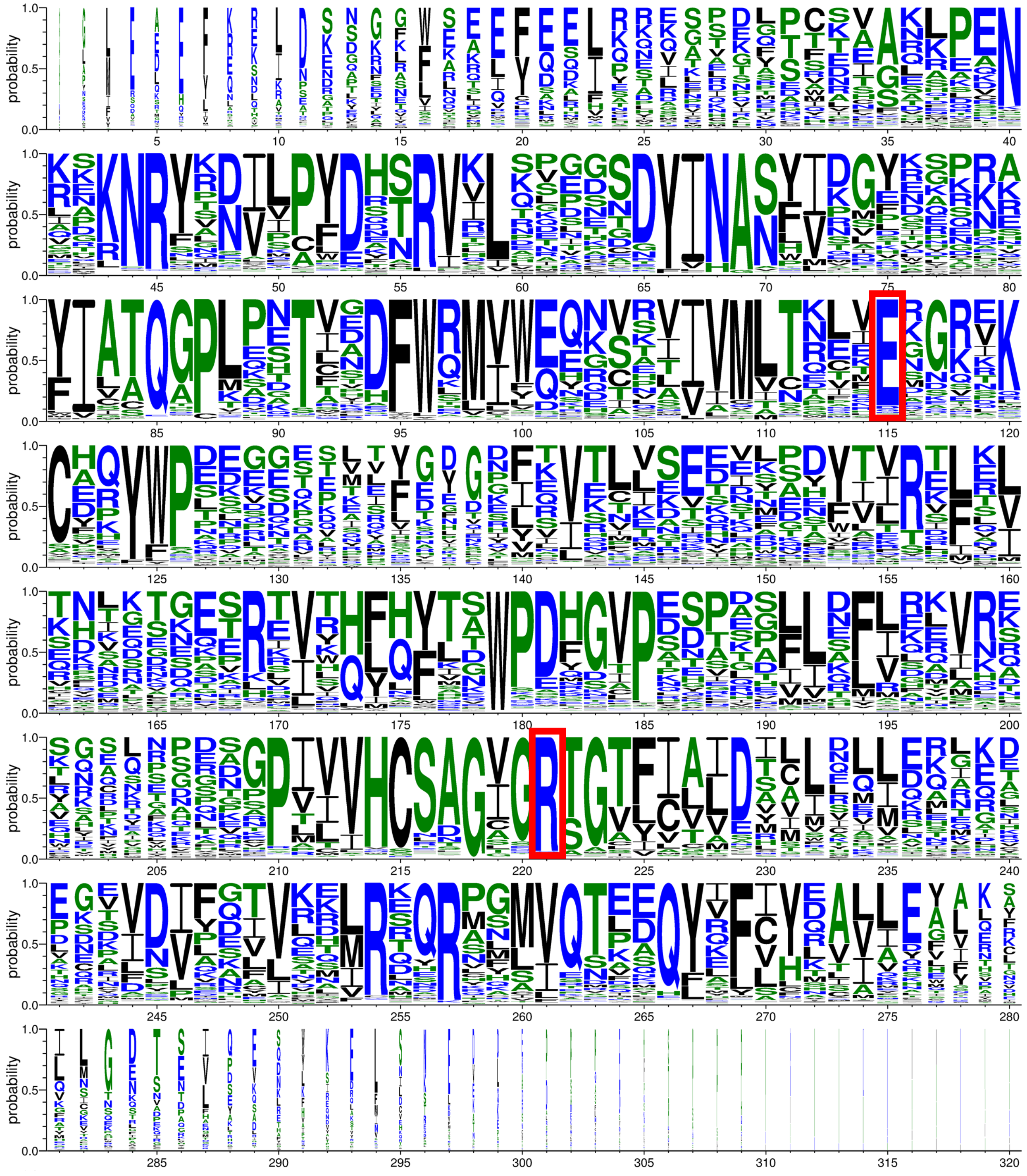

Figure S12. Measurement of sequence conservation among PTPs. The probability of a amino acid residue for a given position is depicted using the protein sequence of PTP1B as the template.

Residues E115 and R223 (both highlighted with red boxes) are highly conserved at their respective positions, and form the salt bridge between the P-loop and Loop 5 (see the main text for further discussion). The sequence logo was generated using the GREMLIN[79] webserver (http://gremlin.bakerlab.org) and the PTP1B amino acid sequence, using default settings.

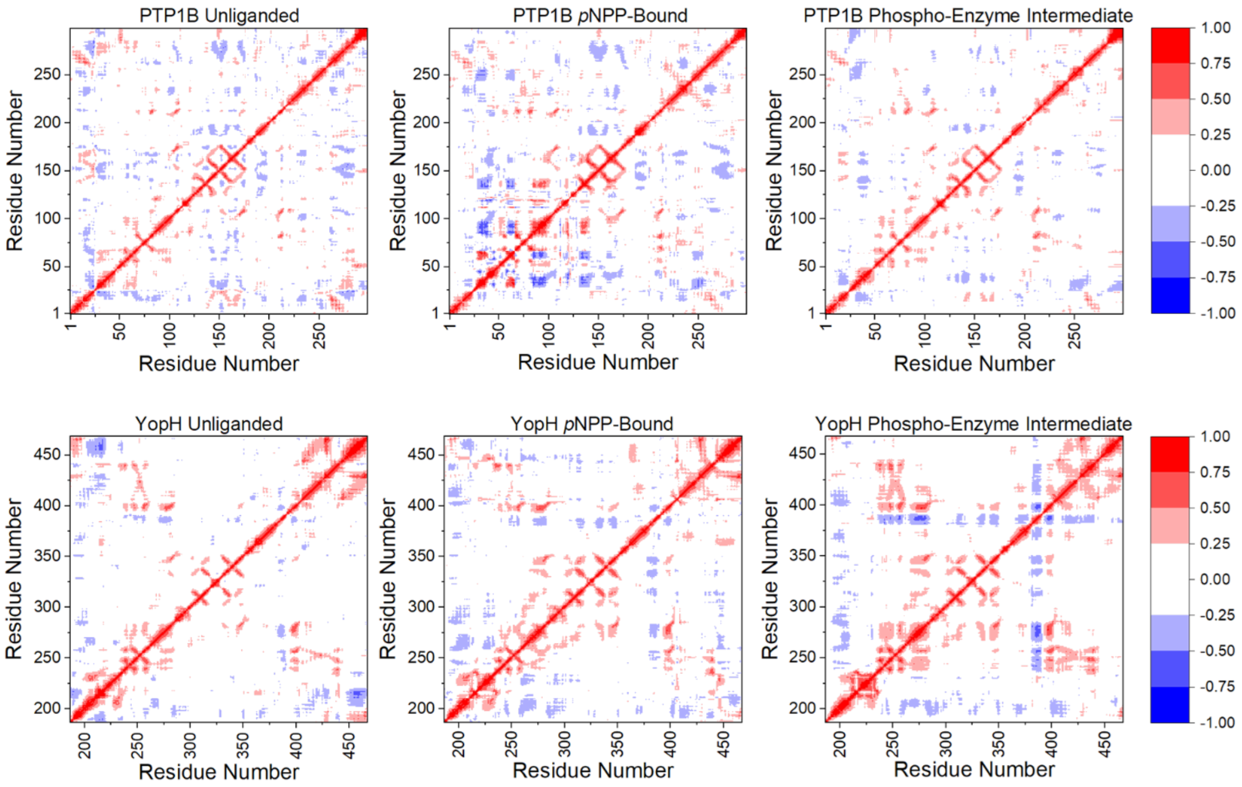

Figure S13. Dynamic cross correlation matrices (DCCMs) obtained from our PT-MetaD-WTE simulations of PTP1B and YopH in their unliganded, *p*NPP-bound and phospho-enzyme intermediate states. DCCMs were obtained using the C_α carbon of each residue and are colored coded as indicated by the color bars on the left-hand side of each row. A value of +1 (red) indicates perfectly correlated motion, whilst -1 (blue) indicates perfectly anti-correlated motion.

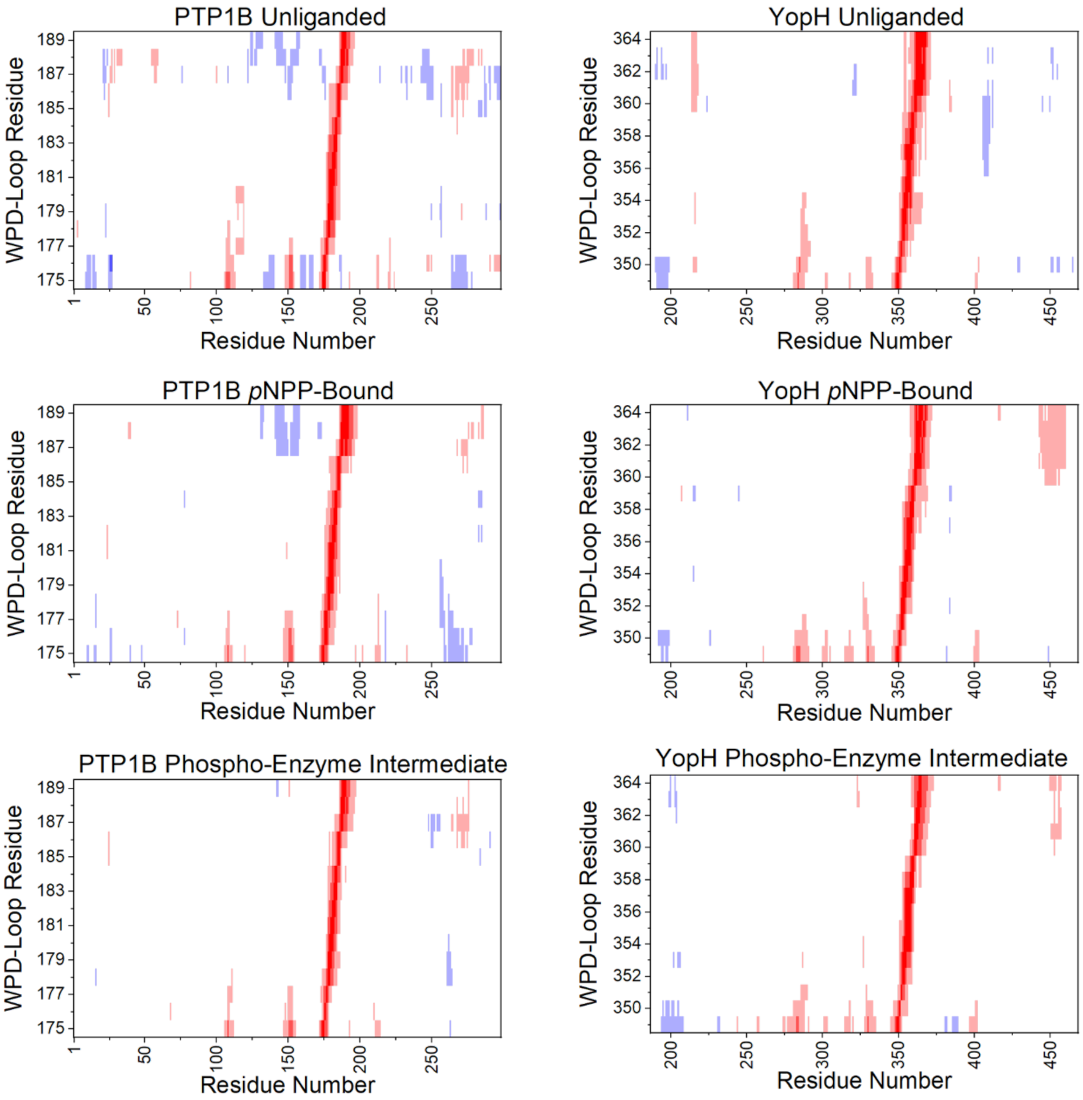

Figure S14. Truncated dynamic cross correlation matrices (DCCMs) obtained from our PT-MetaD-WTE simulations of PTP1B and YopH in their unliganded, *p*NPP-bound and phospho-enzyme intermediate states. Complete DCCMs shown in **Figure S13** are truncated on their y-axis to show only the WPD-loop residues against all other residues (on the x-axis). The color scaling of the correlation values is the same as in **Figure S13**. A value of +1 (red) indicates perfectly correlated motion, whilst -1 (blue) means perfectly anti-correlated motion.

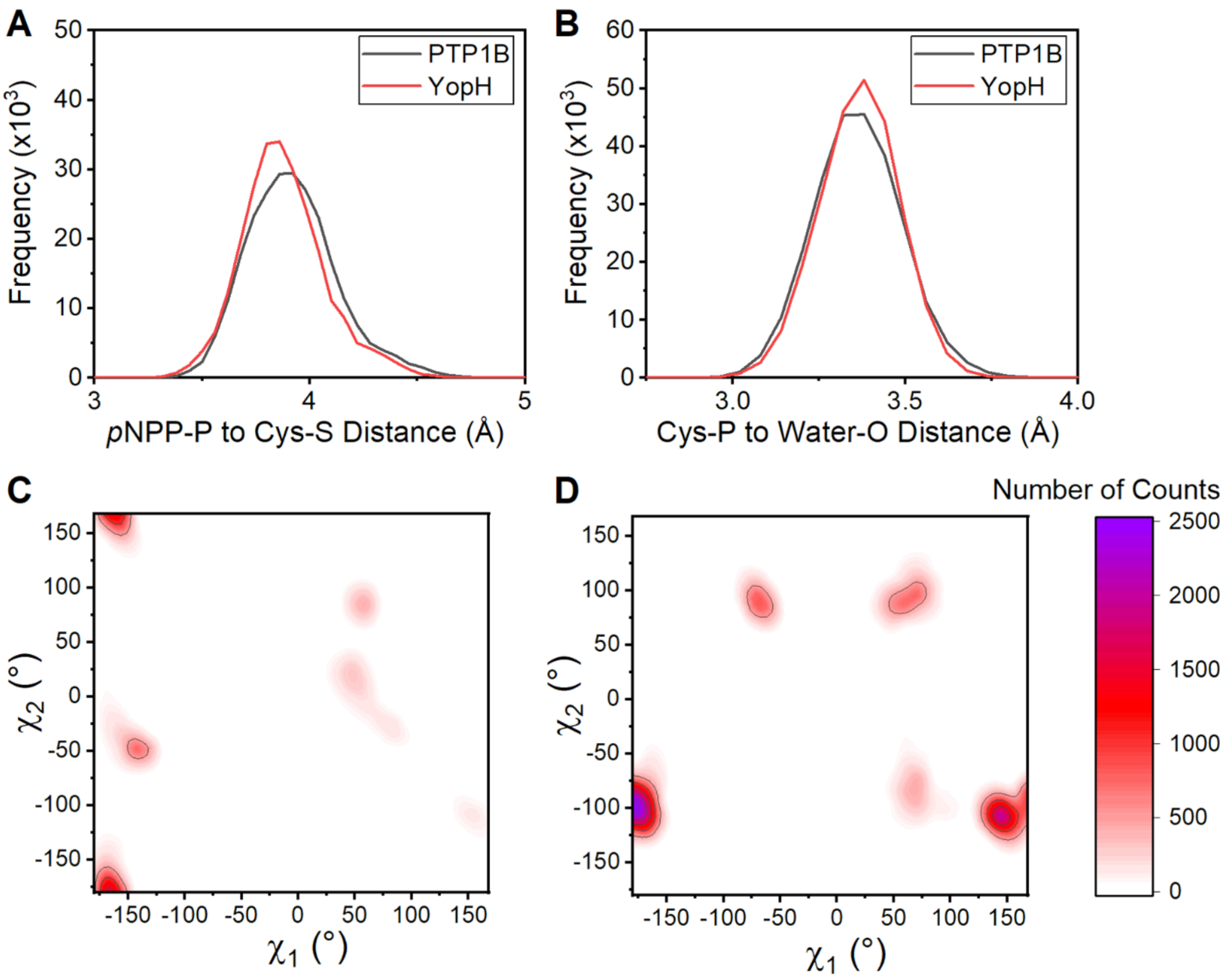

Figure S15. (**A**) Histograms (bin range 3-5 Å and bin size 0.05 Å) of the distance between the phosphorous atom of the substrate *p*NPP to the sulfur atom of the nucleophilic cysteine on the P-loop for both PTP1B and YopH, obtained from conventional MD simulations of the first chemical step. Note that a one sided harmonic restraint (often referred to as a "wall potential") was applied in both cases to maintain coordination of the substrate throughout the simulation (see the **Methodology** and **Table S6**). (**B**) Histograms (bin range 2.5-4 Å and bin size 0.05 Å) of the distance between the closest water molecule (as measured by its oxygen atom) to the phosphorus atom on the phospho-enzyme intermediate obtained from conventional MD simulations of the second chemical step. (**C**, **D**) 2D-histograms (bin range −180° to +180°, bin size 12°) of the

catalytic aspartic acid's side chain dihedral from conventional MD simulations of the first chemical step for both (**C**) PTP1B and (**D**) YopH. χ_1 is defined as the dihedral of atoms: N, C_α, C_β and C_γ and χ_2 is defined as the dihedrals of atoms C_α, C_β, C_γ and $O_{\delta1}$. In all cases, histograms of the cleavage (*i.e.*, *p*NPP-bound, panels **A**, **C** and **D**) and hydrolysis steps (*i.e.*, the phospho-enzyme intermediate, panel **B**) for both PTP1B and YopH were generated from 25x200 ns production MD simulations, with all simulations starting from the closed (catalytically competent) state.

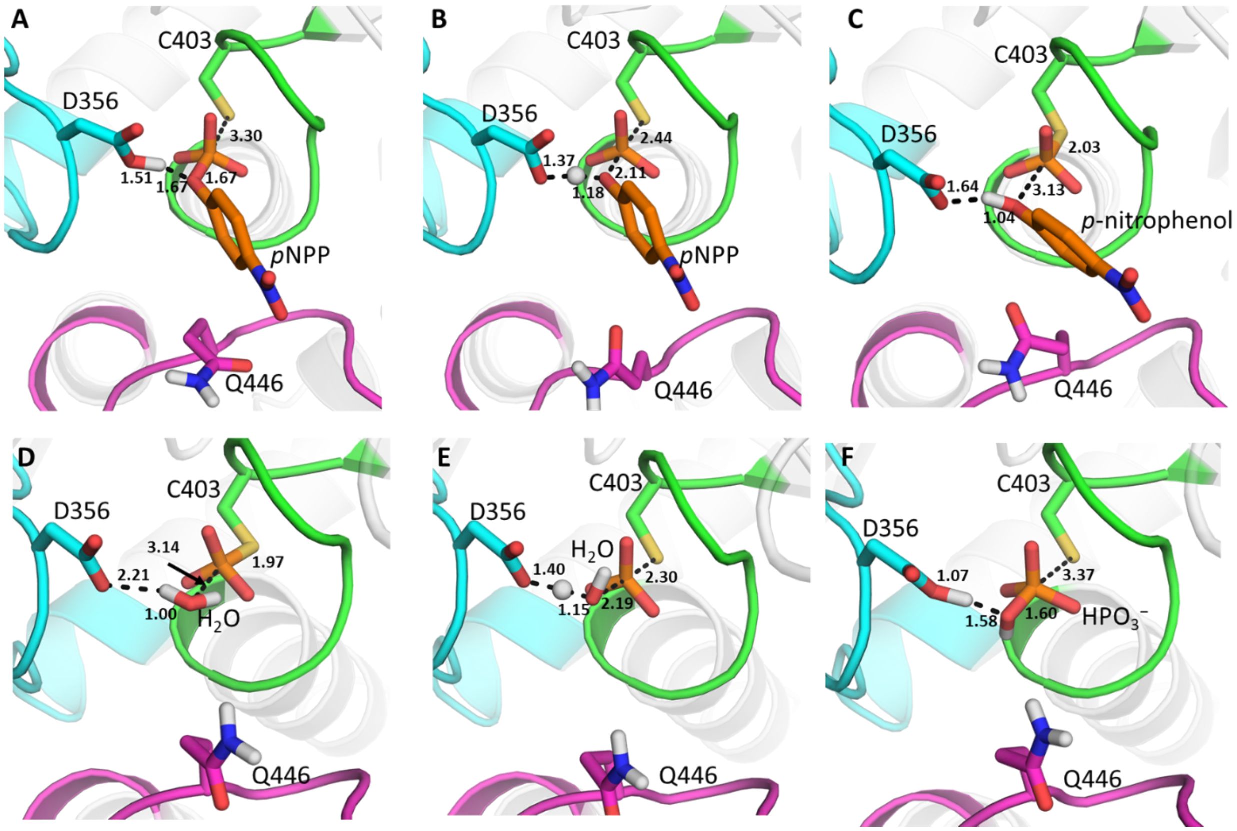

Figure S16. Representative structures of (**A**) the Michaelis complex, (**B**) the transition state for the cleavage step, (**C**, **D**) the phospho-enzyme intermediate, (**E**) the transition state for the hydrolysis step, and (**F**) the final product complex, for the YopH-catalyzed hydrolysis of *p*NPP. Note that while states **C** and **D** are essentially identical, we modelled the two reaction steps (**Figure 1**) as discrete processes, using separate starting structures with appropriate transition state analogues bound as described in the main text and the **Supplementary Methodology** section. The structures shown here are the centroids of the top ranked cluster obtained from RMSD clustering of 30 individual EVB trajectories of each stationary or saddle point, performed as described in the **Supplementary Methodology**. The equivalent figure for PTP1B is provided in the main text (See **Figure 9**).

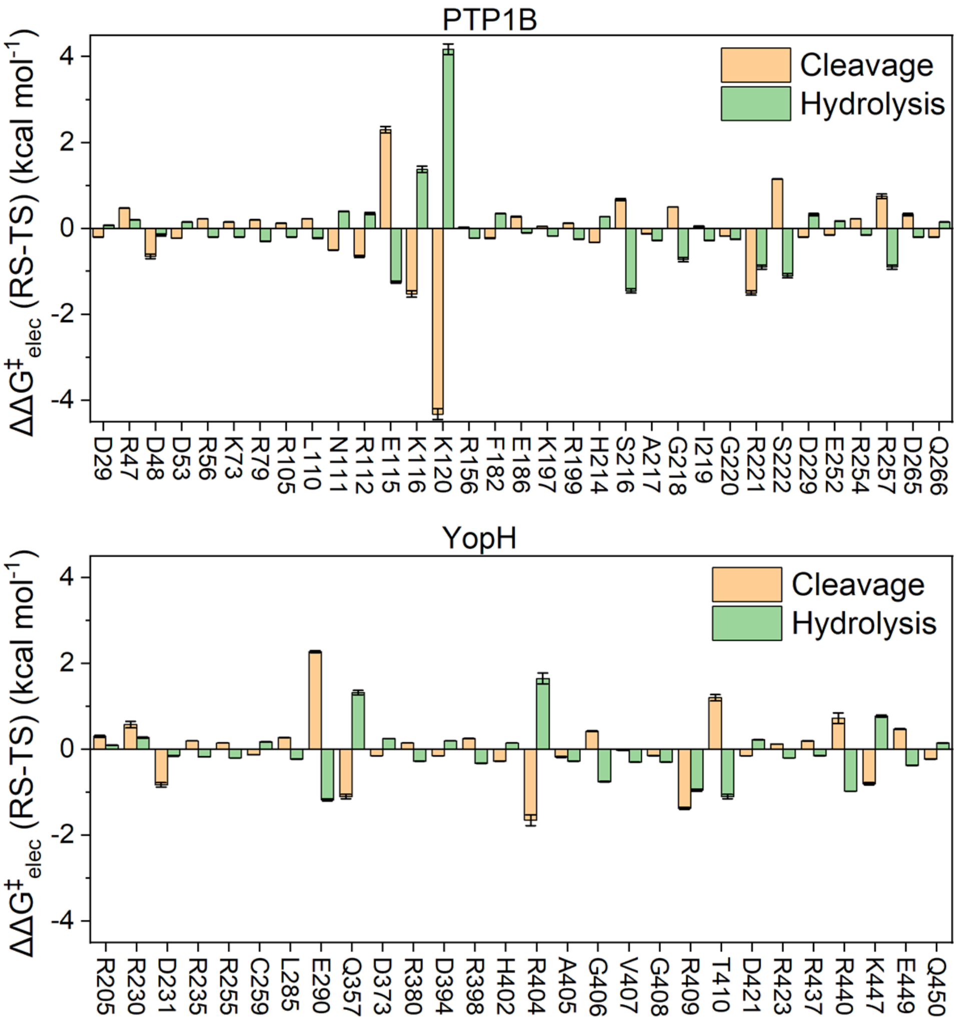

Figure S17. Electrostatic contributions of selected amino acids ($\Delta\Delta G^{\ddagger}_{elec}$, kcal mol^{-1}) to the calculated activation free energies for the cleavage and hydrolysis steps catalyzed by PTP1B and YopH. Note that all electrostatic contributions were scaled assuming an internal dielectric constant of 4.[80,81] In addition, amino acid side chains that are directly participating in the transition state (from the catalytic cysteine and aspartic acid) are not shown here. Note that reacting atoms (including catalytic residues) are not shown in this plot.

State 1

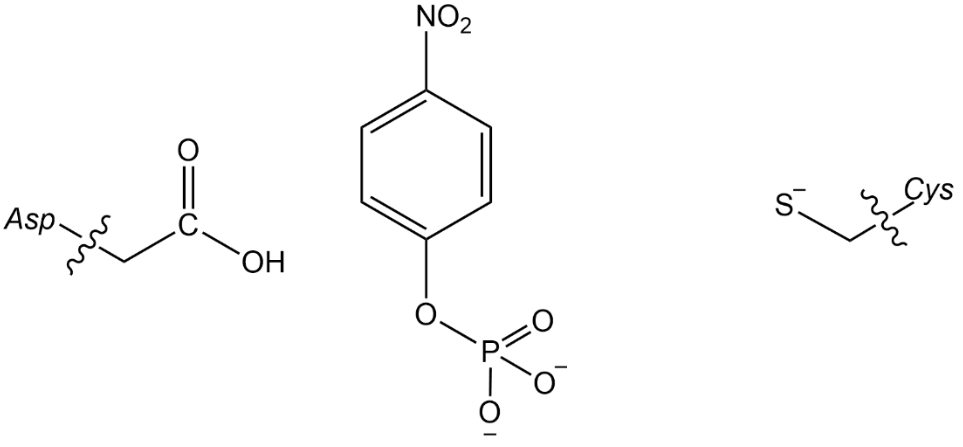

State 2

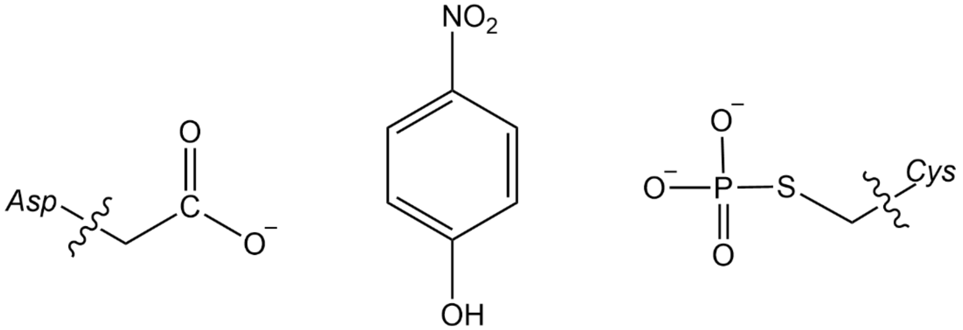

Figure S18. Valence bond states used to describe the cleavage reaction (*i.e.* first chemical step) catalysed by PTP1B and YopH. Note that for the non-enzymatic reaction, we use a truncated system whereby only the side chains of the reacting amino acid residues are used, as described in the **Methodology** section. Atom numbering for the valence bond states is depicted in **Figure S23**.

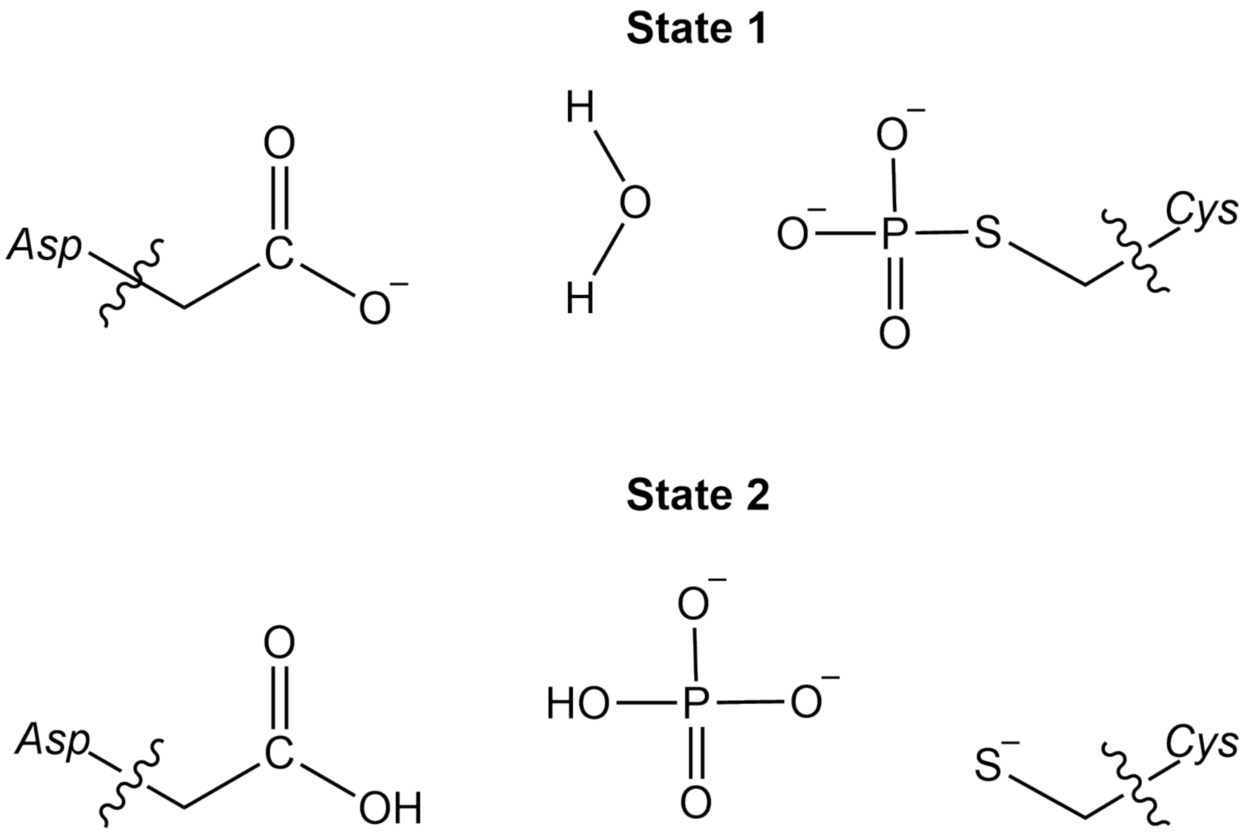

Figure S19. Valence bond states used to describe the hydrolysis reaction (*i.e.*, second chemical step) catalysed by PTP1B and YopH. Note that for the non-enzymatic reaction, we use a truncated system whereby only the side chains of the reacting amino acid residues are used, as described in the **Methodology** section. Atom numbering for the valence bond states is depicted in **Figure S24**.

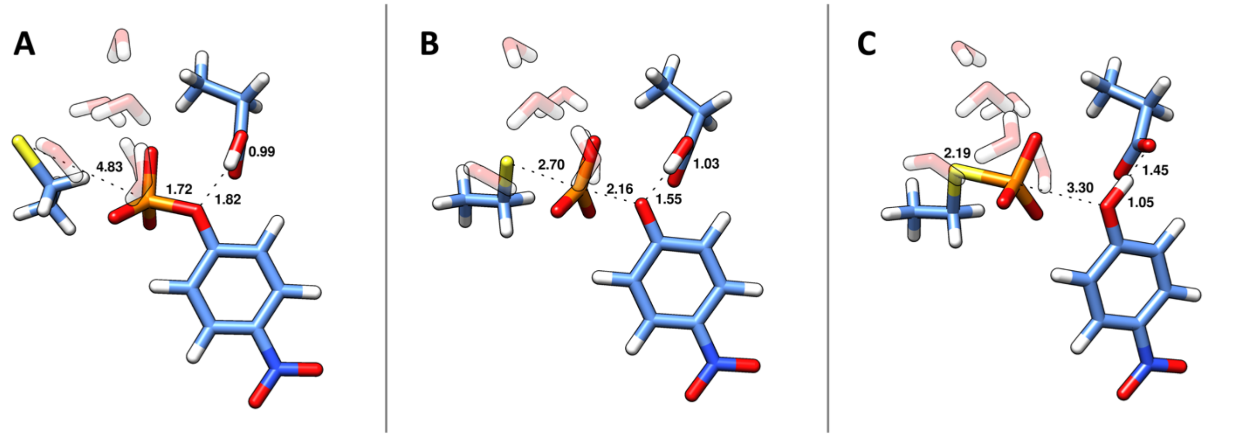

Figure S20. Key reacting distances at the (**A**) reactant, (**B**) transition and (**C**) product states obtained from DFT optimization of the non-enzymatic nucleophilic attack of ethanethiolate on *p*NPP in the presence of propionic acid. Calculations were performed at the SMD/M062X/6-31+G(d,p) level of theory. The corresponding raw data for this figure is shown in **Table S13** and the corresponding Cartesian coordinates are shown in **Section S7**.

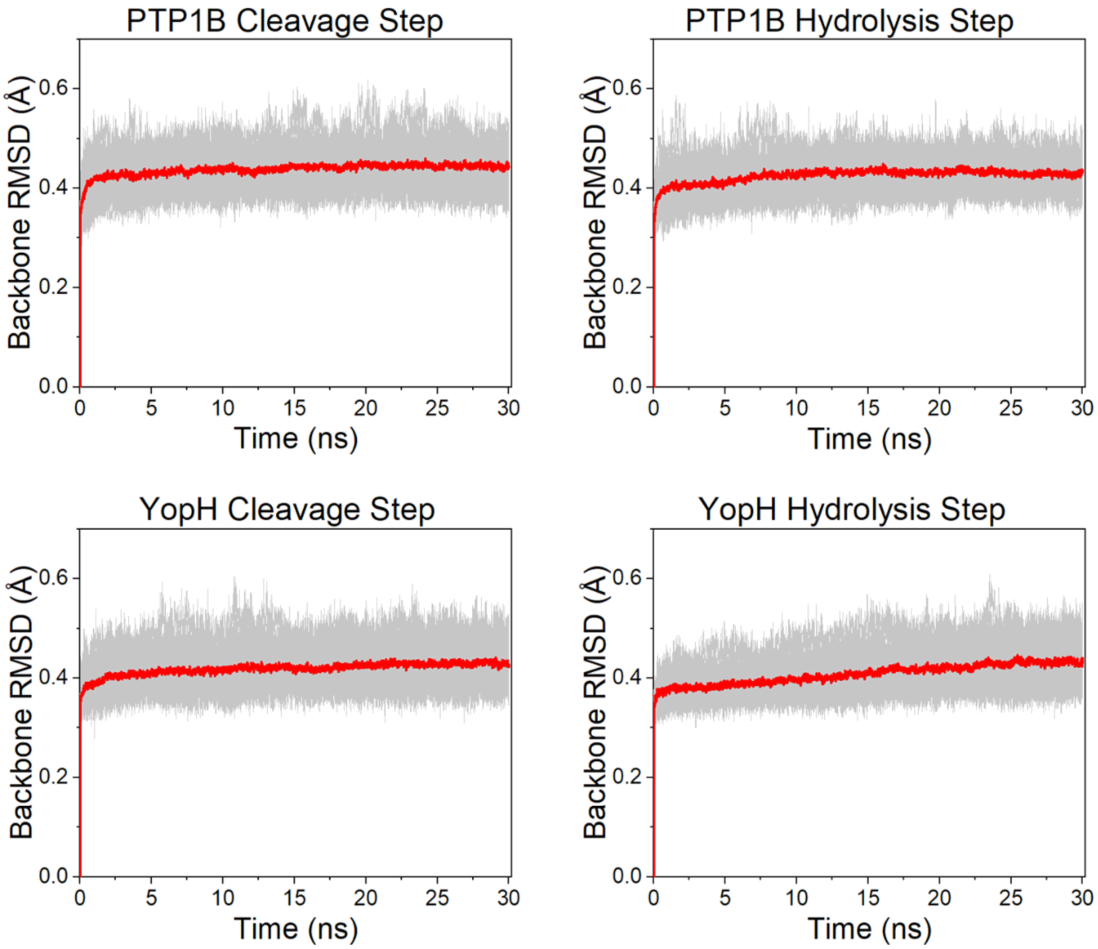

Figure S21. The root mean square deviations (RMSD, Å) of all backbone atoms during our EVB equilibration MD simulations. Equilibration simulations were performed at the approximate EVB transition states ($\lambda = 0.5$) for both PTPs and catalytic steps. Shown here are both PTP1B and YopH during the first (cleavage) and second (hydrolysis) steps of the catalytic reaction. Data was collected every 10 ps from 30 replicas each of length 30 ns. The grey lines show the 30 individual runs, whilst the red line shows a rolling average RMSD from all 30 replicas.

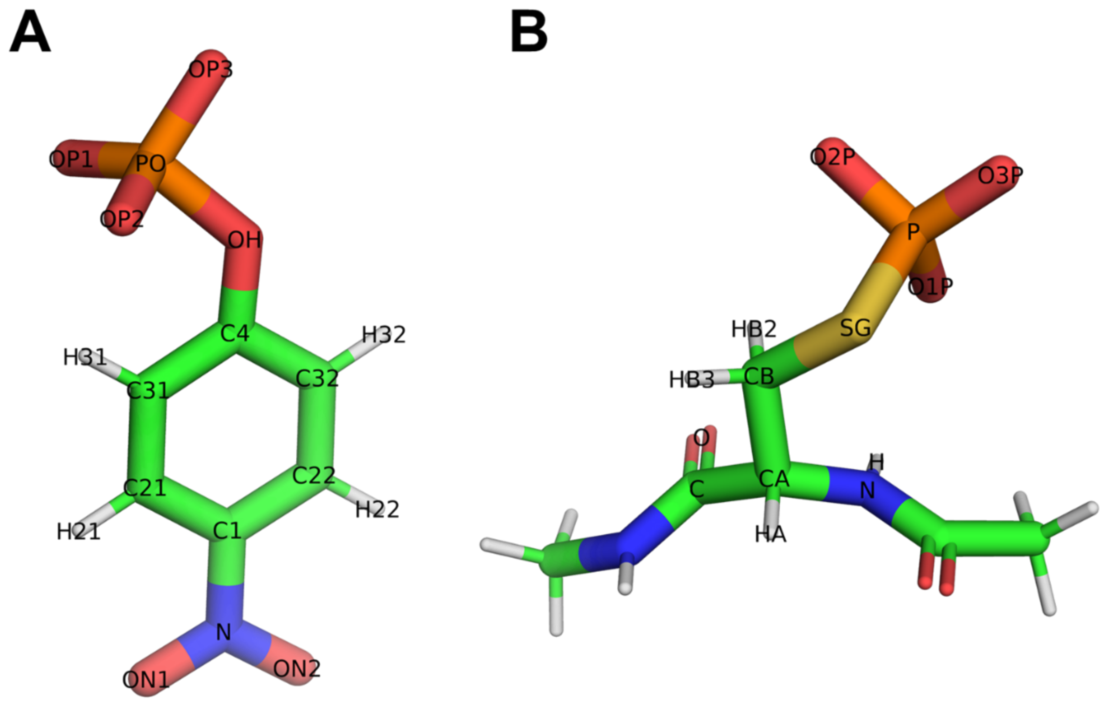

Figure S22. Labelling of the atom types of the substrate *p*-nitrophenyl phosphate (*p*NPP, **A**) and the phosphorylated cysteine residue in the phospho-enzyme intermediate (CSP, **B**). In panel **B**, capping groups to the main chain are shown only for completeness, and only the atoms corresponding to the CSP residue in our simulations are labelled. The corresponding force field parameters used in our simulations are provided in **Table S4** (*p*NPP) and **Table S5** (CSP).

S4. Supplementary Tables

Table S1. All X-ray crystal structures obtained from the Protein Data Bank (PDB)[1] for the structural analysis performed in this work.

PTP1B Structures	YopH Structures	YopH-PTP1B Chimeras[a]
1aax,[82] 1bzc,[83] 1bzh,[83] 1bzj,[83] 1c83,[84] 1c84,[84] 1c85,[84] 1c86,[85] 1c87,[85] 1ecv,[84] 1een,[86] 1eeo,[86] 1g1f,[87] 1g1g,[87] 1g1h,[87] 1g7f,[88] 1g7g,[88] 1gfy,[89] 1i57,[90] 1jf7,[91] 1l8g,[92] 1nl9,[93] 1nny,[93] 1no6,[93] 1nwe,[94] 1nwl,[94] 1nz7,[95] 1oem,[96] 1oeo,[96] 1ony,[97] 1onz,[97] 1ph0,[98] 1pty,[82] 1pxh,[99] 1pyn,[100] 1q1m,[101] 1q6j,[102] 1q6m,[102] 1q6n,[102] 1q6p,[102] 1q6s,[102] 1q6t,[102] 1qxk,[69] 1sug,[103] 1t48,[70] 1t49,[70] 1t4j,[70] 1xbo,[104] 2azr,[105] 2b07,[105] 2bge,[106] 2f6f,[107] 2f6t,[108] 2f6v,[108] 2f6w,[108] 2f6y,[108] 2f6z,[108] 2f70,[108] 2f71,[108] 2fjn,[109] 2h4g,[110] 2h4k,[110] 2hb1,[110] 2nt7,[111] 2nta[111] 2qbp,[112] 2qbq,[112] 2qbr,[112] 2qbs,[112] 2veu,[113] 2vev,[113] 2vew,[113] 2vex,[113] 2vey,[113] 2zmm,[114] 2zn7,[114] 3a5j,[b] 3a5k,[b] 3d9c,[115] 3eax,[116] 3eb1,[116] 3eu0,[116] 3i7z,[9] 3i80,[9] 3qkp,[117] 3qkq,[117] 3sme,[118] 3zv2,[119] 4bjo,[120] 4i8n,[121] 4qah,[122] 4qap[122] 4qbe,[122] 4qbw,[122] 4zrt,[123] 5k9w,[124] 5ka0,[124] 5ka1,[124] 5ka2,[124] 5ka3,[124] 5ka4,[124] 5ka7,[124] 5ka8,[124] 5ka9[124] 5kaa,[124] 5kab,[124] 5kad,[124] 5qde,[8] 5qdf,[8] 5qdg,[8] 5qdh,[8] 5qdi,[8] 5qdj,[8] 5qdk,[8] 5qdl,[8] 5qdm,[8] 5qdn,[8] 5qdo,[8] 5qdp,[8] 5qdq,[8] 5qdr,[8] 5qds,[8] 5qdt,[8] 5qdu,[8] 5qdv,[8] 5qdw,[8] 5qdx,[8] 5qdy,[8] 5qdz,[8] 5qe0,[8] 5qe1,[8] 5qe2,[8] 5qe3,[8] 5qe4,[8] 5qe5,[8] 5qe6,[8] 5qe7[8] 5qe8,[8] 5qe9,[8] 5qea,[8] 5qeb,[8] 5qec,[8] 5qed,[8] 5qee,[8] 5qef,[8] 5qeg,[8] 5qeh,[8] 5qei,[8] 5qej,[8] 5qek,[8] 5qel,[8] 5qem,[8] 5qen,[8] 5qeo,[8] 5qep,[8] 5qeq,[8] 5qer,[8] 5qes,[8] 5qet,[8] 5qeu,[8] 5qev,[8] 5qew,[8] 5qex,[8] 5qey[8] 5qez,[8] 5qf0,[8] 5qf1,[8] 5qf2,[8] 5qf3,[8] 5qf4,[8] 5qf5,[8] 5qf6,[8] 5qf7,[8] 5qf8,[8] 5qf9,[8] 5qfa,[8] 5qfb,[8] 5qfc,[8] 5qfd,[8] 5qfe,[8] 5qff,[8] 5qfg,[8] 5qfh,[8] 5qfi,[8] 5qfj,[8] 5qfk,[8] 5qfl,[8] 5qfm,[8] 5qfn,[8] 5qfo,[8] 5qfp,[8] 5qfq,[8] 5qfr,[8] 5qfs,[8] 5qft,[8] 5qfu,[8] 5qfv,[8] 5qfw,[8] 5qfx,[8] 5qfy,[8] 5qfz,[8] 5qg0,[8] 5qg1,[8] 5qg2,[8] 5qg3,[8] 5qg4,[8] 5qg5,[8] 5qg6,[8] 5qg7,[8] 5qg8,[8] 5qg9,[8] 5qga,[8] 5qgb,[8] 5qgc,[8] 5qgd,[8] 5qge,[8] 5qgf,[8] 5t19,[125] 6b8e,[8] 6b8t,[8] 6b8x,[8] 6b8z,[8] 6b90,[8] 6b95,[8] 6bai,[8] 6cwu,[75] 6cwv.[75]	1lyv,[b] 1qz0,[5] 1xxp,[126] 1xxv,[126] 2i42,[7] 3blt,[127] 3blu,[127] 3bm8,[127] 3f99,[128] 3f9a,[128] 3f9b,[128] 3u96,[129] 4yaa,[130] 4z6b,[130] 4zi4,[130] 4zn5.[130]	6dr1,[4] 6dr7,[4] 6dt6.[4]

[a] The YopH-PTP1B chimeras consist of the YopH enzyme with part of the PTP1B WPD-loop transposed into the sequence.[4] [b] No publication is associated with this structure.

Table S2. X-ray crystal structures used for simulations of PTP1B and YopH.[a]

Wild-Type PTP1B		
**Simulation**	**PDB ID**	**Modifications Required before simulation**
Unliganded-Enzyme Closed	6B90[8]	None (both open and closed WPD-loop conformations are provided in this PDB).
Unliganded-Enzyme Open	6B90[8]	None (both open and closed WPD-loop conformations are provided in this PDB).
*p*NPP-Bound Closed[b]	3I7Z[9]	Inhibitor modified to *p*NPP, geometry optimized.
*p*NPP-Bound Open[b]	6B90[8]	Manual placement of *p*NPP into the active site based on position of *p*NPP in the ligand-bound closed state (see previous row).
CSP State Closed[c]	3I80[9]	Bound vanadate ion modified into a phosphate group, geometry optimized.
CSP State Open[c]	6B90[8]	Constructed based on CSP-bound closed state (see previous row).
**Wild-Type YopH**		
**Simulation**	**PDB ID**	**Modifications Required before simulation**
Unliganded-Enzyme Closed	2I42[7]	Bound VO_4 ion removed.
Unliganded-Enzyme Open	1YPT[6]	None.
*p*NPP-Bound Closed[b]	1QZ0[5]	Inhibitor modified to become *p*NPP, geometry optimized.
*p*NPP-Bound Open[b]	1YPT[6]	Manual placement of *p*NPP into the active site based on position of *p*NPP in the ligand-bound closed state (see previous row).
CSP State Closed[c]	2I42[7]	Bound vanadate ion modified into a phosphate group, geometry optimized.
CSP State Open[c]	1YPT[6]	Constructed based on CSP-bound closed state (see previous row).

[a] "Open" and "Closed" refer to the conformations of the WPD-loop in each crystal structure. Shown here are also any modifications made to each structure prior to simulations, if relevant. [b] *p*NPP-bound denotes the *p*NPP-bound Michaelis complex. [c] CSP denotes the phospho-enzyme intermediate, with the catalytic cysteine phosphorylated.

Table S3. Non-standard protonation states and histidine tautomerization states used in all simulations.[a]

System	Non-Standard Protonation States			HIE Tautomerisation State[b]
	**Catalytic Aspartic acid**	**Catalytic Cysteine**	**Extra Residues**	
**WT PTP1B**				
Unliganded-Enzyme	Deprotonated	Deprotonated	102GlH[e]	25, 54, 60, 94, 173, 175, 208, 296
*p*NPP-Bound[c]	Protonated	Deprotonated	102GlH[e]	(same as above)
CSP-State[d]	Deprotonated	N/A	102GlH[e]	(same as above)
**WT YopH**				
Unliganded-Enzyme	Deprotonated	Deprotonated	None	270, 350
*p*NPP-Bound[c]	Protonated	Deprotonated	None	(same as above)
CSP-State[d]	Deprotonated	N/A	None	(same as above)

[a] For a description of how these states were determined, see the main text. [b] HIE corresponds to a neutral histidine residue which is protonated on its $N_{\epsilon 2}$ nitrogen atom. All other histidine residues were simulated as neutral and protonated on their $N_{\delta 1}$ nitrogen (HID). [c] *p*NPP-Bound denotes the *p*NPP-bound Michaelis complex. [d] CSP-state denotes the phospho-enzyme intermediate, with the catalytic cysteine phosphorylated. [e] GLH denotes a protonated glutamic acid side chain.

Table S4. The GAFF2 force field[14] parameters used to describe the substrate *p*-nitrophenyl phosphate (*p*NPP).

Atom Name	Atom Type	Partial Charge	Atom Name	Atom Type	Partial Charge
N	no	0.796	OH	os	-0.712
ON1	o	-0.553	H21	ha	0.169
ON2	o	-0.553	H22	ha	0.169
C1	ca	-0.134	H31	ha	0.186
C21	ca	-0.124	H32	ha	0.186
C22	ca	-0.124	PO	p5	1.386
C31	ca	-0.442	OP1	o	-0.905
C32	ca	-0.442	OP2	o	-0.905
C4	ca	0.905	OP3	o	-0.905

[a] Parameterization was performed as described in the main text. Labelling of atom types can be found in **Figure S22**.

Table S5. The ff14SB force field[16] parameters used to describe the phosphorylated cysteine residue (CSP) at the phospho-enzyme intermediate.[a]

Atom Name	Atom Type	Partial Charge	Atom Name	Atom Type	Partial Charge
N	N	-0.416	O1P	O2	-0.900
CA	CX	-0.035	O2P	O2	-0.900
C	C	0.597	O3P	O2	-0.900
O	O	-0.568	H	H	0.272
CB	CT	0.045	HA	H1	0.051
SG	S	-0.562	HB2	H1	0.037
P	P	1.242	HB3	H1	0.037

[a] Parameterization was performed as described in the main text. Any missing force field terms were added by analogy using the GAFF2 force field.[14] Labelling of atom types can be found in **Figure S22**.

Table S6. Restraints used to stabilize the *p*NPP substrate (residue ID: PND) in a catalytically competent position during the conventional MD and PT-MetaD-WTE simulations with *p*NPP-bound to both PTP1B and YopH.[a]

**Distance Restraints**							
**Restraint Index**	**Atom IDs**	**Distances (Å)**				**Restraints (kcal mol^{-1} $Å^{-2}$)**	
		r^1	r^2	r^3	r^4	rk^2	rk^3
Distance1	PND@PO<br>CYM@SG	0.0	0.0	3.8	10.0	0.0	10.0
Distance2	PND@OP1<br>RES1[b]@H	0.0	0.0	2.5	10.0	0.0	10.0
Distance3	PND@OP2<br>RES2[c]@H	0.0	0.0	2.5	10.0	0.0	10.0
Distance4	PND@OP3<br>RES3[d]@H	0.0	0.0	2.5	10.0	0.0	10.0
**Angle Restraints**							
**Restraint Index**	**Atom IDs**	**Angles (°)**				**Restraints (kcal mol^{-1} $°^{-2}$)**	
		r^1	r^2	r^3	r^4	rk^2	rk^3
Angle1	PND@OH<br>PND@PO<br>CYM@SG	-10	140	180	350	10.0	10.0

[a] The values r^1 through to r^4, are the four positions used to define the restraints, and are presented in "Å" or "°" respectively. The force constants rk^2 and rk^3 are for the left and right parabolas respectively and are shown in kcal mol^{-1} $Å^{-2}$. Note that the four distance restraints are "half-sided", meaning no restraint is felt unless r^3 is exceeded. Note also that no restraints are placed between *p*NPP and the WPD-loop. [b] Residue in PTP1B is: S216, residue in YopH is R404. [c] Residue in PTP1B is: G220, residue in YopH is G408. [d] Residue in PTP1B is: I219, residue in YopH is V407.

Table S7. Collective variables (CVs) used for our PT-MetaD-WTE simulations of PTP1B.[a]

**PTP1B**								
**CV Index**	**CV Type**	**Residue selection 1**	**Residue selection 2**	**Grid Min and Max**	**Sigma**	**Upper Wall Position**[a]	**Gaussian Height**	**Bias Factor**
CV0[b]	Potential energy	N/A	N/A	−160000.0 to −40000.0 kcal mol^{-1}	100.0 kcal mol^{-1}	None.	0.6 kcal mol^{-1}	60
CV1	Inter Distance RMSD	Cα of residues 175-190	Cα of residues 214-220	0 to 10 Å	0.1 Å	6.9 Å	0.2 kcal mol^{-1}	12
CV2	Center of Mass Distance	Heavy atoms of residues 181-182	Backbone heavy atoms of residues 214-221	2 to 30 Å	0.3 Å	23 Å	0.2 kcal mol^{-1}	12
CV3	Center of Mass Distance	Heavy atoms of residues 184-185	Backbone heavy atoms of residues 214-221	6 to 30 Å	0.3 Å	24 Å	0.2 kcal mol^{-1}	12

[a] Upper wall potentials have a restraint size of 50 kcal mol^{-1} $Å^{-2}$ for deviations beyond the wall. [b] Note that CV0 was held constant (after an initial 10 ns long simulation) during the simulations in order to run our PT-MetaD simulations in the well-tempered ensemble (WTE), see **Methodology** and **Supplementary Methodology**. Note also that the same CVs (and CV parameters) were used for all three states simulated. A graphical representation of the CVs used is provided in **Figure S5**.

Table S8. Collective variables (CVs) used for our PT-MetaD-WTE simulations of YopH.[a]

**YopH**								
**CV Index**	**CV Type**	**Residue selection 1**	**Residue selection 2**	**Grid Min and Max**	**Sigma**	**Upper Wall Position**[a]	**Gaussian Height**	**Bias Factor**
CV0[b]	Potential energy	N/A	N/A	−160000.0 to −40000.0 kcal mol^{-1}	110.0 kcal mol^{-1}	None	0.6 kcal mol^{-1}	60
CV1	Inter Distance RMSD	C_α of residues 351-365	C_α of residues 403-409	0 to 10 Å	0.1 Å	8 Å	0.2 kcal mol^{-1}	12
CV2	Center of Mass Distance	Heavy atoms of residues 356-357	Backbone heavy atoms of residue 403-410	2 to 30 Å	0.3 Å	25 Å	0.2 kcal mol^{-1}	12
CV3	Center of Mass Distance	Heavy atoms of residues 359-360	Backbone heavy atoms of residue 403-410	4 to 30 Å	0.3 Å	25 Å	0.2 kcal mol^{-1}	12

[a] Upper wall potentials have a restraint size of 50 kcal mol^{-1} $Å^{-2}$ for deviations beyond the wall. [b] Note that CV0 was held constant (after an initial 10 ns long simulation) during the simulations in order to run our PT-MetaD simulations in the well-tempered ensemble (WTE), see **Methodology** and **Supplementary Methodology**. Note also that the same CVs (and CV parameters) were used for all three states simulated. A graphical representation of the CVs used is provided in **Figure S5**.

Table S9. Non-WPD-loop or P-loop single point mutations available from the literature that are known to alter PTP1B k_{cat} or K_m by 50%.[a]

**Mutations**	**k_{cat} (s^{-1})**	**K_m (μM)**	**Mutant k_{cat} / WT k_{cat}**	**Mutant K_m / WT K_m**
**Set 1:**[75]				
WT	9.2	282	N/A	N/A
R254A	0.4	2696	**0.04**	**9.56**
G259S	4.3	587	**0.47**	**2.08**
R56G	1.9	2930	**0.21**	**10.39**
**Set 2:**[8]				
WT	5.22	1.92	N/A	N/A
K197A	2.22	2.31	**0.43**	1.20
Y152G	1.61	3.51	**0.31**	**1.83**
Y153G	0.81	1.32	**0.16**	0.69
**Set 3:**[74]				
WT	56	0.57	N/A	N/A
Y153A	49	1.1	0.88	**1.93**
L232A	26	1.0	**0.46**	**1.75**
E276A	49	1.1	0.88	**1.93**
M282A	60	0.9	1.07	**1.58**
E297A	43	1.1	0.77	**1.93**
**Set 4:**[124]				
WT	4.4	5.0	N/A	N/A
L192A	0.5	0.46	**0.11**	**0.09**
N193A	2.5	1.8	0.57	**0.36**
**Set 5:**[89]				
WT	38.5	0.77	N/A	N/A
G259Q	16.3	3.02	**0.42**	**3.92**
**Set 6:**[131]				
WT	100%[b]	Not available[b]	N/A	N/A
A69V	<50%[b]	Not available[b]	**<0.5**	N/A

[a] Mutations which meet this threshold (> |50%|) change in k_{cat} or K_m are marked in bold. The impact of each mutation is assessed against the WT kinetics data from the same study. In total, eleven different positions were found. [b] PTP1B activity assays were performed in lysates,[131] meaning only a relative activity value is available, see ref. 131 for further experimental details.

Table S10. Calculated distances at the Michaelis complexes (MC), transition states (TS) and product states (PS) obtained from out EVB simulations. Shown are also the average numbers of water molecules within 4 Å of the reacting atoms.[a]

Cleavage						
**System**		**S_{Cys}-P**	**P-O_{pNPP}**	**O_{pNPP}-H**	**H-O_{Asp}**	**No. of waters**
**Non-enzymatic**	**RS**	4.33 ± 0.05	1.62 ± 0.01	2.38 ± 0.07	1.14 ± 0.02	12.4 ± 0.3
	**TS1**	2.69 ± 0.04	2.05 ± 0.03	1.20 ± 0.01	1.36 ± 0.01	12.5 ± 0.3
	**IS**	2.13 ± 0.02	3.10 ± 0.03	1.04 ± 0.01	3.45 ± 0.08	12.3 ± 0.4
**PTP1B**	**MC**	3.38 ± 0.03	1.67 ± 0.01	1.52 ± 0.01	1.09 ± 0.01	0.2 ± 0.1
	**TS1**	2.49 ± 0.02	2.12 ± 0.02	1.19 ± 0.01	1.37 ± 0.01	0.5 ± 0.1
	**IS**	2.04 ± 0.01	3.34 ± 0.05	1.03 ± 0.01	1.66 ± 0.02	1.4 ± 0.1
**YopH**	**MC**	3.30 ± 0.03	1.67 ± 0.01	1.51 ± 0.01	1.09 ± 0.01	0.7 ± 0.1
	**TS1**	2.44 ± 0.02	2.11 ± 0.02	1.18 ± 0.01	1.37 ± 0.01	0.8 ± 0.1
	**IS**	2.03 ± 0.01	3.13 ± 0.05	1.04 ± 0.01	1.64 ± 0.02	1.3 ± 0.2
**Hydrolysis**						
		**S_{Cys}-P**	**P-O_{H2O}**	**O_{H2O}-H**	**H-O_{Asp}**	**No. of waters**
**Non-enzymatic**	**IS**	2.12 ± 0.02	3.28 ± 0.04	1.01 ± 0.01	3.16 ± 0.04	13.3 ± 0.2
	**TS2**	2.54 ± 0.04	2.02 ± 0.03	1.18 ± 0.01	1.38 ± 0.01	13.9 ± 0.3
	**PS**	4.64 ± 0.06	1.57 ± 0.01	2.01 ± 0.08	1.09 ± 0.02	13.6 ± 0.3
**PTP1B**	**IS**	1.98 ± 0.01	3.09 ± 0.04	1.00 ± 0.01	2.35 ± 0.14	2.4 ± 0.1
	**TS2**	2.33 ± 0.02	2.16 ± 0.02	1.16 ± 0.01	1.41 ± 0.01	2.3 ± 0.1
	**PS**	3.43 ± 0.03	1.60 ± 0.01	1.59 ± 0.01	1.06 ± 0.01	2.6 ± 0.1
**YopH**	**IS**	1.97 ± 0.01	3.14 ± 0.03	1.00 ± 0.01	2.21 ± 0.15	2.7 ± 0.1
	**TS2**	2.30 ± 0.02	2.19 ± 0.02	1.15 ± 0.01	1.40 ± 0.01	3.0 ± 0.1
	**PS**	3.37 ± 0.03	1.60 ± 0.01	1.58 ± 0.01	1.07 ± 0.01	3.6 ± 0.1

[a] RS/MC, TS1, IS, TS2 and PS correspond to the reactant state/Michaelis complex, transition state for the cleavage, phospho-enzyme intermediate, transition state for the hydrolysis and product state, respectively. Note that while the IS obtained at the endpoint of the cleavage and starting point of the hydrolysis reactions are chemically identical, we modelled the two stages of the reactions using separate crystal structures with appropriate transition state analogues for each reaction step bound to the active site, as described in the main text. S_{Cys}-P denotes the distance between the cysteine side chain and the phosphorus atom of the phosphate group, P-O_{pNPP} denotes the distance between the

phosphorus atom and the leaving group oxygen in the cleavage step, P-O_{H2O} denotes the distance between the phosphorus atom and the nucleophilic water molecule in the hydrolysis step, $O_{p\mathrm{NPP}}$-H denotes the distance between the leaving group oxygen and the proton being transferred from the aspartic acid side chain in the cleavage step, O_{H2O}-H denotes the distance between the nucleophilic water molecule and the proton being transferred back to the aspartic acid side chain in the hydrolysis step, and H-O_{Asp} denotes the distance between the proton and the relevant oxygen atom of the aspartic acid side chain. All distances are shown in Å. Data is presented as average values and standard error of the mean over 30 individual EVB trajectories per system / reaction step.

Table S11. Electrostatic contributions of individual amino acids ($\Delta\Delta G^{\ddagger}_{elec}$, kcal mol^{-1}) to the calculated activation free energies for the cleavage and hydrolysis steps catalyzed by PTP1B and YopH.[a]

Amino Acid	PTP1B		Amino Acid	YopH	
	Cleavage	Hydrolysis		Cleavage	Hydrolysis
D29	-0.20 ± 0.01	0.08 ± 0.01	R205	0.30 ± 0.03	0.10 ± 0.01
R47	0.48 ± 0.01	0.20 ± 0.01	R230	0.58 ± 0.08	0.28 ± 0.01
D48	-0.65 ± 0.05	-0.15 ± 0.03	D231	-0.83 ± 0.05	-0.15 ± 0.01
D53	-0.23 ± 0.01	0.15 ± 0.01	R235	0.20 ± 0.01	-0.18 ± 0.01
R56	0.23 ± 0.01	-0.20 ± 0.01	R255	0.15 ± 0.01	-0.20 ± 0.01
K73	0.15 ± 0.01	-0.20 ± 0.01	C259	-0.13 ± 0.01	0.18 ± 0.01
R79	0.20 ± 0.01	-0.30 ± 0.01	L285	0.28 ± 0.01	-0.23 ± 0.01
R105	0.13 ± 0.01	-0.20 ± 0.01	E290	2.28 ± 0.03	-1.18 ± 0.03
L110	0.23 ± 0.01	-0.23 ± 0.01	Q357	-1.10 ± 0.05	1.33 ± 0.05
N111	-0.50 ± 0.01	0.40 ± 0.01	D373	-0.15 ± 0.01	0.25 ± 0.01
R112	-0.65 ± 0.03	0.35 ± 0.03	R380	0.15 ± 0.01	-0.28 ± 0.01
E115	2.30 ± 0.08	-1.25 ± 0.03	D394	-0.15 ± 0.01	0.20 ± 0.01
K116	-1.53 ± 0.08	1.38 ± 0.08	R398	0.25 ± 0.01	-0.33 ± 0.01
K120	-4.33 ± 0.13	4.18 ± 0.13	H402	-0.28 ± 0.01	0.15 ± 0.01
R156	0.03 ± 0.01	-0.23 ± 0.01	R404	-1.65 ± 0.13	1.65 ± 0.13
F182	-0.23 ± 0.01	0.35 ± 0.01	A405	-0.18 ± 0.01	-0.28 ± 0.01
E186	0.28 ± 0.01	-0.10 ± 0.01	G406	0.43 ± 0.01	-0.75 ± 0.01
K197	0.05 ± 0.01	-0.18 ± 0.01	V407	-0.03 ± 0.01	-0.30 ± 0.01
R199	0.13 ± 0.01	-0.25 ± 0.01	G408	-0.15 ± 0.01	-0.30 ± 0.01
H214	-0.33 ± 0.01	0.28 ± 0.01	R409	-1.38 ± 0.03	-0.95 ± 0.03
S216	0.68 ± 0.03	-1.45 ± 0.05	T410	1.20 ± 0.08	-1.10 ± 0.05
A217	-0.13 ± 0.01	-0.28 ± 0.01	D421	-0.15 ± 0.01	0.23 ± 0.01
G218	0.50 ± 0.01	-0.73 ± 0.05	R423	0.13 ± 0.01	-0.20 ± 0.01
I219	0.05 ± 0.01	-0.28 ± 0.01	R437	0.20 ± 0.01	-0.15 ± 0.01
G220	-0.18 ± 0.01	-0.25 ± 0.01	R440	0.73 ± 0.13	-0.98 ± 0.01
R221	-1.50 ± 0.05	-0.90 ± 0.05	K447	-0.80 ± 0.03	0.78 ± 0.03
S222	1.15 ± 0.01	-1.10 ± 0.05	E449	0.48 ± 0.01	-0.38 ± 0.01
D229	-0.20 ± 0.01	0.33 ± 0.03	Q450	-0.23 ± 0.01	0.15 ± 0.01
E252	-0.15 ± 0.01	0.18 ± 0.01			
R254	0.23 ± 0.01	-0.15 ± 0.01			
R257	0.75 ± 0.05	-0.90 ± 0.05			
D265	0.33 ± 0.03	-0.20 ± 0.01			
Q266	-0.20 ± 0.01	0.15 ± 0.01			

[a] Data was obtained from our calculated EVB trajectories using the linear response approximation (LRA)[132,133] and is presented as average values and standard error of the mean over 30 individual trajectories per system. This data is presented visually in **Figure S17**. All electrostatic contributions were scaled assuming an internal dielectric constant of 4 as it is common practice for a polar active site.[80,81]

Table S12. List of ionized residues and histidine protonation patterns used in our EVB simulations of PTP1B and YopH.[a]

**Type**	**Residue Number**	
	**PTP1B**	**YopH**
Asp	11, 22, 29, 48, 53, 65, 148, 229, 236, 245, 265, 289, 298	231, 300, 325, 330, 373, 394, 421, 431, 448, 452
Glu	6, 26, 76, 115, 147, 186, 200, 252, 276, 297	196, 290, 363, 377, 430, 449, 459
Lys	73, 116, 120, 150, 197, 237, 239, 247, 248, 255, 279, 292	225, 366, 447, 456
Arg	24, 45, 47, 56, 79, 105, 112, 156, 199, 221, 238, 254, 257, 268	194, 200, 205, 228, 230, 235, 255, 303, 380, 398, 404, 409, 423, 437, 440, 463
His-ε[b]	25, 54, 60, 94, 173, 175, 208, 296	270, 350
His-δ[c]	214	402

[a] All other ionizable residues not specified in the table were simulated in their neutral state during the simulations, as they fell outside the explicit solvent sphere (see the **Methodology** section). [b] His-ε corresponds to a histidine singly protonated on its $N_{\epsilon 2}$ nitrogen atom. [c] His-δ corresponds to a histidine singly protonated on its $N_{\delta 1}$ nitrogen atom.

Table S13. Key reacting distances for the non-enzymatic model reaction, based on DFT-optimized stationary points obtained in the presence of 6 explicit water molecules.[a]

State	Reactive State Distances				ΔG
	S_{Cys}-P	P-O_{pNPP}	O_{pNPP}-H	H-O_{Asp}	
**RS**	4.83	1.72	1.82	0.99	0.0
**TS**	2.70	2.16	1.55	1.03	19.1
**PS**	2.19	3.30	1.05	1.45	2.5

[a] S_{Cys}-P denotes the distance between the cysteine side chain and the phosphorus atom of the phosphate group, P-O_{pNPP} denotes the distance between the phosphorus atom and the leaving group oxygen in the cleavage step, O_{pNPP}-H denotes the distance between the leaving group oxygen and the proton being transferred from the aspartic acid side chain in the cleavage step, and H-O_{Asp} denotes the distance between the proton and the relevant oxygen atom of the aspartic acid side chain. The calculated free energies relative to the reactant state are also shown here for reference. As in our previous work with nucleophilic attack of anionic nucleophiles on related species (see *e.g.*, refs. [134,135]), it can be seen that the DFT calculations significantly underestimate the activation free energy relative to experiment (expected value of 29.4 kcal mol^{-1}, based on experimental data presented in ref. [54]). Therefore, our objective with these calculations is only to obtain meaningful transition state geometries as these have been shown to be reliable in our prior work, even when energies are significantly underestimated (see discussion in ref. [134] and references cited therein). All distances are shown in Å and all energies are shown in kcal mol^{-1}. RS, TS and PS denote the reactant, transition and product states, respectively. All geometries were optimized at the SMD/M06-2X/6-31+G(d,p) level of theory.

Table S14. The impact of the WPD-loop conformation on the predicted pK_a values of all ionizable residues in PTP1B.[a]

Residue	Native Contact (*Q*) Value												
	**1.0**	**0.99**	**0.98**	**0.97**	**0.96**	**0.95**	**0.9**	**0.85**	**0.8**	**0.7**	**0.6**	**0.5**	**0.4**
**D11**	1.9	1.9	1.9	1.9	1.8	1.9	1.8	1.9	1.9	1.9	1.8	1.9	1.8
**D22**	3.9	3.9	4.0	3.9	4.0	3.9	4.0	4.0	4.0	4.0	4.0	4.0	3.9
**D29**	3.8	3.9	4.0	4.0	4.0	3.9	3.9	3.9	3.9	3.9	3.9	3.9	3.9
**D48**	3.8	2.5	2.5	2.5	2.6	2.8	3.1	3.1	3.0	2.9	2.4	2.4	2.6
**D53**	3.1	3.0	2.8	2.6	2.6	2.7	3.0	3.1	3.1	3.2	3.2	3.0	2.9
**D63**	3.1	3.1	3.5	3.5	3.7	3.6	3.7	3.8	3.8	3.7	3.5	3.4	3.4
**D65**	2.0	2.7	2.8	2.7	2.7	2.8	2.6	2.6	2.6	2.7	2.7	2.7	2.7
**D137**	4.0	3.9	3.9	4.0	3.9	4.0	4.0	3.9	4.0	4.0	3.9	3.8	3.8
**D148**	3.1	3.5	3.2	3.3	3.3	3.3	3.3	3.3	3.5	3.5	3.5	3.2	3.3
**D181**	**4.6**	**3.8**	**3.6**	**3.6**	**3.7**	**3.6**	**3.4**	**3.1**	**3.4**	**3.0**	**3.4**	**3.5**	**3.6**
**D229**	1.9	2.0	2.1	2.1	2.0	2.1	2.1	2.1	2.0	1.8	1.9	2.0	1.9
**D236**	3.0	3.0	3.1	3.0	3.0	3.1	3.1	3.0	3.1	3.1	3.0	3.0	3.4
**D240**	2.6	2.0	2.0	1.8	1.9	1.9	2.1	2.4	2.2	1.8	2.0	2.4	2.3
**D245**	3.0	2.9	2.9	3.1	3.0	2.9	2.8	2.9	2.8	2.8	2.9	2.8	2.8
**D265**	4.0	4.1	4.1	4.1	4.1	4.1	4.1	4.1	4.0	4.0	4.1	4.0	4.1
**D284**	4.0	3.7	3.0	2.9	2.9	2.8	2.9	3.1	3.1	3.0	3.0	2.8	2.7
**D289**	3.9	3.9	3.9	3.9	3.9	3.9	3.9	3.9	3.9	3.9	3.7	3.8	3.9
**D298**	4.2	3.7	3.4	3.5	3.6	3.6	3.6	3.6	3.6	3.7	3.7	3.6	3.5
**E2**	4.5	4.7	4.1	4.1	3.9	4.1	3.9	3.7	3.7	4.1	4.1	4.0	4.0
**E4**	4.8	3.8	3.5	3.2	3.2	3.3	3.7	3.6	3.2	3.3	3.8	4.3	4.3
**E6**	3.7	3.8	3.8	3.8	3.8	3.8	3.8	3.8	4.0	4.0	3.9	3.7	3.9
**E8**	3.4	4.3	4.2	4.2	4.1	4.2	4.0	3.9	4.2	4.4	4.2	4.0	4.1
**E26**	4.7	4.6	4.5	4.5	4.5	4.5	4.5	4.6	4.6	4.6	4.6	4.6	4.6
**E62**	4.7	4.6	4.5	4.4	4.4	4.4	4.5	4.5	4.5	4.5	4.5	4.5	4.6
**E75**	3.7	4.6	4.1	3.9	4.0	3.9	4.1	4.2	4.3	4.4	4.3	4.3	4.1
**E76**	4.1	4.1	4.0	4.1	4.3	4.2	4.4	4.4	4.1	3.7	3.5	3.2	3.1
**E97**	5.5	4.5	6.0	6.1	5.7	5.9	5.1	4.8	5.1	5.7	6.2	7.2	7.2
**E101**	6.5	7.5	6.5	6.5	6.8	6.9	7.6	7.6	7.3	6.7	6.6	5.9	5.9
**E115**	3.4	4.1	4.0	4.1	4.3	4.4	4.6	4.8	4.7	4.9	4.1	4.2	4.0
**E129**	4.6	3.8	4.0	4.0	4.1	4.1	4.1	3.9	3.7	3.8	3.9	3.5	3.6
**E130**	4.1	4.5	4.3	4.2	4.3	4.3	4.4	4.4	4.4	4.3	4.3	4.4	4.2
**E132**	3.0	3.1	3.2	3.2	3.2	3.1	3.1	3.2	3.4	3.2	3.3	3.3	3.3
**E136**	4.7	4.7	4.6	4.6	4.6	4.6	4.4	4.5	4.4	4.4	4.4	4.5	4.6
**E147**	4.2	4.2	4.3	4.3	4.3	4.3	4.3	4.4	4.3	4.3	4.3	4.3	4.1
**E159**	4.8	4.9	4.9	4.9	4.9	4.9	4.9	4.9	4.9	4.9	4.9	4.8	4.8
**E161**	3.8	4.0	3.9	3.9	3.9	3.9	4.0	4.0	3.8	4.0	4.0	4.0	4.0
**E167**	3.6	3.8	3.7	3.7	3.7	3.6	3.6	3.6	3.6	3.8	4.0	4.4	4.5

**E170**	3.3	3.7	3.6	3.7	3.7	3.7	3.8	3.8	3.7	3.7	3.4	3.3	3.4
**E186**	4.6	4.6	4.4	4.4	4.4	4.4	4.4	4.2	4.2	4.3	4.3	4.4	4.5
**E200**	4.4	4.6	4.4	4.3	4.2	4.3	4.3	4.4	4.4	4.5	4.2	4.1	4.0
**E207**	3.1	4.0	3.6	3.5	3.6	3.5	3.4	3.7	3.6	3.5	3.7	3.9	4.1
**E252**	4.0	3.7	3.7	3.7	3.7	3.6	3.9	4.1	4.0	3.9	4.0	4.0	3.9
**E276**	3.6	3.5	3.6	3.6	3.6	3.7	3.7	3.7	3.7	3.6	3.7	3.8	3.9
**E293**	4.6	4.6	4.6	4.6	4.5	4.5	4.6	4.5	4.5	4.5	4.4	4.4	4.5
**E297**	2.8	2.4	2.7	2.8	3.0	2.9	2.7	2.6	2.6	2.6	2.6	3.2	2.8
**H25**	6.3	6.6	6.5	6.4	6.4	6.4	6.4	6.5	6.5	6.5	6.6	6.5	6.6
**H54**	6.0	6.0	6.0	6.0	6.0	6.0	6.0	5.9	6.0	6.0	6.0	6.0	6.0
**H60**	6.7	6.5	6.6	6.5	6.5	6.5	6.4	6.4	6.4	6.5	6.5	6.6	6.5
**H94**	4.7	4.8	4.8	4.8	4.8	4.8	4.7	4.6	4.7	4.9	4.7	4.6	4.8
**H173**	0.5	1.5	1.0	0.9	0.9	0.9	1.2	1.3	1.2	0.8	1.0	1.2	1.3
**H175**	3.0	3.7	3.4	3.4	3.4	3.5	3.6	3.8	3.7	3.4	3.6	3.9	3.9
**H208**	6.1	5.9	5.9	5.9	5.9	5.9	5.9	6.0	6.0	6.0	6.0	6.0	5.9
**H214**	3.5	3.4	3.3	3.3	3.3	3.3	3.3	3.3	3.4	3.5	3.5	3.4	3.4
**H296**	6.4	6.5	6.6	6.6	6.6	6.6	6.6	6.6	6.5	6.7	6.7	6.6	6.6
**C32**	9.0	10.7	10.2	10.1	9.9	9.9	9.7	9.9	9.9	9.8	10.2	10.5	10.4
**C92**	11.5	11.1	11.3	11.4	11.3	11.3	11.1	11.1	11.3	11.4	11.3	11.4	11.4
**C121**	12.7	11.8	12.1	12.2	12.3	12.3	12.2	12.0	12.1	12.2	12.1	12.2	12.2
**C215**	9.2	8.5	8.7	8.8	8.7	8.7	8.6	8.6	8.5	8.3	8.4	8.3	8.3
**C226**	13.8	13.5	13.4	13.5	13.5	13.5	13.6	13.6	13.6	13.5	13.5	13.6	13.5
**C231**	12.7	12.7	12.6	12.6	12.6	12.6	12.6	12.6	12.7	12.7	12.6	12.7	12.7
**Y20**	10.8	10.5	10.7	10.7	10.6	10.6	10.6	10.6	10.6	10.5	10.6	10.6	10.7
**Y46**	10.9	10.3	10.8	10.9	10.9	10.9	10.6	10.3	10.2	10.1	10.2	10.2	10.1
**Y66**	12.8	12.7	12.7	12.8	12.7	12.8	12.8	12.8	12.7	12.7	12.7	12.6	12.6
**Y81**	15.7	15.7	15.8	15.7	15.7	15.7	15.8	15.9	15.7	15.4	15.6	15.7	15.7
**Y124**	15.4	15.2	15.3	15.3	15.3	15.3	15.3	15.2	15.3	15.3	15.3	15.3	15.4
**Y152**	12.2	11.4	11.5	11.2	11.4	11.2	11.5	11.7	11.8	11.7	11.7	12.1	12.9
**Y153**	14.0	13.8	13.9	13.8	13.8	13.9	14.0	14.1	14.0	13.9	13.7	13.3	13.1
**Y176**	14.2	12.5	13.1	13.1	13.4	13.3	13.6	13.7	13.8	13.6	13.0	13.1	13.0
**Y271**	14.5	14.4	14.5	14.4	14.4	14.5	14.5	14.6	14.5	14.7	14.6	14.6	14.6
**K5**	11.2	11.1	11.1	11.2	11.2	11.0	11.1	11.5	11.6	11.1	11.1	11.0	10.9
**K12**	11.0	10.7	10.8	10.9	11.0	10.9	11.1	11.2	10.9	10.7	10.9	11.1	11.1
**K36**	11.0	10.6	10.6	10.6	10.7	10.7	10.8	10.6	10.6	10.7	10.7	10.6	10.6
**K39**	10.4	10.3	10.3	10.4	10.4	10.4	10.4	10.4	10.4	10.4	10.4	10.4	10.4
**K41**	10.2	10.3	10.3	10.3	10.3	10.3	10.3	10.3	10.4	10.3	10.3	10.2	10.3
**K58**	10.9	10.3	10.4	10.3	10.4	10.3	10.4	10.4	10.4	10.4	10.4	10.4	10.4
**K73**	11.3	10.4	10.8	10.8	10.7	10.8	10.6	10.6	10.6	10.6	10.6	10.7	10.9
**K103**	10.3	10.3	10.3	10.3	10.3	10.3	10.2	10.3	10.3	10.3	10.2	10.2	10.2
**K116**	10.5	10.6	10.6	10.6	10.6	10.6	10.7	10.7	10.6	10.6	10.7	10.5	10.6
**K120**	10.2	10.3	10.5	10.4	10.4	10.4	10.5	10.5	10.5	10.5	10.4	10.3	10.4
**K128**	10.5	10.9	11.0	11.1	11.0	10.9	10.8	11.1	11.2	11.3	11.2	11.4	11.4

**K131**	11.3	11.2	11.2	11.1	11.2	11.2	11.2	11.1	10.9	11.0	11.0	11.1	11.0
**K141**	11.4	11.2	11.3	11.3	11.3	11.3	11.2	11.2	11.3	11.2	11.2	11.2	11.2
**K150**	11.3	11.7	11.4	11.4	11.3	11.5	11.7	11.6	11.5	11.3	11.5	11.4	11.5
**K197**	9.9	10.4	10.3	10.5	10.5	10.4	10.4	10.1	10.2	10.0	10.5	10.8	11.0
**K237**	10.4	10.0	10.4	10.3	10.3	10.2	10.2	10.1	10.3	10.2	10.3	10.4	10.4
**K239**	11.4	11.4	11.3	11.3	11.3	11.2	11.2	11.3	11.2	11.3	11.3	11.3	11.0
**K247**	12.0	12.0	12.0	12.0	12.0	12.0	12.0	11.9	12.0	12.1	12.0	11.9	11.9
**K248**	10.6	10.5	10.7	10.7	10.8	10.8	10.7	10.7	10.7	10.7	10.6	10.7	10.6
**K255**	11.2	11.4	11.3	11.3	11.3	11.3	11.2	11.1	11.3	11.4	11.3	11.3	11.3
**K279**	11.4	11.3	11.3	11.3	11.3	11.3	11.3	11.3	11.3	11.3	11.3	11.3	11.3
**K292**	11.0	12.0	11.1	10.9	10.7	10.8	10.7	10.7	10.8	10.9	11.1	11.4	11.1
**R24**	12.7	12.9	12.6	12.5	12.6	12.5	12.5	12.6	12.6	12.6	12.7	12.7	12.5
**R33**	12.6	12.8	12.7	12.8	12.8	12.7	12.6	12.6	12.6	12.6	12.5	12.7	12.7
**R43**	11.6	11.6	11.6	11.7	11.6	11.6	11.5	11.4	11.5	11.7	11.6	11.4	11.5
**R45**	11.6	11.9	11.6	11.6	11.6	11.6	11.7	11.8	11.8	11.7	11.8	11.6	11.7
**R47**	12.5	13.8	13.7	13.8	13.6	13.5	13.2	13.0	13.0	13.3	13.9	13.9	13.7
**R56**	13.3	13.0	13.0	13.1	13.0	13.0	13.0	13.0	13.0	12.9	13.0	13.1	13.1
**R79**	14.1	13.9	13.7	13.8	13.9	13.8	13.8	13.8	14.0	14.4	14.1	14.0	14.1
**R105**	12.8	12.9	12.8	12.8	12.8	12.8	12.7	12.8	12.7	12.7	12.8	12.9	12.7
**R112**	12.3	12.5	12.4	12.4	12.4	12.4	12.5	12.7	12.7	12.7	12.7	12.5	12.7
**R156**	11.4	11.8	11.6	11.6	11.5	11.5	11.6	11.5	11.5	11.5	11.4	11.6	11.8
**R169**	12.5	12.3	12.3	12.3	12.3	12.4	12.5	12.5	12.4	12.3	12.1	11.8	11.7
**R199**	12.7	12.7	12.6	12.6	12.6	12.6	12.6	12.6	12.6	12.7	12.7	12.7	12.7
**R221**	12.8	12.4	12.5	12.6	12.6	12.6	12.5	12.3	12.2	12.0	12.1	12.5	12.4
**R238**	12.7	12.8	12.7	12.7	12.6	12.6	12.4	12.4	12.8	13.2	13.2	13.5	13.5
**R254**	11.0	11.0	11.3	11.4	11.4	11.4	11.3	11.3	11.1	11.1	11.1	11.2	11.2
**R257**	10.1	10.2	10.2	10.2	10.2	10.2	10.2	10.2	10.2	10.2	10.2	10.2	10.2
**R268**	13.7	13.6	13.7	13.7	13.8	13.7	13.8	13.8	13.8	13.8	13.9	13.8	13.8

[a] pK_a calculations were performed on our PT-MetaD-WTE simulations of PTP1B in complex with *p*NPP, and estimated using PROPKA v3.1.[10] For each *Q*-value, 100 frames were used to calculate the average value. The native contact value (*Q*) is used to define how similar the WPD-loop is to the closed state crystal structure reference, with values closer to 1 meaning the WPD-loop closed state (see the **Supplementary Methodology** for further details about how the *Q*-value is defined). The general acid, D181, is highlighted in bold red.

Table S15. The impact of the WPD-loop conformation on the predicted pK_a values of all ionizable residues in YopH.[a]

Residue Number	Native Contact (Q) Value												
	**1**	**0.99**	**0.98**	**0.97**	**0.96**	**0.95**	**0.9**	**0.85**	**0.8**	**0.7**	**0.6**	**0.5**	**0.4**
**D214**	1.4	1.4	1.5	1.4	1.4	1.5	1.5	1.6	1.5	1.5	1.5	1.5	1.5
**D231**	3.4	3.7	3.6	3.6	3.5	3.5	3.2	3.3	3.4	3.3	3.0	3.0	3.0
**D243**	2.2	2.4	2.6	2.6	2.7	2.6	2.3	2.3	2.6	2.4	2.1	2.3	2.2
**D300**	3.4	3.4	3.1	3.0	3.0	3.0	3.0	3.1	3.1	3.0	3.0	3.1	3.0
**D325**	3.5	3.6	3.7	3.8	3.8	3.8	3.7	3.7	3.7	3.6	3.8	3.8	3.6
**D330**	3.8	3.6	3.6	3.5	3.5	3.6	3.7	3.6	3.6	3.5	3.7	3.9	3.8
**D356**	4.7	4.4	4.5	4.3	4.4	4.3	4.2	4.2	4.0	4.2	4.1	4.2	3.9
**D373**	4.5	4.7	4.7	4.7	4.7	4.7	4.7	4.6	4.7	4.7	4.6	4.9	4.8
**D393**	2.1	2.1	1.9	1.9	1.9	1.8	1.8	2.0	2.0	1.7	2.0	1.9	2.2
**D394**	2.6	2.7	2.9	3.0	3.0	3.0	2.9	3.1	3.2	3.0	3.1	3.2	3.0
**D421**	1.8	2.0	2.0	2.0	2.0	2.0	2.1	2.2	2.0	2.0	2.1	2.0	2.1
**D431**	3.6	3.8	3.6	3.6	3.6	3.6	3.6	3.5	3.6	3.3	3.2	3.6	3.5
**D448**	4.0	3.9	4.0	4.0	4.0	4.0	4.0	4.0	4.0	3.9	3.8	4.0	4.0
**D452**	3.3	3.3	3.3	3.2	3.3	3.2	3.0	3.1	3.2	3.2	3.2	3.5	3.4
**E192**	4.7	4.6	4.5	4.5	4.5	4.5	4.5	4.5	4.3	4.4	4.2	4.0	4.1
**E196**	3.9	3.9	3.9	3.8	3.8	3.9	3.9	3.9	3.9	3.9	3.8	3.9	3.9
**E224**	4.6	4.4	4.4	4.4	4.4	4.5	4.1	4.1	4.3	4.5	4.5	4.2	4.2
**E268**	3.0	3.1	3.1	3.1	3.1	3.2	3.2	3.4	3.4	3.1	3.3	3.4	3.4
**E276**	3.8	3.8	3.9	4.0	4.0	4.1	4.0	4.0	3.9	3.9	4.0	3.9	3.9
**E290**	2.1	2.3	2.2	2.2	2.2	2.2	2.3	2.2	2.3	2.1	2.4	2.5	2.5
**E314**	3.6	3.7	3.8	3.8	3.8	3.8	3.8	3.9	3.9	3.8	3.6	3.7	3.7
**E338**	3.1	3.5	3.7	3.8	3.7	3.7	3.4	3.5	3.2	3.4	2.7	3.2	3.1
**E363**	4.1	3.8	3.8	3.8	3.8	3.9	3.8	3.8	4.0	4.0	4.2	3.8	3.9
**E377**	3.8	3.9	4.0	4.1	4.1	4.0	3.6	3.6	3.6	3.7	3.3	3.2	3.1
**E384**	4.7	4.7	4.3	4.3	4.3	4.3	4.2	4.3	4.1	4.2	4.0	3.9	3.8
**E430**	3.2	3.5	3.7	3.7	3.6	3.6	3.6	3.7	3.7	3.9	4.0	4.0	3.9
**E449**	4.2	4.0	4.1	4.0	4.0	3.9	3.8	3.9	4.0	4.3	4.2	4.1	4.2
**E459**	3.9	4.0	4.0	4.1	4.2	4.2	4.3	4.3	4.3	4.3	4.3	4.0	4.1
**H270**	2.7	2.7	2.8	2.8	2.8	2.8	2.7	2.7	2.7	2.7	2.7	2.8	2.8
**H350**	4.7	4.7	4.7	4.7	4.7	4.7	4.8	4.7	4.7	4.7	4.5	4.4	4.4
**H402**	3.3	3.3	3.1	3.2	3.2	3.2	3.4	3.2	3.3	3.3	3.4	3.2	3.3
**C221**	9.1	9.1	9.1	9.1	9.1	9.1	9.2	9.4	9.7	9.6	9.4	9.4	9.4
**C234**	11.2	11.1	11.1	11.1	11.1	11.1	10.8	10.8	10.7	10.7	11.0	10.9	11.0
**C259**	12.1	12.1	12.1	12.0	12.1	12.0	12.0	12.1	12.0	12.1	12.1	12.0	11.9
**C403**	9.0	9.2	9.1	9.1	9.1	9.1	8.7	8.9	9.0	8.7	8.7	8.7	8.6
**C418**	11.5	11.5	11.5	11.5	11.5	11.4	11.4	11.3	11.5	11.6	11.4	11.3	11.3

**Y189**	10.7	10.8	10.7	10.8	10.7	10.8	10.7	10.7	10.7	10.7	10.9	10.5	10.5
**Y217**	11.3	11.4	11.4	11.5	11.4	11.5	11.4	11.4	11.3	11.3	11.5	11.5	11.4
**Y248**	10.7	10.7	10.8	10.9	10.8	10.8	10.8	10.8	10.7	10.7	10.8	10.8	10.7
**Y261**	13.5	13.5	13.5	13.4	13.4	13.4	13.5	13.5	13.5	13.5	13.6	13.5	13.4
**Y301**	15.6	15.5	15.6	15.6	15.6	15.5	15.6	15.6	15.6	15.5	15.6	15.5	15.5
**Y308**	10.9	11.0	10.9	11.0	11.0	11.0	10.9	10.9	11.0	11.0	11.0	11.0	11.0
**Y332**	11.0	10.9	10.9	10.9	10.9	10.9	10.9	10.9	10.9	10.8	10.8	10.9	11.1
**Y383**	11.3	11.3	11.3	11.3	11.3	11.3	11.3	11.2	11.2	11.3	11.3	11.3	11.4
**K225**	10.3	10.3	10.3	10.3	10.3	10.3	10.3	10.3	10.3	10.4	10.3	10.3	10.4
**K316**	10.5	10.4	10.4	10.4	10.4	10.4	10.5	10.5	10.5	10.6	10.8	10.6	10.5
**K342**	10.1	10.2	10.3	10.3	10.3	10.3	10.3	10.3	10.4	10.5	10.6	10.5	10.6
**K366**	10.9	10.9	10.9	11.0	10.9	10.9	10.8	10.8	10.5	10.8	10.7	10.9	11.0
**K379**	9.4	9.5	9.5	9.6	9.5	9.6	9.5	9.5	9.5	9.6	9.6	9.4	9.5
**K386**	10.4	10.4	10.4	10.4	10.4	10.4	10.4	10.4	10.4	10.4	10.4	10.4	10.4
**K396**	11.4	11.3	11.3	11.3	11.3	11.3	11.4	11.3	11.3	11.6	11.3	11.4	11.4
**K447**	10.5	10.8	10.6	10.7	10.6	10.7	10.6	10.7	10.7	10.4	10.4	10.6	10.6
**K456**	11.2	11.1	11.1	11.1	11.0	11.0	11.1	11.0	11.0	11.1	10.8	11.1	11.1
**R194**	13.2	13.1	13.1	13.1	13.1	13.0	13.1	13.1	13.1	13.1	13.2	12.8	12.9
**R200**	14.3	14.4	14.3	14.3	14.3	14.2	14.3	14.3	14.3	14.3	14.3	14.4	14.6
**R205**	11.9	12.0	12.0	12.1	12.1	12.1	12.2	12.2	12.2	12.1	12.1	11.8	11.9
**R216**	14.0	14.0	13.9	14.0	14.0	13.9	13.9	13.9	14.0	13.9	13.9	13.9	14.0
**R228**	10.8	10.8	10.8	10.8	10.8	10.8	11.2	11.0	11.0	11.1	11.1	11.0	10.9
**R230**	13.0	12.8	12.8	12.8	12.9	12.9	13.2	13.1	13.0	13.1	13.3	13.4	13.4
**R235**	12.0	11.9	12.0	11.9	12.0	11.9	11.9	11.9	11.9	11.9	11.9	11.8	11.9
**R236**	12.3	12.2	12.2	12.2	12.2	12.2	12.2	12.2	12.2	12.2	12.2	12.2	12.2
**R241**	13.4	13.2	13.1	13.0	13.0	13.1	13.2	13.3	13.0	13.2	13.5	13.5	13.5
**R255**	13.3	13.1	12.9	12.7	12.7	12.7	12.7	12.6	12.6	12.6	12.6	12.5	12.7
**R272**	14.1	13.9	13.7	13.7	13.8	13.7	14.1	13.9	14.0	13.9	14.0	14.3	14.3
**R278**	12.2	12.4	12.4	12.4	12.4	12.4	12.3	12.3	12.4	12.5	12.4	12.4	12.4
**R295**	12.4	12.4	12.4	12.4	12.4	12.4	12.4	12.4	12.4	12.4	12.4	12.4	12.4
**R303**	13.3	13.2	13.2	13.2	13.2	13.3	13.2	13.4	13.5	13.4	13.4	13.4	13.3
**R337**	13.4	13.2	13.2	13.1	13.1	13.0	12.8	12.7	12.8	12.8	12.5	12.6	12.7
**R380**	12.7	12.5	12.9	12.9	12.9	13.1	13.7	13.5	13.7	13.8	14.4	14.8	14.9
**R398**	11.1	11.1	11.0	10.9	11.0	11.0	11.1	11.0	11.0	10.9	11.0	11.0	11.2
**R404**	12.4	12.2	12.2	12.2	12.3	12.3	12.1	12.2	12.3	12.2	11.9	11.9	11.8
**R409**	13.9	13.7	13.7	13.7	13.8	13.8	13.8	13.8	13.8	13.8	13.9	14.0	14.1
**R423**	13.8	13.8	13.8	13.8	13.8	13.8	13.8	13.8	13.8	13.7	13.9	13.9	13.8
**R437**	10.0	9.9	10.0	10.0	10.0	10.0	10.1	10.1	10.1	10.1	10.1	10.0	10.0
**R440**	11.0	11.0	11.0	11.0	11.0	11.0	11.2	11.3	11.4	11.2	11.0	11.1	11.1
**R463**	11.5	11.5	11.5	11.5	11.5	11.5	11.5	11.5	11.5	11.5	11.7	11.7	11.7

[a] pK_a calculations were performed on our PT-MetaD-WTE simulations of YopH in complex with *p*NPP, and estimated using PROPKA v3.1.[10] For each *Q*-value, 100 frames were used to calculate the average value. The native contact value (*Q*) is used to define how similar the WPD-loop is to the closed state crystal structure reference, with values closer to 1 meaning the WPD-loop closed state (see the **Supplementary Methodology** for further details on how the *Q*-value is defined). The general acid, D356, is highlighted in bold red.

S5. Empirical Valence Bond Parameters

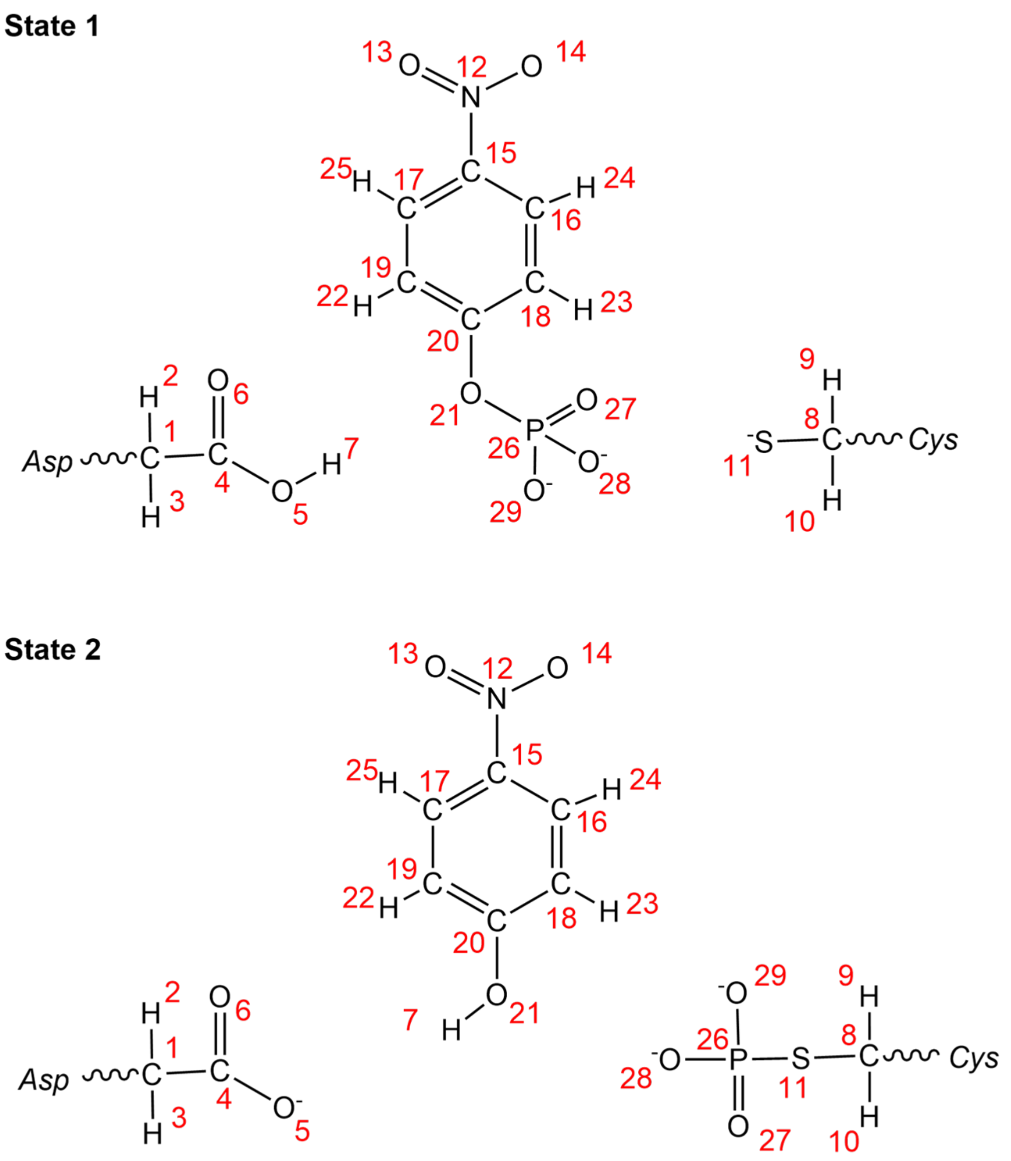

Figure S23. Atom numbering of the valence bond states used to describe the cleavage (*i.e.*, first chemical step) reaction catalyzed by PTP1B and YopH (see **Figure 1** in the main text for the full reaction mechanism).

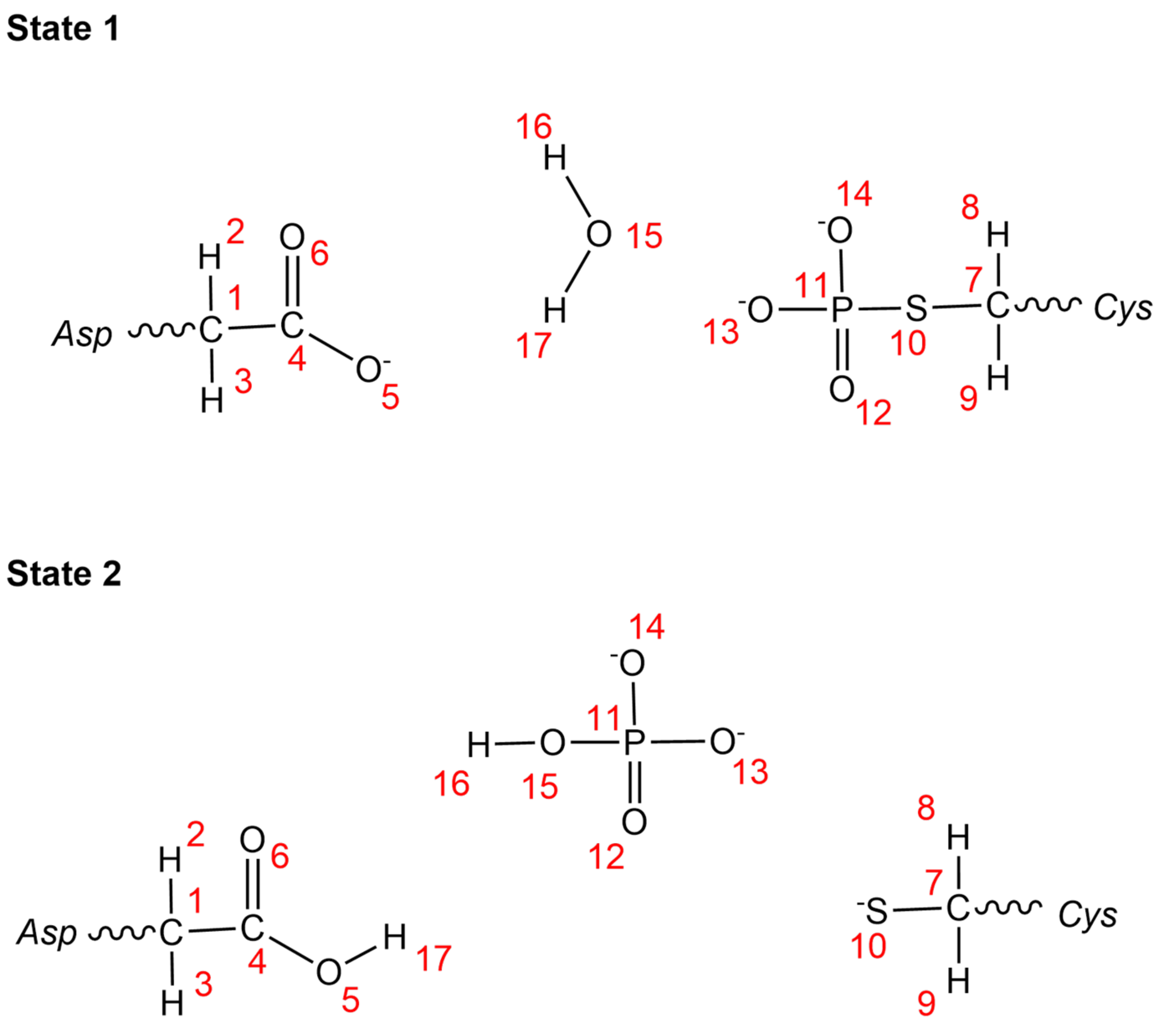

Figure S24. Atom numbering of the valence bond states used to describe the hydrolysis reaction (*i.e.*, second chemical step) catalyzed by PTP1B and YopH (see **Figure 1** in the main text for the full reaction mechanism).

Table S16. Empirical valence bond parameters used to describe the PTP-catalyzed reactions.[a]

	$\Delta G^{\ddagger}$	ΔG_0	H_{ij}	α
**Cleavage**	29.6 ± 0.1	-1.4 ± 0.2	162.5	54.5
**Hydrolysis**	35.2 ± 0.2	-10.1 ± 0.2	133.5	47.8

[a] Shown here are the are averages and standard error of the mean of calculated activation ($\Delta G^{\ddagger}$) and reaction (ΔG_0) free energies for the non-enzymatic model systems used to describe the cleavage and hydrolysis steps (**Figure 1**), respectively, and the values of H_{ij} and α calibrated to reproduce these energies. All energies are shown in kcal mol^{-1}. For details of the calibration, see the **Methodology** section, and for a detailed description of the physical meaning of these parameters, see *e.g.*, refs. 51,136.

Table S17. List of the atom types and van der Waals parameters in our EVB simulations to describe the atoms constituting the reacting parts of the system.[a]

Atom Type	A_i (kcal$^{1/2}$ mol$^{1/2}$ Å^6)	B_i (kcal$^{1/2}$ mol$^{1/2}$ Å^3)	C_i (kcal mol^{-1})	α_i (Å^2)	$A_{1\text{-}4}$ (kcal$^{1/2}$ mol$^{1/2}$ Å^3)	$B_{1\text{-}4}$ (kcal$^{1/2}$ mol$^{1/2}$ Å^3)	Mass (a.u.)
CA	1059.1297	23.6736			748.9178	16.7398	12.011
CT	944.518	22.0296			667.8751	15.5773	12.011
C2	1802.2385	34.1758			1274.3751	24.1659	12.011
HA	69.5797	4.9095			49.2003	3.4715	1.0079
HC	84.5728	5.4127			59.802	3.8274	1.0079
H	0.0054	0.0433			0.0038	0.0306	1.0079
HO	0.0054	0.0433	92	1.8	0.0038	0.0306	1.0079
N	971.7502	28.3077			687.1312	20.0166	14.007
O1	616.4387	23.7692			435.888	16.8074	15.999
O2	616.4387	23.7692	90	2.5	435.888	16.8074	15.999
ODE	601.1488	22.2648	90	1.5	425.076	15.7436	15.999
O3	401.0174	17.3232	90	2.5	283.5621	12.2494	15.999
O4	690.3744	23.86	90	2.5	488.1684	16.8716	15.999
ON	554.6317	21.386			392.1838	15.1222	15.999
OP	626.3868	23.6698			442.9224	16.7371	15.999
OW	793.3991	25.0106	90	2.5	561.0179	17.6852	15.999
SG	2593.9355	55.597	91	2.5	1834.1894	39.313	32.065
P	2447.7925	46.7907	110	1.7	1730.8507	33.086	30.974

[a] A standard 6-12 Lennard-Jones potential was used for all other atoms except the reacting atoms. In the case of the reacting atoms, which change bonding patterns between atoms i and j, an alternate function of the form $V_{react} = C_i C_j \exp(-\alpha_i \alpha_j r_{ij})$ was used to prevent artificial repulsion between these atoms as bonding patterns change. r_{ij} denotes the distance (Å) between atoms i and j. Atom type assignments to the atoms in our EVB simulations are provided in **Table S22**.

Table S18. Bond types and corresponding parameters used to describe the covalent bonds constituting the reacting part of the system. [a]

Bond Type	K_b (kcal mol^{-1} Å^{-2})	r_0 (Å)	D (kcal mol^{-1})	α (Å^{-2})	r_0 (Å)
1	900	1.364			
2	1312	1.25			
3	1140	1.229			
4			200	1.25	0.945
5			245.8	1.5	0.945
6			30	1	2.163
7			95	1.75	1.61
8	1106	0.945			
9	2000	1.0			
10	938	1.4			
11	800	1.46			
12	734	1.08			
13	1100	1.225			
14	1050	1.516			
15	680	1.09			
16	444	1.81			
17	634	1.522			

[a] Bonds between non-reacting atoms are described using harmonic potentials, $V_{harmonic} = 0.5K_b\left(r_{ij} - r_0\right)^2$,while bonds between reacting atoms are described using Morse potentials, $V_{morse} = D\left(1 - exp\left(-\alpha\left(r_{ij} - r_0\right)\right)\right)^2$. Note that a bond type assignment of '0' indicates that a bond has been broken in that VB state. Bond type assignments to the atoms in our EVB simulations are provided in **Table S17**.

Table S19. Angle types and their corresponding parameters used to describe the bending of adjacent bonds in the reacting part of the system.[a]

Angle Type	K_a (kcal mol^{-1} rad^{-2})	Θ (°)
1	160.0	120.4
2	140.0	117.0
3	70.0	113.0
4	150.0	100.0
5	160.0	114.0
6	150.0	115.923
7	200.0	107.0
8	400.0	109.47
9	130.0	92.754
10	126.0	120.0
11	70.0	120.0
12	160.0	117.5
13	170.0	120.0
14	140.0	120.0
15	160.0	125.3
16	66.0	107.8
17	70.0	109.5
18	280.0	114.0

[a] The angle potential is described using the potential $V_{angle} = 0.5 \sum k_a(\Theta - \Theta_0)^2$. Note that an angle type assignment of '0' indicates that an angle has been broken in that VB state. Angle type assignments to the atoms in our EVB simulations are provided in **Table S25**.

Table S20. Torsion angle types and the corresponding parameters used to describe the rotation of dihedral angles in the reacting part of the system.[a]

Torsion Angle Type	K_φ (kcal mol^{-1} rad^{-2})	n	φ_0 (°)
1	0.583	2	180
2	0.273	2	180
3	1.5	-1	0
4	2.45	2	180
5	0	2	180
6	0	1	0
7	0.841	2	180
8	-2.6555	-1	0
9	-0.2235	-2	180
10	0.2415	3	0
11	0.15	3	0
12	0.24	3	0
13	0.547	2	180
14	0	1	0
15	2.45	2	180
16	3.625	2	180
17	0.6995	2	180

[a] The torsion angle potential is described using the potential $V_{torsion} = K_\varphi(1 + \cos(n\varphi - \varphi_0))$. Note that a torsion angle type assignment of '0' indicates that a torsion angle has been broken in that VB state. Torsion angle type assignments to the atoms in our EVB simulations are provided in **Table S26**.

Table S21. Improper torsion types and the corresponding parameters in the reacting part of the system.[a]

Improper Torsion Angle Type	k_i (kcal mol^{-1} rad^{-2})	τ_0 (°)
1	1.1	180
2	10.5	180
3	4.0	180

[a] The improper torsion potential is described using the potential $V_{improper} = k_i(\tau - \tau_0)^2$, where k_i is the force constant and τ is the equilibrium angle (in degrees). Note that an improper torsion angle type assignment of '0' indicates that a bond has been broken in that VB state.

Table S22. Atom types in the different valence bond states used to describe the PTP-catalyzed reaction.[a]

Cleavage				Hydrolysis		
**Atom Number**	**Atom Type**			**Atom Number**	**Atom Type**	
	**State 1**	**State 2**			**State 1**	**State 2**
1	CT	CT		1	CT	CT
2	HC	HC		2	HC	HC
3	HC	HC		3	HC	HC
4	C2	C2		4	C2	C2
5	ODE	O2		5	O2	ODE
6	O1	O2		6	O2	O1
7	HO	HO		7	CT	CT
8	CT	CT		8	HC	HC
9	HC	HC		9	HC	HC
10	HC	HC		10	SG	SG
11	SG	SG		11	P	P
12	N	N		12	OP	OP
13	ON	ON		13	OP	OP
14	ON	ON		14	OP	OP
15	CA	CA		15	OW	O3
16	CA	CA		16	H	HO
17	CA	CA		17	HO	HO
18	CA	CA				
19	CA	CA				
20	CA	CA				
21	O3	O4				
22	HA	HA				
23	HA	HA				
24	HA	HA				
25	HA	HA				
26	P	P				
27	OP	OP				
28	OP	OP				
29	OP	OP				

[a] Corresponding parameters for each atom type can be found in **Table S17**.

Table S23. Partial charges for the different valence bond states used to describe the PTP-catalyzed reaction.

Cleavage				Hydrolysis		
Atom Number	Atom Type			Atom Number	Atom Type	
	State 1	State 2			State 1	State 2
1	-0.1200	-0.2200		1	-0.2200	-0.1200
2	0.0600	0.0600		2	0.0600	0.0600
3	0.0600	0.0600		3	0.0600	0.0600
4	0.5200	0.7000		4	0.7000	0.5200
5	-0.5300	-0.8000		5	-0.8000	-0.5300
6	-0.4400	-0.8000		6	-0.8000	-0.4400
7	0.4500	0.4299		7	0.0454	-0.2545
8	-0.2545	0.0454		8	0.0380	0.0992
9	0.0992	0.0380		9	0.0380	0.0992
10	0.0992	0.0380		10	-0.6622	-0.9440
11	-0.9440	-0.6622		11	1.2423	1.3832
12	0.7963	0.3191		12	-0.9003	-0.9678
13	-0.5529	-0.2121		13	-0.9003	-0.9678
14	-0.5529	-0.2121		14	-0.9003	-0.9678
15	-0.1338	-0.2193		15	-0.8340	-0.7953
16	-0.1244	-0.0281		16	0.4170	0.3154
17	-0.1244	-0.0281		17	0.4170	0.4500
18	-0.4416	-0.1996				
19	-0.4416	-0.1996				
20	0.9050	0.1700				
21	-0.7123	-0.4833				
22	0.1863	0.1580				
23	0.1863	0.1580				
24	0.1694	0.1734				
25	0.1694	0.1734				
26	1.3862	1.2423				
27	-0.9050	-0.9003				
28	-0.9050	-0.9003				
29	-0.9050	-0.9003				

Table S24. Bond types used to describe the covalent bonds in the reacting part of the system.[a]

Cleavage					Hydrolysis			
Atom Number		Bond Type			Atom Number		Bond Type	
#1	#2	State 1	State 2		#1	#2	State 1	State 2
4	5	1	2		4	5	2	1
4	6	3	2		4	6	2	3
5	7	4	0		5	17	0	4
7	21	0	5		10	11	6	0
11	26	0	6		11	15	0	7
21	26	7	0		15	16	9	8
15	16	10	10		15	17	5	0
15	17	10	10		7	8	15	15
15	12	11	11		7	9	15	15
16	18	10	10		7	10	16	16
16	24	12	12		1	4	17	17
17	19	10	10		1	2	15	15
17	25	12	12		1	3	15	15
18	20	10	10		12	11	14	14
18	23	12	12		13	11	14	14
19	20	10	10		14	11	14	14
19	22	12	12					
20	21	1	1					
12	13	13	13					
12	14	13	13					
27	26	14	14					
28	26	14	14					
29	26	14	14					
8	9	15	15					
8	10	15	15					
8	11	16	16					
1	4	17	17					
1	2	15	15					
1	3	15	15					

[a] Corresponding parameters for each bond can be found in **Table S18**.

Table S25. Angle types used to describe the angles in the reacting part of the system.[a]

Cleavage						Hydrolysis				
Atom Number			Angle Type			Atom Number			Angle Type	
#1	#2	#3	State 1	State 2		#1	#2	#3	State 1	State 2
1	4	5	1	2		1	4	5	2	1
4	5	7	3	0		4	5	17	0	3
7	21	20	0	3		17	10	11	4	0
8	11	26	0	4		10	11	12	5	0
11	26	27	0	5		10	11	13	5	0
11	26	28	0	5		10	11	14	5	0
11	26	29	0	5		11	15	16	0	9
20	21	26	6	0		12	11	15	0	7
21	26	27	7	0		13	11	15	0	7
21	26	28	7	0		14	11	15	0	7
21	26	29	7	0		16	15	17	8	0
15	16	18	10	10		9	7	8	16	16
15	16	24	11	11		9	7	10	17	17
15	17	19	10	10		8	7	10	17	17
15	17	25	11	11		12	11	13	18	18
15	12	13	12	12		12	11	14	18	18
15	12	14	12	12		13	11	14	18	18
16	15	17	10	10		4	1	2	17	17
16	15	12	13	13		4	1	3	17	17
16	18	20	10	10		2	1	3	16	16
16	18	23	11	11						
17	15	12	13	13						
17	19	20	10	10						
17	19	22	11	11						
18	16	24	11	11						
18	20	19	10	10						
18	20	21	14	14						
19	17	25	11	11						
19	20	21	14	14						
20	18	23	11	11						
20	19	22	11	11						
14	12	13	16	16						
10	8	9	16	16						
10	8	11	17	17						
9	8	11	17	17						
27	26	28	18	18						
27	26	29	18	18						
28	26	29	18	18						

4	1	2	17	17						
4	1	3	17	17						
2	1	3	16	16						

[a] Corresponding parameters for each angle can be found in **Table S19**.

Table S26. Torsion angle types used to describe the torsion angles in the reacting part of the system.[a]

Cleavage

Atom Number				Torsion Angle Type	
**#1**	**#2**	**#3**	**#4**	**State 1**	**State 2**
1	4	5	7	3	0
1	4	5	7	4	0
3	1	4	6	0	6
3	1	4	6	5	0
2	1	4	6	0	6
2	1	4	6	5	0
6	4	5	7	4	0
7	21	20	18	0	7
7	21	20	19	0	7
8	11	26	27	0	11
8	11	26	28	0	11
8	11	26	29	0	11
9	8	11	26	0	12
10	8	11	26	0	12
18	20	21	26	13	0
19	20	21	26	13	0
20	21	26	27	14	0
20	21	26	28	14	0
20	21	26	29	14	0
C_{Asp}[b]	1	4	5	1	2
C_{Cys}[c]	8	11	26	0	8
C_{Cys}	8	11	26	0	9
C_{Cys}	8	11	26	0	10
15	16	18	20	16	16
15	16	18	23	16	16
15	17	19	20	16	16
15	17	19	22	16	16
16	15	17	19	16	16
16	15	17	25	16	16
16	15	12	13	17	17
16	15	12	14	17	17
16	18	20	19	16	16
16	18	20	21	16	16
17	15	16	18	16	16
17	15	16	24	16	16
17	15	12	13	17	17
17	15	12	14	17	17

Hydrolysis

Atom Number				Torsion Angle Type	
**#1**	**#2**	**#3**	**#4**	**State 1**	**State 2**
1	4	5	17	0	3
1	4	5	17	0	4
3	1	4	6	6	0
3	1	4	6	0	5
2	1	4	6	6	0
2	1	4	6	0	5
6	4	5	17	0	15
7	10	11	12	11	0
7	10	11	13	11	0
7	10	11	14	11	0
8	7	10	11	12	0
9	7	10	11	12	0
12	11	15	16	0	14
13	11	15	16	0	14
14	11	15	16	0	14
C_{Asp}[b]	1	4	5	2	1
C_{Cys}[c]	7	10	11	8	0
C_{Cys}	7	10	11	9	0
C_{Cys}	7	10	11	10	0

17	19	20	18	16	16							
17	19	20	21	16	16							
18	16	15	12	16	16							
18	20	19	22	16	16							
19	17	15	12	16	16							
19	20	18	23	16	16							
20	18	16	24	16	16							
20	19	17	25	16	16							
24	16	15	12	16	16							
24	16	18	23	16	16							
25	17	15	12	16	16							
25	17	19	22	16	16							
23	18	20	21	16	16							
33	19	20	21	16	16							

[a] Corresponding parameters for the defined torsions can be found in **Table S20**. [b] C_{Asp} denotes the C_α carbon of the catalytic aspartic acid, [c] C_{cys} denotes the C_γ carbon of the catalytic cysteine. These two atoms are not part of the reacting system.

Table S27. Improper torsion angle types used to describe the improper torsion angles in the reacting part of the system.[a]

Atom Number				Improper Torsion Angle Type			
				Cleavage		Hydrolysis	
**#1**	**#2**	**#3**	**#4**	**State 1**	**State 2**	**State 1**	**State 2**
1	4	5	6	2	2	2	2
15	16	18	24	1	1		
15	17	19	25	1	1		
15	12	13	14	2	2		
16	15	17	12	3	3		
16	18	20	23	1	1		
17	19	20	22	1	1		
18	20	19	21	3	3		

[a] Corresponding parameters for each torsion angle can be found in **Table S21**.

S6. Supplementary References

(1) Berman, H. M.; Westbrook, J.; Feng, Z.; Gilliland, G.; Bhat, T. N.; Weissig, H.; Shindyalov, I. N.; Bourne, P. E. The Protein Data Bank. *Nucleic Acids Res.* **2000**, *28*, 235–242.

(2) Roe, D. R.; Cheatham, T. E. PTRAJ and CPPTRAJ: Software for Processing and Analysis of Molecular Dynamics Trajectory Data. *J. Chem. Theory Comput.* **2013**, *9*, 3084–3095.

(3) Case, D.A.; Betz, R.M.; Cerutti, D.S.; Cheatham, III, T.E.; Darden, R.E.; Duke, T.J.; Gohlke, H.; Goetz, A.W.; Homeyer, N.; Izadi, S.; Janowski, P.; Kaus, J.; Kovalenko, A.; Lee, T.S.; LeGrand, S.; Li, P.; Lin, C.; Luchko, T.; Luo, R.; Madej, B.; Mermelstein, D.; Merz, K.M.; Monard, G.; Nguyen, H.; Nguyen, H.T.; Omelyan, I.; Onufriev, A.; Roe, D.R.; Roitberg, A.; Sagui, C.; Simmerling, C. L.; Botello-Smith, W.M.; Swails, J.; Walker, R.C.; Wang, J.; Wolf, R.M.; Wu, X.; Xiao, L.; Kollman, P.A. Amber 2016. University of California, San Francisco: San Francisco 2016.

(4) Moise, G.; Johnson, S. J.; Caradonna, T.; Loria, J. P.; Beaumont, V.; Hengge, A. C.; Morales, Y. A YopH PTP1B Chimera Shows the Importance of the WPD-Loop Sequence to the Activity, Structure, and Dynamics of Protein Tyrosine Phosphatases. *Biochemistry* **2018**, *57*, 5315–5326.

(5) Phan, J.; Lee, K.; Cherry, S.; Tropea, J. E.; Burke, T. R.; Waugh, D. S. High-Resolution Structure of the Yersinia Pestis Protein Tyrosine Phosphatase YopH in Complex with a Phosphotyrosyl Mimetic-Containing Hexapeptide. *Biochemistry* **2003**, *42*, 13113–13121.

(6) Stuckey, J. A.; Schubert, H. L.; Fauman, E. B.; Zhang, Z.-Y.; Dixon, J. E.; Saper, M. A. Crystal Structure of Yersinia Protein Tyrosine Phosphatase at 2.5 Å and the Complex with Tungstate. *Nature* **1994**, *370*, 571–575.

(7) Denu, J. M.; Lohse, D. L.; Vijayalakshmi, J.; Saper, M. A.; Dixon, J. E. Visualization of Intermediate and Transition-State Structures in Protein-Tyrosine Phosphatase Catalysis. *Proc. Natl. Acad. Sci. USA* **1996**, *93*, 2493–2498.

(8) Keedy, D. A.; Hill, Z. B.; Biel, J. T.; Kang, E.; Rettenmaier, T. J.; Brandão-Neto, J.; Pearce, N. M.; von Delft, F.; Wells, J. A.; Fraser, J. S. An Expanded Allosteric Network in PTP1B by Multitemperature Crystallography, Fragment Screening, and Covalent Tethering. *eLife* **2018**, *7*, 1–36.

(9) Brandão, T. A. S.; Hengge, A. C.; Johnson, S. J. Insights into the Reaction of Protein-Tyrosine Phosphatase 1B. *J. Biol. Chem.* **2010**, *285*, 15874–15883.

(10) Søndergaard, C. R.; Olsson, M. H. M.; Rostkowski, M.; Jensen, J. H. Improved Treatment of Ligands and Coupling Effects in Empirical Calculation and Rationalization of pK_a Values. *J. Chem. Theory Comput.* **2011**, *7*, 2284–2295.

(11) Chen, V. B.; Arendall, W. B.; Headd, J. J.; Keedy, D. A.; Immormino, R. M.; Kapral, G. J.; Murray, L. W.; Richardson, J. S.; Richardson, D. C. MolProbity: All-Atom Structure Validation for Macromolecular Crystallography. *Acta Crystallogr. Sect. D Biol. Crystallogr.* **2010**, *66*, 12–21.

(12) Wang, J.; Wang, W.; Kollman, P. A.; Case, D. A. Automatic Atom Type and Bond Type Perception in Molecular Mechanical Calculations. *J. Mol. Graph. Model.* **2006**, *25*, 247–260.

(13) Frisch, M. J.; et al; Trucks, G. W.; Schlegel, H. B.; Scuseria, G. E.; Robb, M. A.; Cheeseman, J. R.; Scalmani, G.; Barone, V.; Petersson, G. A.; Nakatsuji, H.; Li, X.; Fox, D. J.; et al. Gaussian16, Revision A.03. Gaussian Inc.: Wallingford CT 2016.

(14) Wang, J.; Wolf, R. M.; Caldwell, J. W.; Kollman, P. A.; Case, D. A. Development and Testing of a General Amber Force Field. *J. Comput. Chem.* **2004**, *25*, 1157–1174.

(15) Cossi, M.; Rega, N.; Scalmani, G.; Barone, V. Energies, Structures, and Electronic Properties of Molecules in Solution with the C-PCM Solvation Model. *J. Comput. Chem.* **2003**, *24*, 669–681.

(16) Maier, J. A.; Martinez, C.; Kasavajhala, K.; Wickstrom, L.; Hauser, K. E.; Simmerling, C. FF14SB: Improving the Accuracy of Protein Side Chain and Backbone Parameters from FF99SB. *J. Chem. Theory Comput.* **2015**, *11*, 3696–3713.

(17) Kaminski, G. A.; Friesner, R. A.; Tirado-Rives, J.; Jorgensen, W. L. Evaluation and Reparametrization of the OPLS-AA Force Field for Proteins via Comparison with Accurate Quantum Chemical Calculations on Peptides. *J. Phys. Chem. B* **2001**, *105*, 6474–6487.

(18) Jorgensen, W. L.; Maxwell, D. S.; Tirado-Rives, J. Development and Testing of the OPLS All-Atom Force Field on Conformational Energetics and Properties of Organic Liquids. *J. Am. Chem. Soc.* **1996**, *118*, 11225–11236.

(19) Ben-David, M.; Soskine, M.; Dubovetskyi, A.; Cherukuri, K.-P.; Dym, O.; Sussman, J. L.; Liao, Q.; Szeler, K.; Kamerlin, S. C. L.; Tawfik, D. S. Enzyme Evolution: An Epistatic Ratchet versus a Smooth Reversible Transition. *Mol. Biol. Evol.* **2020**, *37*, 1133–1147.

(20) Liao, Q.; Kulkarni, Y.; Sengupta, U.; Petrović, D.; Mulholland, A. J.; van der Kamp, M. W.; Strodel, B.; Kamerlin, S. C. L. Loop Motion in Triosephosphate Isomerase Is Not a Simple Open and Shut Case. *J. Am. Chem. Soc.* **2018**, *140*, 15889–15903.

(21) Purg, M.; Elias, M.; Kamerlin, S. C. L. Similar Active Sites and Mechanisms Do Not Lead to Cross-Promiscuity in Organophosphate Hydrolysis: Implications for Biotherapeutic

Engineering. *J. Am. Chem. Soc.* **2017**, *139*, 17533–17546.

(22) Barrozo, A.; Duarte, F.; Bauer, P.; Carvalho, A. T. P.; Kamerlin, S. C. L. Cooperative Electrostatic Interactions Drive Functional Evolution in the Alkaline Phosphatase Superfamily. *J. Am. Chem. Soc.* **2015**, *137*, 9061–9076.

(23) Jorgensen, W. L.; Chandrasekhar, J.; Madura, J. D.; Impey, R. W.; Klein, M. L. Comparison of Simple Potential Functions for Simulating Liquid Water. *J. Chem. Phys.* **1983**, *79*, 926–935.

(24) Ryckaert, J.-P.; Ciccotti, G.; Berendsen, H. J. C. Numerical Integration of the Cartesian Equations of Motion of a System with Constraints: Molecular Dynamics of *n*-Alkanes. *J. Comput. Phys.* **1977**, *23*, 327–341.

(25) Schneider, T.; Stoll, E. Molecular-Dynamics Study of a Three-Dimensional One-Component Model for Distortive Phase Transitions. *Phys. Rev. B* **1978**, *17*, 1302–1322.

(26) Berendsen, H. J. C.; Postma, J. P. M.; van Gunsteren, W. F.; DiNola, A.; Haak, J. R. Molecular Dynamics with Coupling to an External Bath. *J. Chem. Phys.* **1984**, *81*, 3684–3690.

(27) Darden, T.; York, D.; Pedersen, L. Particle Mesh Ewald: An $N \cdot \log(N)$ Method for Ewald Sums in Large Systems. *J. Chem. Phys.* **1993**, *98*, 10089–10092.

(28) Bussi, G. Hamiltonian Replica Exchange in GROMACS: A Flexible Implementation. *Mol. Phys.* **2014**, *112*, 379–384.

(29) Parrinello, M.; Rahman, A. Polymorphic Transitions in Single Crystals: A New Molecular Dynamics Method. *J. Appl. Phys.* **1981**, *52*, 7182–7190.

(30) Hess, B. P-LINCS: A Parallel Linear Constraint Solver for Molecular Simulation. *J. Chem.*

Theory Comput. **2008**, *4*, 116–122.

(31) Meli, M.; Colombo, G. A Hamiltonian Replica Exchange Molecular Dynamics (MD) Method for the Study of Folding, Based on the Analysis of the Stabilization Determinants of Proteins. *Int. J. Mol. Sci.* **2013**, *14*, 12157–12169.

(32) Lindorff-Larsen, K.; Piana, S.; Palmo, K.; Maragakis, P.; Klepeis, J. L.; Dror, R. O.; Shaw, D. E. Improved Side-Chain Torsion Potentials for the Amber ff99SB Protein Force Field. *Proteins Struct. Funct. Bioinformat.* **2010**, *78*, 1950–1958.

(33) Van Der Spoel, D.; Lindahl, E.; Hess, B.; Groenhof, G.; Mark, A. E.; Berendsen, H. J. C. GROMACS: Fast, Flexible, and Free. *J. Comput. Chem.* **2005**, *26*, 1701–1718.

(34) Tribello, G. A.; Bonomi, M.; Branduardi, D.; Camilloni, C.; Bussi, G. PLUMED 2: New Feathers for an Old Bird. *Comput. Phys. Commun.* **2014**, *185*, 604–613.

(35) Bonomi, M.; Parrinello, M. Enhanced Sampling in the Well-Tempered Ensemble. *Phys. Rev. Lett.* **2010**, *104*, 190601.

(36) Deighan, M.; Bonomi, M.; Pfaendtner, J. Efficient Simulation of Explicitly Solvated Proteins in the Well-Tempered Ensemble. *J. Chem. Theory Comput.* **2012**, *8*, 2189–2192.

(37) Lovera, S.; Morando, M.; Pucheta-Martinez, E.; Martinez-Torrecuadrada, J. L.; Saladino, G.; Gervasio, F. L. Towards a Molecular Understanding of the Link between Imatinib Resistance and Kinase Conformational Dynamics. *PLOS Comput. Biol.* **2015**, *11*, e1004578.

(38) Lovera, S.; Sutto, L.; Boubeva, R.; Scapozza, L.; Dölker, N.; Gervasio, F. L. The Different Flexibility of C-Src and c-Abl Kinases Regulates the Accessibility of a Druggable Inactive Conformation. *J. Am. Chem. Soc.* **2012**, *134*, 2496–2499.

(39) Doro, F.; Saladino, G.; Belvisi, L.; Civera, M.; Gervasio, F. L. New Insights into the Molecular Mechanism of E-Cadherin-Mediated Cell Adhesion by Free Energy Calculations. *J. Chem. Theory Comput.* **2015**, *11*, 1354–1359.

(40) Barducci, A.; Bussi, G.; Parrinello, M. Well-Tempered Metadynamics: A Smoothly Converging and Tunable Free-Energy Method. *Phys. Rev. Lett.* **2008**, *100*, 020603.

(41) Barducci, A.; Bonomi, M.; Prakash, M. K.; Parrinello, M. Free-Energy Landscape of Protein Oligomerization from Atomistic Simulations. *Proc. Natl. Acad. Sci. USA* **2013**, *110*, E4708–E4713.

(42) Tiwary, P.; Parrinello, M. A Time-Independent Free Energy Estimator for Metadynamics. *J. Phys. Chem. B* **2015**, *119*, 736–742.

(43) Marcos-Alcalde, I.; Setoain, J.; Mendieta-Moreno, J. I.; Mendieta, J.; Gómez-Puertas, P. MEPSA: Minimum Energy Pathway Analysis for Energy Landscapes. *Bioinformatics* **2015**, btv453.

(44) Romero-Rivera, A.; Garcia-Borràs, M.; Osuna, S. Role of Conformational Dynamics in the Evolution of Retro-Aldolase Activity. *ACS Catal.* **2017**, *7*, 8524–8532.

(45) Best, R. B.; Hummer, G.; Eaton, W. A. Native Contacts Determine Protein Folding Mechanisms in Atomistic Simulations. *Proc. Natl. Acad. Sci. USA* **2013**, *110*, 17874–17879.

(46) Warshel, A.; Weiss, R. M. An Empirical Valence Bond Approach for Comparing Reactions in Solutions and in Enzymes. *J. Am. Chem. Soc.* **1980**, *102*, 6218–6226.

(47) Warshel, A.; King, G. Polarization Constraints in Molecular Dynamics Simulation of Aqueous Solutions: The Surface Constraint All Atom Solvent (SCAAS) Model. *Chem.*

Phys. Lett. **1985**, *121*, 124–129.

(48) Kulkarni, Y. S.; Amyes, T. L.; Richard, J. P.; Kamerlin, S. C. L. Uncovering the Role of Key Active-Site Side Chains in Catalysis: An Extended Brønsted Relationship for Substrate Deprotonation Catalyzed by Wild-Type and Variants of Triosephosphate Isomerase. *J. Am. Chem. Soc.* **2019**, *141*, 16139–16150.

(49) Calixto, A. R.; Moreira, C.; Pabis, A.; Kötting, C.; Gerwert, K.; Rudack, T.; Kamerlin, S. C. L. GTP Hydrolysis Without an Active Site Base: A Unifying Mechanism for Ras and Related GTPases. *J. Am. Chem. Soc.* **2019**, *141*, 10684–10701.

(50) Blaha-Nelson, D.; Krüger, D. M.; Szeler, K.; Ben-David, M.; Kamerlin, S. C. L. Active Site Hydrophobicity and the Convergent Evolution of Paraoxonase Activity in Structurally Divergent Enzymes: The Case of Serum Paraoxonase 1. *J. Am. Chem. Soc.* **2017**, *139*, 1155–1167.

(51) Shurki, A.; Derat, E.; Barrozo, A.; Kamerlin, S. C. L. How Valence Bond Theory Can Help You Understand Your (Bio)Chemical Reaction. *Chem. Soc. Rev.* **2015**, *44*, 1037–1052.

(52) Rosta, E.; Warshel, A. Origin of Linear Free Energy Relationships: Exploring the Nature of the Off-Diagonal Coupling Elements in S_N2 Reactions. *J. Chem. Theory Comput.* **2012**, *8*, 3574–3585.

(53) Hong, G.; Rosta, E.; Warshel, A. Using the Constrained DFT Approach in Generating Diabatic Surfaces and Off Diagonal Empirical Valence Bond Terms for Modeling Reactions in Condensed Phases. *J. Phys. Chem. B* **2006**, *110*, 19570–19574.

(54) Kirby, A. J.; Jencks, W. P. The Reactivity of Nucleophilic Reagents toward the *P*-Nitrophenyl Phosphate Dianion. *J. Am. Chem. Soc.* **1965**, *87*, 3209–3216.

(55) Milstien, S.; Fife, T. H. Hydrolysis of S-Aryl Phosphorothioates. *J. Am. Chem. Soc.* **1967**, *89*, 5820–5826.

(56) Fife, T. H.; Milstien, S. Carboxyl Group Participation in Phosphorothioate Hydrolysis. Hydrolysis of S-(2-Carboxyphenyl)Phosphorothioate. *J. Org. Chem.* **1969**, *34*, 4007–4012.

(57) Duarte, F.; Åqvist, J.; Williams, N. H.; Kamerlin, S. C. L. Resolving Apparent Conflicts between Theoretical and Experimental Models of Phosphate Monoester Hydrolysis. *J. Am. Chem. Soc.* **2015**, *137*, 1081–1093.

(58) Zhao, Y.; Truhlar, D. G. The M06 Suite of Density Functionals for Main Group Thermochemistry, Thermochemical Kinetics, Noncovalent Interactions, Excited States, and Transition Elements: Two New Functionals and Systematic Testing of Four M06-Class Functionals and 12 Other Functionals. *Theor. Chem. Acc.* **2008**, *120*, 215–241.

(59) Frisch, M. J.; Trucks, G. W.; Schlegel, H. B.; Scuseria, G. E.; Robb, M. A.; Cheeseman, J. R.; Scalmani, G.; Barone, V.; Petersson, G. A.; Nakatsuji, H.; Li, X.; Caricato, M.; Fox, D. J.; *et al*. Gaussian 09, Revision E.02. Gaussian, Inc.: Wallingford CT 2009.

(60) Barrozo, A.; Blaha-Nelson, D.; Williams, N. H.; Kamerlin, S. C. L. The Effect of Magnesium Ions on Triphosphate Hydrolysis. *Pure Appl. Chem.* **2017**, *89*, 715–727.

(61) Marenich, A. V.; Cramer, C. J.; Truhlar, D. G. Universal Solvation Model Based on Solute Electron Density and on a Continuum Model of the Solvent Defined by the Bulk Dielectric Constant and Atomic Surface Tensions. *J. Phys. Chem. B* **2009**, *113*, 6378–6396.

(62) Hratchian, H. P.; Schlegel, H. B. Accurate Reaction Paths Using a Hessian Based Predictor–Corrector Integrator. *J. Chem. Phys.* **2004**, *120*, 9918–9924.

(63) Hratchian, H. P.; Schlegel, H. B. Using Hessian Updating To Increase the Efficiency of a

Hessian Based Predictor-Corrector Reaction Path Following Method. *J. Chem. Theory Comput.* **2005**, *1*, 61–69.

(64) Schrödinger LLC: Schrödinger Release 2017-1: MacroModel. New York, NY, 2017.

(65) Bauer, P.; Barrozo, A.; Purg, M.; Amrein, B. A.; Esguerra, M.; Wilson, P. B.; Major, D. T.; Åqvist, J.; Kamerlin, S. C. L. Q6: A Comprehensive Toolkit for Empirical Valence Bond and Related Free Energy Calculations. *SoftwareX* **2018**, *7*, 388–395.

(66) Lee, F. S.; Warshel, A. A Local Reaction Field Method for Fast Evaluation of Long-range Electrostatic Interactions in Molecular Simulations. *J. Chem. Phys.* **1992**, *97*, 3100–3107.

(67) Humphrey, W.; Dalke, A.; Schulten, K. VMD: Visual Molecular Dynamics. *J. Mol. Graph.* **1996**, *14*, 33–38.

(68) Daura, X.; Gademann, K.; Jaun, B.; Seebach, D.; van Gunsteren, W. F.; Mark, A. E. Peptide Folding: When Simulation Meets Experiment. *Angew. Chemie Int. Ed.* **1999**, *38*, 236–240.

(69) Xin, Z.; Liu, G.; Abad-Zapatero, C.; Pei, Z.; Szczepankiewicz, B. G.; Li, X.; Zhang, T.; Hutchins, C. W.; Hajduk, P. J.; Ballaron, S. J.; Stashko, M. A.; Lubben, T. H.; Trevillyan, J. M.; Jirousek, M. R. Identification of a Monoacid-Based, Cell Permeable, Selective Inhibitor of Protein Tyrosine Phosphatase 1B. *Bioorg. Med. Chem. Lett.* **2003**, *13*, 3947–3950.

(70) Wiesmann, C.; Barr, K. J.; Kung, J.; Zhu, J.; Erlanson, D. A.; Shen, W.; Fahr, B. J.; Zhong, M.; Taylor, L.; Randal, M.; McDowell, R. S.; Hansen, S. K. Allosteric Inhibition of Protein Tyrosine Phosphatase 1B. *Nat. Struct. Mol. Biol.* **2004**, *11*, 730–737.

(71) Guan, K.; Dixon, J. Protein Tyrosine Phosphatase Activity of an Essential Virulence Determinant in Yersinia. *Science* **1990**, *249*, 553–556.

(72) Brubaker, R. R. Factors Promoting Acute and Chronic Diseases Caused by Yersiniae. *Clin. Microbiol. Rev.* **1991**, *4*, 309–324.

(73) Kuban-Jankowska, A.; Sahu, K. K.; Gorska, M.; Tuszynski, J. A.; Wozniak, M. Chicoric Acid Binds to Two Sites and Decreases the Activity of the YopH Bacterial Virulence Factor. *Oncotarget* **2016**, *7*, 2229–2238.

(74) Cui, D. S.; Beaumont, V.; Ginther, P. S.; Lipchock, J. M.; Loria, J. P. Leveraging Reciprocity to Identify and Characterize Unknown Allosteric Sites in Protein Tyrosine Phosphatases. *J. Mol. Biol.* **2017**, *429*, 2360–2372.

(75) Hjortness, M. K.; Riccardi, L.; Hongdusit, A.; Zwart, P. H.; Sankaran, B.; De Vivo, M.; Fox, J. M. Evolutionarily Conserved Allosteric Communication in Protein Tyrosine Phosphatases. *Biochemistry* **2018**, *57*, 6443–6451.

(76) Whittier, S. K.; Hengge, A. C.; Loria, J. P. Conformational Motions Regulate Phosphoryl Transfer in Related Protein Tyrosine Phosphatases. *Science* **2013**, *341*, 899–903.

(77) Ester, M.; Kriegel, H.-P.; Sander, J.; Xu, X. A Density-Based Algorithm for Discovering Clusters in Large Spatial Databases with Noise. *Data Min. Knowl. Discov.*, **1996**, 90, 226–231.

(78) Zhang, Z. Y.; Malachowski, W. P.; Etten, R. L. Van; Dixon, J. E. Nature of the Rate-Determining Steps of the Reaction Catalyzed by the Yersinia Protein-Tyrosine Phosphatase. *J. Biol. Chem.* **1994**, *269*, 8140–8145.

(79) Balakrishnan, S.; Kamisetty, H.; Carbonell, J. G.; Lee, S.-I.; Langmead, C. J. Learning Generative Models for Protein Fold Families. *Proteins Struct. Funct. Bioinformat.* **2011**, *79*, 1061–1078.

(80) Li, L.; Li, C.; Zhang, Z.; Alexov, E. On the Dielectric "Constant" of Proteins: Smooth Dielectric Function for Macromolecular Modeling and Its Implementation in DelPhi. *J. Chem. Theory Comput.* **2013**, *9*, 2126–2136.

(81) Kulkarni, Y. S.; Liao, Q.; Petrović, D.; Krüger, D. M.; Strodel, B.; Amyes, T. L.; Richard, J. P.; Kamerlin, S. C. L. Enzyme Architecture: Modeling the Operation of a Hydrophobic Clamp in Catalysis by Triosephosphate Isomerase. *J. Am. Chem. Soc.* **2017**, *139*, 10514–10525.

(82) Puius, Y. A.; Zhao, Y.; Sullivan, M.; Lawrence, D. S.; Almo, S. C.; Zhang, Z. Y. Identification of a Second Aryl Phosphate-Binding Site in Protein-Tyrosine Phosphatase 1B: A Paradigm for Inhibitor Design. *Proc. Natl. Acad. Sci. USA* **1997**, *94*, 13420–13425.

(83) Groves, M. R.; Yao, Z. J.; Roller, P. P.; Burke, T. R.; Barford, D. Structural Basis for Inhibition of the Protein Tyrosine Phosphatase 1B by Phosphotyrosine Peptide Mimetics. *Biochemistry* **1998**, *37*, 17773–17783.

(84) Andersen, H. S.; Iversen, L. F.; Jeppesen, C. B.; Branner, S.; Norris, K.; Rasmussen, H. B.; Møller, K. B.; Møller, N. P. 2-(Oxalylamino)-Benzoic Acid Is a General, Competitive Inhibitor of Protein-Tyrosine Phosphatases. *J. Biol. Chem.* **2000**, *275*, 7101–7108.

(85) Iversen, L. F.; Andersen, H. S.; Branner, S.; Mortensen, S. B.; Peters, G. H.; Norris, K.; Olsen, O. H.; Jeppesen, C. B.; Lundt, B. F.; Ripka, W.; Møller, K. B.; Møller, N. P. Structure-Based Design of a Low Molecular Weight, Nonphosphorus, Nonpeptide, and Highly Selective Inhibitor of Protein-Tyrosine Phosphatase 1B. *J. Biol. Chem.* **2000**, *275*, 10300–10307.

(86) Sarmiento, M.; Puius, Y. A.; Vetter, S. W.; Keng, Y. F.; Wu, L.; Zhao, Y.; Lawrence, D.

S.; Almo, S. C.; Zhang, Z. Y. Structural Basis of Plasticity in Protein Tyrosine Phosphatase 1B Substrate Recognition. *Biochemistry* **2000**, *39*, 8171–8179.

(87) Salmeen, A.; Andersen, J. N.; Myers, M. P.; Tonks, N. K.; Barford, D. Molecular Basis for the Dephosphorylation of the Activation Segment of the Insulin Receptor by Protein Tyrosine Phosphatase 1B. *Mol. Cell* **2000**, *6*, 1401–1412.

(88) Bleasdale, J. E.; Ogg, D.; Palazuk, B. J.; Jacob, C. S.; Swanson, M. L.; Wang, X. Y.; Thompson, D. P.; Conradi, R. A.; Mathews, W. R.; Laborde, A. L.; Stuchly, C. W.; Heijbel, A.; Bergdahl, K.; Bannow, C. A.; Smith, C. W.; Svensson, C.; Liljebris, C.; Schostarez, H. J.; May, P. D.; Stevens, F. C.; Larsen, S. D. Small Molecule Peptidomimetics Containing a Novel Phosphotyrosine Bioisostere Inhibit Protein Tyrosine Phosphatase 1B and Augment Insulin Action. *Biochemistry* **2001**, *40*, 5642–5654.

(89) Peters, G. H.; Iversen, L. F.; Branner, S.; Andersen, H. S.; Mortensen, S. B.; Olsen, O. H.; Moller, K. B.; Moller, N. P. Residue 259 Is a Key Determinant of Substrate Specificity of Protein-Tyrosine Phosphatases 1B and Alpha. *J. Biol. Chem.* **2000**, *275*, 18201–18209.

(90) Scapin, G.; Patel, S.; Patel, V.; Kennedy, B.; Asante-Appiah, E. The Structure of Apo Protein-Tyrosine Phosphatase 1B C215S Mutant: More than Just an S --> O Change. *Protein Sci.* **2001**, *10*, 1596–1605.

(91) Larsen, S. D.; Barf, T.; Liljebris, C.; May, P. D.; Ogg, D.; O'Sullivan, T. J.; Palazuk, B. J.; Schostarez, H. J.; Stevens, F. C.; Bleasdale, J. E. Synthesis and Biological Activity of a Novel Class of Small Molecular Weight Peptidomimetic Competitive Inhibitors of Protein Tyrosine Phosphatase 1B. *J. Med. Chem.* **2002**, *45*, 598–622.

(92) Iversen, L. F.; Andersen, H. S.; Møller, K. B.; Olsen, O. H.; Peters, G. H.; Branner, S.;

Mortensen, S. B.; Hansen, T. K.; Lau, J.; Ge, Y.; Holsworth, D. D.; Newman, M. J.; Hundahl Møller, N. P. Steric Hindrance as a Basis for Structure-Based Design of Selective Inhibitors of Protein-Tyrosine Phosphatases. *Biochemistry* **2001**, *40*, 14812–14820.

(93) Szczepankiewicz, B. G.; Liu, G.; Hajduk, P. J.; Abad-Zapatero, C.; Pei, Z.; Xin, Z.; Lubben, T. H.; Trevillyan, J. M.; Stashko, M. A.; Ballaron, S. J.; Liang, H.; Huang, F.; Hutchins, C. W.; Fesik, S. W.; Jirousek, M. R. Discovery of a Potent, Selective Protein Tyrosine Phosphatase 1B Inhibitor Using a Linked-Fragment Strategy. *J. Am. Chem. Soc.* **2003**, *125*, 4087–4096.

(94) Erlanson, D. A.; McDowell, R. S.; He, M. M.; Randal, M.; Simmons, R. L.; Kung, J.; Waight, A.; Hansen, S. K. Discovery of a New Phosphotyrosine Mimetic for PTP1B Using Breakaway Tethering. *J. Am. Chem. Soc.* **2003**, *125*, 5602–5603.

(95) Xin, Z.; Oost, T. K.; Abad-Zapatero, C.; Hajduk, P. J.; Pei, Z.; Szczepankiewicz, B. G.; Hutchins, C. W.; Ballaron, S. J.; Stashko, M. A.; Lubben, T.; Trevillyan, J. M.; Jirousek, M. R.; Liu, G. Potent, Selective Inhibitors of Protein Tyrosine Phosphatase 1B. *Bioorg. Med. Chem. Lett.* **2003**, *13*, 1887–1890.

(96) Salmeen, A.; Andersen, J. N.; Myers, M. P.; Meng, T.-C.; Hinks, J. A.; Tonks, N. K.; Barford, D. Redox Regulation of Protein Tyrosine Phosphatase 1B Involves a Sulphenyl-Amide Intermediate. *Nature* **2003**, *423*, 769–773.

(97) Liu, G.; Szczepankiewicz, B. G.; Pei, Z.; Janowick, D. A.; Xin, Z.; Hajduk, P. J.; Abad-Zapatero, C.; Liang, H.; Hutchins, C. W.; Fesik, S. W.; Ballaron, S. J.; Stashko, M. A.; Lubben, T.; Mika, A. K.; Zinker, B. A.; Trevillyan, J. M.; Jirousek, M. R. Discovery and Structure-Activity Relationship of Oxalylarylaminobenzoic Acids as Inhibitors of Protein

Tyrosine Phosphatase 1B. *J. Med. Chem.* **2003**, *46*, 2093–2103.

(98) Liu, G.; Xin, Z.; Liang, H.; Abad-Zapatero, C.; Hajduk, P. J.; Janowick, D. A.; Szczepankiewicz, B. G.; Pei, Z.; Hutchins, C. W.; Ballaron, S. J.; Stashko, M. A.; Lubben, T. H.; Berg, C. E.; Rondinone, C. M.; Trevillyan, J. M.; Jirousek, M. R. Selective Protein Tyrosine Phosphatase 1B Inhibitors: Targeting the Second Phosphotyrosine Binding Site with Non-Carboxylic Acid-Containing Ligands. *J. Med. Chem.* **2003**, *46*, 3437–3440.

(99) Sun, J.-P.; Fedorov, A. A.; Lee, S.-Y.; Guo, X.-L.; Shen, K.; Lawrence, D. S.; Almo, S. C.; Zhang, Z.-Y. Crystal Structure of PTP1B Complexed with a Potent and Selective Bidentate Inhibitor. *J. Biol. Chem.* **2003**, *278*, 12406–12414.

(100) Pei, Z.; Li, X.; Liu, G.; Abad-Zapatero, C.; Lubben, T.; Zhang, T.; Ballaron, S. J.; Hutchins, C. W.; Trevillyan, J. M.; Jirousek, M. R. Discovery and SAR of Novel, Potent and Selective Protein Tyrosine Phosphatase 1B Inhibitors. *Bioorg. Med. Chem. Lett.* **2003**, *13*, 3129–3132.

(101) Liu, G.; Xin, Z.; Pei, Z.; Hajduk, P. J.; Abad-Zapatero, C.; Hutchins, C. W.; Zhao, H.; Lubben, T. H.; Ballaron, S. J.; Haasch, D. L.; Kaszubska, W.; Rondinone, C. M.; Trevillyan, J. M.; Jirousek, M. R. Fragment Screening and Assembly: A Highly Efficient Approach to a Selective and Cell Active Protein Tyrosine Phosphatase 1B Inhibitor. *J. Med. Chem.* **2003**, *46*, 4232–4235.

(102) Scapin, G.; Patel, S. B.; Becker, J. W.; Wang, Q.; Desponts, C.; Waddleton, D.; Skorey, K.; Cromlish, W.; Bayly, C.; Therien, M.; Gauthier, J. Y.; Li, C. S.; Lau, C. K.; Ramachandran, C.; Kennedy, B. P.; Asante-Appiah, E. The Structural Basis for the Selectivity of Benzotriazole Inhibitors of PTP1B. *Biochemistry* **2003**, *42*, 11451–11459.

(103) Pedersen, A. K.; Peters G, G. üNther H.; Møller, K. B.; Iversen, L. F.; Kastrup, J. S. Water-Molecule Network and Active-Site Flexibility of Apo Protein Tyrosine Phosphatase 1B. *Acta Crystallogr. D. Biol. Crystallogr.* **2004**, *60*, 1527–1534.

(104) Zhao, H.; Liu, G.; Xin, Z.; Serby, M. D.; Pei, Z.; Szczepankiewicz, B. G.; Hajduk, P. J.; Abad-Zapatero, C.; Hutchins, C. W.; Lubben, T. H.; Ballaron, S. J.; Haasch, D. L.; Kaszubska, W.; Rondinone, C. M.; Trevillyan, J. M.; Jirousek, M. R. Isoxazole Carboxylic Acids as Protein Tyrosine Phosphatase 1B (PTP1B) Inhibitors. *Bioorg. Med. Chem. Lett.* **2004**, *14*, 5543–5546.

(105) Moretto, A. F.; Kirincich, S. J.; Xu, W. X.; Smith, M. J.; Wan, Z.-K.; Wilson, D. P.; Follows, B. C.; Binnun, E.; Joseph-McCarthy, D.; Foreman, K.; Erbe, D. V; Zhang, Y. L.; Tam, S. K.; Tam, S. Y.; Lee, J. Bicyclic and Tricyclic Thiophenes as Protein Tyrosine Phosphatase 1B Inhibitors. *Bioorg. Med. Chem.* **2006**, *14*, 2162–2177.

(106) Black, E.; Breed, J.; Breeze, A. L.; Embrey, K.; Garcia, R.; Gero, T. W.; Godfrey, L.; Kenny, P. W.; Morley, A. D.; Minshull, C. A.; Pannifer, A. D.; Read, J.; Rees, A.; Russell, D. J.; Toader, D.; Tucker, J. Structure-Based Design of Protein Tyrosine Phosphatase-1B Inhibitors. *Bioorg. Med. Chem. Lett.* **2005**, *15*, 2503–2507.

(107) Montalibet, J.; Skorey, K.; McKay, D.; Scapin, G.; Asante-Appiah, E.; Kennedy, B. P. Residues Distant from the Active Site Influence Protein-Tyrosine Phosphatase 1B Inhibitor Binding. *J. Biol. Chem.* **2006**, *281*, 5258–5266.

(108) Klopfenstein, S. R.; Evdokimov, A. G.; Colson, A.-O.; Fairweather, N. T.; Neuman, J. J.; Maier, M. B.; Gray, J. L.; Gerwe, G. S.; Stake, G. E.; Howard, B. W.; Farmer, J. A.; Pokross, M. E.; Downs, T. R.; Kasibhatla, B.; Peters, K. G. 1,2,3,4-Tetrahydroisoquinolinyl Sulfamic

Acids as Phosphatase PTP1B Inhibitors. *Bioorg. Med. Chem. Lett.* **2006**, *16*, 1574–1578.

(109) Asante-Appiah, E.; Patel, S.; Desponts, C.; Taylor, J. M.; Lau, C.; Dufresne, C.; Therien, M.; Friesen, R.; Becker, J. W.; Leblanc, Y.; Kennedy, B. P.; Scapin, G. Conformation-Assisted Inhibition of Protein-Tyrosine Phosphatase-1B Elicits Inhibitor Selectivity over T-Cell Protein-Tyrosine Phosphatase. *J. Biol. Chem.* **2006**, *281*, 8010–8015.

(110) Wan, Z.-K.; Lee, J.; Xu, W.; Erbe, D. V; Joseph-McCarthy, D.; Follows, B. C.; Zhang, Y.-L. Monocyclic Thiophenes as Protein Tyrosine Phosphatase 1B Inhibitors: Capturing Interactions with Asp48. *Bioorg. Med. Chem. Lett.* **2006**, *16*, 4941–4945.

(111) Wan, Z.-K.; Follows, B.; Kirincich, S.; Wilson, D.; Binnun, E.; Xu, W.; Joseph-McCarthy, D.; Wu, J.; Smith, M.; Zhang, Y.-L.; Tam, M.; Erbe, D.; Tam, S.; Saiah, E.; Lee, J. Probing Acid Replacements of Thiophene PTP1B Inhibitors. *Bioorg. Med. Chem. Lett.* **2007**, *17*, 2913–2920.

(112) Wilson, D. P.; Wan, Z.-K.; Xu, W.-X.; Kirincich, S. J.; Follows, B. C.; Joseph-McCarthy, D.; Foreman, K.; Moretto, A.; Wu, J.; Zhu, M.; Binnun, E.; Zhang, Y.-L.; Tam, M.; Erbe, D. V.; Tobin, J.; Xu, X.; Leung, L.; Shilling, A.; Tam, S. Y.; Mansour, T. S.; Lee, J. Structure-Based Optimization of Protein Tyrosine Phosphatase 1B Inhibitors: From the Active Site to the Second Phosphotyrosine Binding Site. *J. Med. Chem.* **2007**, *50*, 4681–4698.

(113) Douty, B.; Wayland, B.; Ala, P. J.; Bower, M. J.; Pruitt, J.; Bostrom, L.; Wei, M.; Klabe, R.; Gonneville, L.; Wynn, R.; Burn, T. C.; Liu, P. C. C.; Combs, A. P.; Yue, E. W. Isothiazolidinone Inhibitors of PTP1B Containing Imidazoles and Imidazolines. *Bioorg. Med. Chem. Lett.* **2008**, *18*, 66–71.

(114) Wan, Z.-K.; Lee, J.; Hotchandani, R.; Moretto, A.; Binnun, E.; Wilson, D. P.; Kirincich, S. J.; Follows, B. C.; Ipek, M.; Xu, W.; Joseph-McCarthy, D.; Zhang, Y.-L.; Tam, M.; Erbe, D. V.; Tobin, J. F.; Li, W.; Tam, S. Y.; Mansour, T. S.; Wu, J. Structure-Based Optimization of Protein Tyrosine Phosphatase-1 B Inhibitors: Capturing Interactions with Arginine 24. *ChemMedChem* **2008**, *3*, 1525–1529.

(115) Abdo, M.; Liu, S.; Zhou, B.; Walls, C. D.; Wu, L.; Knapp, S.; Zhang, Z.-Y. Seleninate in Place of Phosphate: Irreversible Inhibition of Protein Tyrosine Phosphatases. *J. Am. Chem. Soc.* **2008**, *130*, 13196–13197.

(116) Li, H.; Yao, X.-Q.; Grant, B. J. Comparative Structural Dynamic Analysis of GTPases. *PLoS Comput. Biol.* **2018**, *14*, e1006364.

(117) Brandão, T. A. S.; Johnson, S. J.; Hengge, A. C. The Molecular Details of WPD-Loop Movement Differ in the Protein-Tyrosine Phosphatases YopH and PTP1B. *Arch. Biochem. Biophys.* **2012**, *525*, 53–59.

(118) Zhou, H.; Singh, H.; Parsons, Z. D.; Lewis, S. M.; Bhattacharya, S.; Seiner, D. R.; LaButti, J. N.; Reilly, T. J.; Tanner, J. J.; Gates, K. S. The Biological Buffer Bicarbonate/CO2 Potentiates H2O2-Mediated Inactivation of Protein Tyrosine Phosphatases. *J. Am. Chem. Soc.* **2011**, *133*, 15803–15805.

(119) Haque, A.; Andersen, J. N.; Salmeen, A.; Barford, D.; Tonks, N. K. Conformation-Sensing Antibodies Stabilize the Oxidized Form of PTP1B and Inhibit Its Phosphatase Activity. *Cell* **2011**, *147*, 185–198.

(120) Kenny, P. W.; Newman, J.; Peat, T. S. Nitrate in the Active Site of Protein Tyrosine Phosphatase 1B Is a Putative Mimetic of the Transition State. *Acta Crystallogr. Sect. D*

Biol. Crystallogr. **2014**, *70*, 565–571.

(121) Reddy, M. V. V. S.; Ghadiyaram, C.; Panigrahi, S. K.; Krishnamurthy, N. R.; Hosahalli, S.; Chandrasekharappa, A. P.; Manna, D.; Badiger, S. E.; Dubey, P. K.; Mangamoori, L. N. X-Ray Structure of PTP1B in Complex with a New PTP1B Inhibitor. *Protein Pept. Lett.* **2014**, *21*, 90–93.

(122) Xiao, P.; Wang, X.; Wang, H.-M.; Fu, X.-L.; Cui, F.; Yu, X.; Wen, S.; Bi, W.-X.; Sun, J.-P. The Second-Sphere Residue T263 Is Important for the Function and Catalytic Activity of PTP1B via Interaction with the WPD-Loop. *Int. J. Biochem. Cell Biol.* **2014**, *57*, 84–95.

(123) Selner, N. G.; Luechapanichkul, R.; Chen, X.; Neel, B. G.; Zhang, Z.-Y.; Knapp, S.; Bell, C. E.; Pei, D. Diverse Levels of Sequence Selectivity and Catalytic Efficiency of Protein-Tyrosine Phosphatases. *Biochemistry* **2014**, *53*, 397–412.

(124) Choy, M. S.; Li, Y.; Machado, L. E. S. F.; Kunze, M. B. A.; Connors, C. R.; Wei, X.; Lindorff-Larsen, K.; Page, R.; Peti, W. Conformational Rigidity and Protein Dynamics at Distinct Timescales Regulate PTP1B Activity and Allostery. *Mol. Cell* **2017**, *65*, 644-658.e5.

(125) Punthasee, P.; Laciak, A. R.; Cummings, A. H.; Ruddraraju, K. V.; Lewis, S. M.; Hillebrand, R.; Singh, H.; Tanner, J. J.; Gates, K. S. Covalent Allosteric Inactivation of Protein Tyrosine Phosphatase 1B (PTP1B) by an Inhibitor-Electrophile Conjugate. *Biochemistry* **2017**, *56*, 2051–2060.

(126) Ivanov, M. I.; Stuckey, J. A.; Schubert, H. L.; Saper, M. A.; Bliska, J. B. Two Substrate-Targeting Sites in the Yersinia Protein Tyrosine Phosphatase Co-Operate to Promote Bacterial Virulence. *Mol. Microbiol.* **2005**, *55*, 1346–1356.

(127) Liu, S.; Zhou, B.; Yang, H.; He, Y.; Jiang, Z.-X.; Kumar, S.; Wu, L.; Zhang, Z.-Y. Aryl Vinyl Sulfonates and Sulfones as Active Site-Directed and Mechanism-Based Probes for Protein Tyrosine Phosphatases. *J. Am. Chem. Soc.* **2008**, *130*, 8251–8260.

(128) Brandão, T. A. S.; Robinson, H.; Johnson, S. J.; Hengge, A. C. Impaired Acid Catalysis by Mutation of a Protein Loop Hinge Residue in a YopH Mutant Revealed by Crystal Structures. *J. Am. Chem. Soc.* **2009**, *131*, 778–786.

(129) Ke, S.; Ho, M.-C.; Zhadin, N.; Deng, H.; Callender, R. Investigation of Catalytic Loop Structure, Dynamics, and Function Relationship of Yersinia Protein Tyrosine Phosphatase by Temperature-Jump Relaxation Spectroscopy and X-Ray Structural Determination. *J. Phys. Chem. B* **2012**, *116*, 6166–6176.

(130) Moise, G.; Gallup, N. M.; Alexandrova, A. N.; Hengge, A. C.; Johnson, S. J. Conservative Tryptophan Mutants of the Protein Tyrosine Phosphatase YopH Exhibit Impaired WPD-Loop Function and Crystallize with Divanadate Esters in Their Active Sites. *Biochemistry* **2015**, *54*, 6490–6500.

(131) Gunawardana, J.; Chan, F. C.; Telenius, A.; Woolcock, B.; Kridel, R.; Tan, K. L.; Ben-Neriah, S.; Mottok, A.; Lim, R. S.; Boyle, M.; Rogic, S.; Rimsza, L. M.; Guiter, C.; Leroy, K.; Gaulard, P.; Haioun, C.; Marra, M. A.; Savage, K. J.; Connors, J. M.; Shah, S. P.; Gascoyne, R. D.; Steidl, C. Recurrent Somatic Mutations of PTPN1 in Primary Mediastinal B Cell Lymphoma and Hodgkin Lymphoma. *Nat. Genet.* **2014**, *46*, 329–335.

(132) Kulkarni, Y. S.; Liao, Q.; Petrović, D.; Krüger, D. M.; Strodel, B.; Amyes, T. L.; Richard, J. P.; Kamerlin, S. C. L. Enzyme Architecture: Modeling the Operation of a Hydrophobic Clamp in Catalysis by Triosephosphate Isomerase. *J. Am. Chem. Soc.* **2017**, *139*, 10514–

10525.

(133) Muegge, I.; Tao, H.; Warshel, A. A Fast Estimate of Electrostatic Group Contributions to the Free Energy of Protein-Inhibitor Binding. *Protein Eng. Des. Sel.* **1997**, *10*, 1363–1372.

(134) Szeler, K.; Williams, N. H.; Hengge, A. C.; Kamerlin, S. C. L. Modeling the Alkaline Hydrolysis of Diaryl Sulfate Diesters: A Mechanistic Study. *J. Org. Chem.* **2020**, *85*, 6489–6497.

(135) Duarte, F.; Geng, T.; Marloie, G.; Al Hussain, A. O.; Williams, N. H.; Kamerlin, S. C. L. The Alkaline Hydrolysis of Sulfonate Esters: Challenges in Interpreting Experimental and Theoretical Data. *J. Org. Chem.* **2014**, *79*, 2816–2828.

(136) Kamerlin, S. C. L.; Warshel, A. The EVB as a Quantitative Tool for Formulating Simulations and Analyzing Biological and Chemical Reactions. *Faraday Discuss.* **2010**, *145*, 71–106.

S7. Cartesian Coordinates of QM Optimized Stationary Points

Reactant State

C	3.31800	1.12700	0.33400
C	4.65300	0.86100	0.09000
C	5.05600	-0.46500	-0.04300
C	4.16100	-1.52600	0.06900
C	2.82600	-1.25600	0.31900
C	2.40100	0.07400	0.44800
H	2.96300	2.14800	0.43500
H	5.37300	1.66500	-0.00300
H	4.50900	-2.54700	-0.03200
H	2.11600	-2.06600	0.43100
N	6.45500	-0.75200	-0.30200
O	7.23700	0.18600	-0.38500
O	6.79800	-1.91900	-0.42900
O	1.11600	0.39900	0.71600
P	-0.20900	-0.52200	0.12000
O	-0.28900	-1.71800	1.07200
O	0.14400	-0.86200	-1.31600
O	-1.32300	0.50900	0.29400
C	-2.78300	-3.03100	-2.55500
H	-3.35600	-2.33900	-3.18100
H	-1.74400	-3.02900	-2.90900
H	-3.19500	-4.03500	-2.70200
C	-2.85800	-2.61600	-1.09200
H	-2.39200	-1.63100	-0.99600
H	-2.25600	-3.30300	-0.48300
S	-4.57000	-2.52700	-0.42600
C	-2.41100	3.41100	-1.11300
H	-3.18600	4.09800	-1.46200
H	-2.69000	3.05600	-0.11600
H	-2.37700	2.55100	-1.78700
C	-1.04800	4.11800	-1.09300
C	-0.00400	3.10800	-0.71200
O	0.16400	3.00200	0.61100
H	0.65200	2.16100	0.77800
H	-0.80500	4.50200	-2.08600
H	-1.04800	4.94200	-0.37600
O	0.59100	2.40500	-1.50900
O	-2.48500	0.75700	2.72200
H	-2.79200	-0.18000	2.62100
H	-1.98000	0.85900	1.87700
O	-2.82800	-1.88400	2.17800

H	-3.46100	-2.03400	1.43000
H	-1.94100	-1.86000	1.74800
O	-1.94000	0.21100	-2.88300
H	-1.20000	-0.12900	-2.33200
H	-2.67700	0.29700	-2.24400
O	-3.84200	0.49900	-0.74800
H	-2.96700	0.45200	-0.28900
H	-4.23000	-0.41100	-0.61600
O	-4.62200	2.06700	1.44700
H	-4.50500	1.56800	0.61200
H	-3.93700	1.66300	2.01900
O	-0.06300	-0.49100	3.56500
H	-0.88300	0.03000	3.46400
H	-0.06500	-1.03200	2.74600

Transition State

C	-3.28800	0.96300	-0.79600
C	-4.58000	0.66800	-0.42400
C	-4.87800	-0.61400	0.05200
C	-3.88700	-1.59700	0.15500
C	-2.59500	-1.30000	-0.21900
C	-2.25500	-0.01100	-0.71000
H	-3.03800	1.95100	-1.17000
H	-5.36300	1.41400	-0.49600
H	-4.14300	-2.58300	0.52400
H	-1.81800	-2.05300	-0.15600
N	-6.22100	-0.92600	0.43700
O	-7.08700	-0.05800	0.33800
O	-6.47400	-2.05500	0.85600
O	-1.04000	0.28300	-1.08900
P	0.72500	-0.59300	-0.19500
O	0.50000	-1.85600	-1.01000
O	0.13200	-0.47600	1.18800
O	1.48300	0.57600	-0.80900
C	3.44700	-2.86000	3.25800
H	4.32400	-2.23300	3.45100
H	3.03300	-3.17300	4.22500
H	3.78600	-3.75500	2.72800
C	2.40900	-2.10400	2.44100
H	2.06500	-1.22300	2.99200
H	1.53100	-2.73800	2.27700
S	3.04300	-1.54900	0.81800
C	1.70000	4.25000	0.60500
H	2.21300	5.19600	0.79600
H	2.10300	3.81700	-0.31600
H	1.91000	3.56400	1.42900

C	0.18700	4.47200	0.48300
C	-0.46300	3.13900	0.22500
O	-0.45200	2.79200	-1.05400
H	-0.72100	1.80100	-1.12300
H	-0.21600	4.88300	1.41100
H	-0.03600	5.15300	-0.34100
O	-0.90100	2.41600	1.10900
O	3.29900	-0.45400	-2.56600
H	3.21600	-1.41700	-2.36300
H	2.74400	-0.03100	-1.86200
O	2.73300	-3.10200	-1.89500
H	3.22200	-3.03900	-1.05000
H	1.87000	-2.69700	-1.62300
O	1.59600	1.33400	2.72200
H	1.05000	0.72000	2.18000
H	2.37900	1.47600	2.15700
O	3.65000	1.47400	0.70400
H	2.87300	1.41200	0.10200
H	3.72100	0.51400	0.94800
O	5.55800	0.52900	-1.21800
H	5.02200	1.02300	-0.56900
H	4.86900	0.10400	-1.77000
O	0.62600	-0.72300	-3.59300
H	1.55200	-0.47500	-3.41000
H	0.46000	-1.34300	-2.86100

Product State

C	3.41500	1.28700	0.59700
C	4.58700	0.78200	0.06900
C	4.81500	-0.59500	0.11000
C	3.88600	-1.47600	0.66700
C	2.71300	-0.97000	1.19100
C	2.46200	0.41700	1.16800
H	3.21700	2.35300	0.58400
H	5.32700	1.44200	-0.36800
H	4.08600	-2.54000	0.68000
H	1.96300	-1.62500	1.62000
N	6.03900	-1.11900	-0.43800
O	6.84700	-0.34000	-0.93500
O	6.23700	-2.33000	-0.39000
O	1.33500	0.86400	1.70800
P	-1.09800	-0.70400	0.11600
O	-0.80100	-1.44200	1.42600
O	0.04300	-0.72400	-0.89400
O	-1.74600	0.68300	0.33500
C	-2.73500	-3.70300	-2.83600

H	-3.55200	-3.15800	-3.31800
H	-2.18200	-4.24200	-3.61100
H	-3.17000	-4.43800	-2.15200
C	-1.80100	-2.75400	-2.10100
H	-1.36400	-2.02400	-2.78600
H	-0.98700	-3.30400	-1.62400
S	-2.72300	-1.83000	-0.81700
C	-1.62300	3.84100	-1.41500
H	-2.16900	4.65600	-1.89600
H	-2.27100	3.40000	-0.65000
H	-1.41400	3.07100	-2.16200
C	-0.31600	4.33600	-0.79400
C	0.47100	3.16200	-0.22400
O	0.73200	3.19300	1.03100
H	1.13900	1.86000	1.43100
H	0.30100	4.81200	-1.56500
H	-0.50600	5.07000	-0.00800
O	0.80300	2.24000	-0.99500
O	-3.69300	0.32600	2.19200
H	-3.65700	-0.65700	2.26000
H	-2.97700	0.53000	1.53300
O	-3.06500	-2.34600	2.49800
H	-3.48700	-2.86900	1.79700
H	-2.18900	-2.08300	2.09700
O	-0.93200	0.59200	-3.13600
H	-0.51600	0.18500	-2.34100
H	-1.79400	0.90000	-2.79900
O	-3.43800	1.19800	-1.74900
H	-2.81200	1.13500	-0.98000
H	-3.62300	0.25500	-1.91700
O	-5.56700	0.70100	0.13900
H	-4.94300	1.04400	-0.52700
H	-4.99600	0.57200	0.92400
O	-1.81100	-0.30600	4.24800
H	-2.41600	0.12600	3.61400
H	-2.04700	-1.24200	4.14700
